# SARS-CoV-2 defective viral genomes from distinct genomic regions drive divergent interferon responses

**DOI:** 10.1371/journal.ppat.1014099

**Published:** 2026-09-23

**Authors:** Justin W. Brennan, Simone Spandau, Jiayu Xu, Xingjian Wang, Haley Aull, Gaochan Wang, Sarah Connor, Gloria S. Pryhuber, Thomas J. Mariani, Ruth Serra-Moreno, Susan R. Weiss, Yan Sun

**Affiliations:** 1 Department of Microbiology and Immunology, University of Rochester Medical Center, Rochester, New York, United States of America; 2 Department of Microbiology, Perelman School of Medicine, University of Pennsylvania, Philadelphia, Pennsylvania, United States of America; 3 Division of Allergy, Immunology, and Rheumatology (AIR), Department of Medicine, University of Rochester Medical Center, Rochester, New York, United States of America; 4 Department of Pediatrics and Center for Children’s Health Research, University of Rochester Medical Center, Rochester, New York, United States of America; 5 Departments of Pediatrics and Environmental Medicine, University of Rochester Medical Center, Rochester, New York, United States of America; Thomas Jefferson University - Center City Campus: Thomas Jefferson University, UNITED STATES OF AMERICA

## Abstract

Defective viral genomes (DVGs) are generated during the genomic replication of many RNA viruses. When produced early in infection or supplemented at the onset of infection, DVGs can attenuate viral pathogenesis by stimulating interferon responses and antagonizing wild type (WT) virus replication, highlighting their potential as antiviral therapeutics. However, during natural infection DVGs can exert both antiviral and proviral effects depending on their generation kinetics, species, and abundance, underscoring the need to better understand their roles in viral pathogenesis. Coronaviruses (CoVs) are RNA viruses that ubiquitously generate DVGs, yet the roles of DVGs during CoV infection are largely unknown. In a previous study we investigated SARS-CoV-2 DVG presence *in vitro* and in patient samples and discovered two major genomic hotspots (A and B) for their generation. Here, we first showed that overall DVG abundance tended to positively correlate with COVID-19 severity, with approximately 40% of DVGs originating from a specific genomic region designated hotspot B. Analysis of a publicly available single-cell RNA-seq dataset revealed that DVGs from hotspot B, but not hotspot A, were associated with elevated interferon responses, suggesting that DVGs derived from different genomic regions vary in their ability to stimulate innate immunity. To test this directly, we constructed two representative DVGs corresponding to hotspots A and B. Both DVGs suppressed the replication of co-infecting WT virus; however, only DVG-B induced robust interferon responses, exceeding those triggered by WT virus alone. This was further confirmed in human precision-cut lung slices. Mechanistically, DVG-B–derived dsRNA exhibited a distinct subcellular distribution compared to WT virus. Complementation with the nucleocapsid (N) partially restored dsRNA organization but did not alter the interferon response. Together, our findings demonstrate that DVGs arising from distinct genomic hotspots differentially regulate interferon responses, and N plays a unique role in regulating dsRNA distribution and interferon responses.

## Author summary

Defective viral genomes (DVGs) are produced during RNA virus infection and can suppress viral pathogenesis by stimulating innate immune responses. However, their roles in CoV infection, particularly SARS-CoV-2, remain poorly understood. This study investigated the species-specific function of DVGs generated during SARS-CoV-2 infection and their impact on disease outcomes. Cohort analysis revealed that overall DVG abundance tended to positively correlate with COVID-19 severity, with approximately 40% of DVGs originating from a specific genomic region designated hotspot B. Single-cell RNA sequencing showed that DVGs from hotspot B, but not hotspot A, were associated with elevated interferon responses, suggesting that DVGs from different genomic regions vary in their immunostimulatory capacities. To directly test this, we constructed representative DVGs from both hotspots. While both suppressed WT virus replication, only DVG-B induced robust interferon responses in both *in vitro* and *ex vivo* models. Mechanistically, DVG-B produced dsRNA with a distinct subcellular distribution compared to that of WT virus. Interestingly, complementation with the viral N protein partially restored dsRNA organization but did not alter interferon responses. These findings demonstrate that SARS-CoV-2 DVGs arising from different genomic hotspots differentially regulate innate immunity, and that N plays a unique role in regulating dsRNA distribution and interferon responses.

## Introduction

Defective viral genomes (DVGs) are generated by most RNA viruses and play important roles in viral pathogenesis [1–14]. When arising early during infection, DVGs can reduce viral pathogenicity by inducing robust type I and type III interferon (IFN) responses and competing for critical viral proteins required for genomic replication and packaging to effectively suppress wild-type (WT) virus replication [15]. For this reason, DVGs may represent a novel approach for antiviral therapeutics. However, when DVGs emerge later during infection (during and after peak viral load), their presence is associated with enhanced inflammation, higher viral titers, and greater symptom severity in patients [15]. Additionally, for some viruses, DVGs have been shown to facilitate viral persistence [16,17]. Together, these findings suggest that DVGs can have both antiviral and proviral effects depending on their generation kinetics, species, and abundance. Hence, further investigations into DVG function and their impact on viral pathogenesis are needed to harness their potential to mitigate virus-induced diseases.

Severe acute respiratory syndrome coronavirus 2 (SARS-CoV-2) is the causative agent of the coronavirus disease 2019 (COVID-19) pandemic, which resulted in extensive morbidity and mortality worldwide. Although the acute phase of the pandemic has subsided, SARS-CoV-2 continues to circulate globally and remains a significant public health concern. Continued investigation into the viral factors contributing to SARS-CoV-2 pathogenicity and how the virus interacts with host immune responses is therefore required. SARS-CoV-2 belongs to the *Betacoronavirus* genus and contains a positive-sense ~30 kb RNA genome [18]. Roughly two-thirds of the genome encodes 16 nonstructural proteins that mediate intracellular membrane remodeling and form the viral polymerase complex responsible for viral replication and transcription. The remaining one-third encodes structural and accessory proteins. Among these, the nucleocapsid (N) protein is essential for ribonucleocapsid formation and virus particle assembly [19]. Compared to influenza virus, another pandemic-causing virus, SARS-CoV-2 induces relatively weak interferon (IFN) responses *in vitro* [20]. During infection, SARS-CoV-2 generates double-stranded RNA (dsRNA) as a replicative intermediate of genomic replication and subgenomic mRNA transcription. These dsRNAs can be sensed by RIG-I–like receptors (RLRs), leading to the induction of type I and type III IFNs through mitochondrial antiviral-signaling protein (MAVS) activation [21,22]. However, viral dsRNAs are typically confined within virally generated double-membrane vesicles (DMVs), limiting their exposure to host dsRNA sensors. In addition, SARS-CoV-2 encodes multiple IFN antagonists, such as nsp1, N, and several accessory proteins, which further suppress IFN production and signaling pathways [22]. Despite these immune evasion strategies, elevated IFN responses have been observed in some COVID-19 patients, and early robust IFN responses are associated with improved clinical outcomes [23]. These observations highlight the importance of understanding the viral and host factors that regulate IFN induction during SARS-CoV-2 infection.

DVGs are ubiquitously generated during SARS-CoV-2 infection *in vitro* and in patients [17,24–28]. SARS-CoV-2 primarily generates deletion-type DVGs, which arise during viral genomic replication when the viral polymerase ceases elongation at a break point and undergoes a non-homologous recombination event wherein RNA synthesis is reinitiated at a rejoin point, deleting the sequences flanked by either side of the recombination junction. DVGs harbor internal deletions ablating essential genes required for genomic replication or virus particle assembly [29]. We previously demonstrated that the abundance of SARS-CoV-2 DVGs positively correlates with type I IFN responses. In addition, symptomatic COVID-19 patients were found to harbor higher levels of DVGs than asymptomatic individuals, suggesting that DVGs may influence IFN responses and contribute to differences in COVID-19 outcomes. Importantly, we previously identified three genomic hotspots for DVG generation, designated A, B, and C. Among these, hotspots A and B represent the two major sites contributing to the SARS-CoV-2 DVG population. Hotspot A is primarily observed *in vitro* and is defined by a rejoin point ranging from 700 to 2500 and a break point from 28,200–29,750. This hotspot contains a large deletion ablating nearly the entire viral genome, leaving the 5’ and 3’ untranslated regions (UTRs) and a small truncated fragment of nsp1. Because DVGs derived from hotspot A lack nonstructural proteins, they cannot independently replicate in the absence of SARS-CoV-2. In contrast, hotspot B is defined by a rejoin point position between 27,700 and 29,400 and a break point within the same range as hotspot A (28,200–29,750). This hotspot eliminates orf7a/7b, orf8, and N. Because N is essential for virus particle assembly and all 16 nonstructural proteins are intact, hotspot B DVGs are capable of independent genomic replication but are deficient in virus particle assembly. Moreover, hotspot B is the most conserved among the three hotspots and is detected both *in vitro* and in samples from COVID-19 patients [17].

Here, we aimed to examine the functions of DVGs from these two major hotspots, A and B, with a focus on their ability to induce IFN and suppress WT virus replication. Using a publicly available cohort, we found that total DVG abundance tended to positively correlate with COVID-19 severity. Consistently, approximately 40% of those DVGs originated from hotspot B. We then utilized a single-cell (sc) RNA-seq dataset from *in vitro* infections and observed that DVGs in hotspot B, but not hotspot A, were associated with elevated IFN responses, suggesting that DVGs from different genomic regions differ in their ability to stimulate IFN responses. To directly test this, we constructed two representative DVGs using the outermost recombination boundaries in hotspots A (DVG-A) and B (DVG-B) to maximally encompass the deletions observed in these two DVG populations. Functional analyses indicated that both DVGs suppressed the replication of co-infecting WT virus, while only DVG-B induced robust IFN responses, significantly greater than the level induced by the WT virus alone. This observation was further verified in human precision-cut lung slices (PCLS) from four different adult donors. Mechanistically, DVG-B exhibited a distinct subcellular distribution of dsRNA compared to the WT virus. Interestingly, complementation of N partially restored DVG-B’s dsRNA pattern to that of WT. This, however, did not correspondingly reduce the IFN response induced during DVG-B infection. Deletion of the N gene alone in SARS-CoV-2 induced IFN responses comparable to those elicited by DVG-B, strongly suggesting that a defect in N expression is primarily responsible for DVG-B’s IFN-induction activity. Taken together, these data indicate that DVG-A and DVG-B differ in their IFN induction capacities and that the N protein plays critical roles in dsRNA/DMV organization, IFN regulation, and virus particle assembly.

## Results

### SARS-CoV-2 DVGs tended to positively correlate with COVID-19 severity

We first sought to examine whether DVGs impact infection outcomes in COVID-19 patients. We obtained one published scRNA-seq dataset of nasal swabs collected from a COVID-19 patient cohort with known severity scores and age information (SCP1289 [30]). Using our previously published pipeline [17], we observed ~35% (13/37) of COVID-19 patients were positive for DVGs (Fig 1A). No DVGs were identified in COVID-19 negative patients. DVG+ patients had significantly higher viral read counts than DVG- patients and showed a trend toward higher severity scores. No age differences between the DVG+ and DVG- groups were observed (Fig 1B). Because age is an important factor impacting COVID-19 severity, we divided the COVID-19 patients into two age groups: younger (age < 65) and elderly (age > 65) and compared their DVG amount, viral load, and disease severity. We observed that, on average, elderly patients had significantly more total DVGs and higher severity scores than younger patients (Fig 1C). Correlation analysis of the 37 COVID-19 patients revealed a weak positive correlation between DVG abundance and disease severity scores. This trend was more evident for DVG abundance than for viral load (Fig 1D). It is important to note, however, that because DVG abundance positively correlated with viral load, the relationship between DVG abundance and disease severity may be purely correlative rather than evidence that DVGs directly contribute to disease severity. Previously, we identified two major hotspots where SARS-CoV-2 DVGs were produced, hotspot A and B, via bulk RNA-seq [17]. Hotspot A was observed primarily *in vitro*, whereas hotspot B was conserved among *in vitro* infections and lung autopsy samples from COVID-19 patients. From this cohort study, we consistently observed that hotspot B was more prevalent than hotspot A across both age groups, accounting for ~40% of the total DVG population (Fig 1E). Collectively, our analyses indicate that despite a weak correlation between SARS-CoV-2 DVGs and disease severity, hotspot B represents a dominant site of DVG generation compared with other genomic regions, warranting further mechanistic investigation.

**Fig 1:**
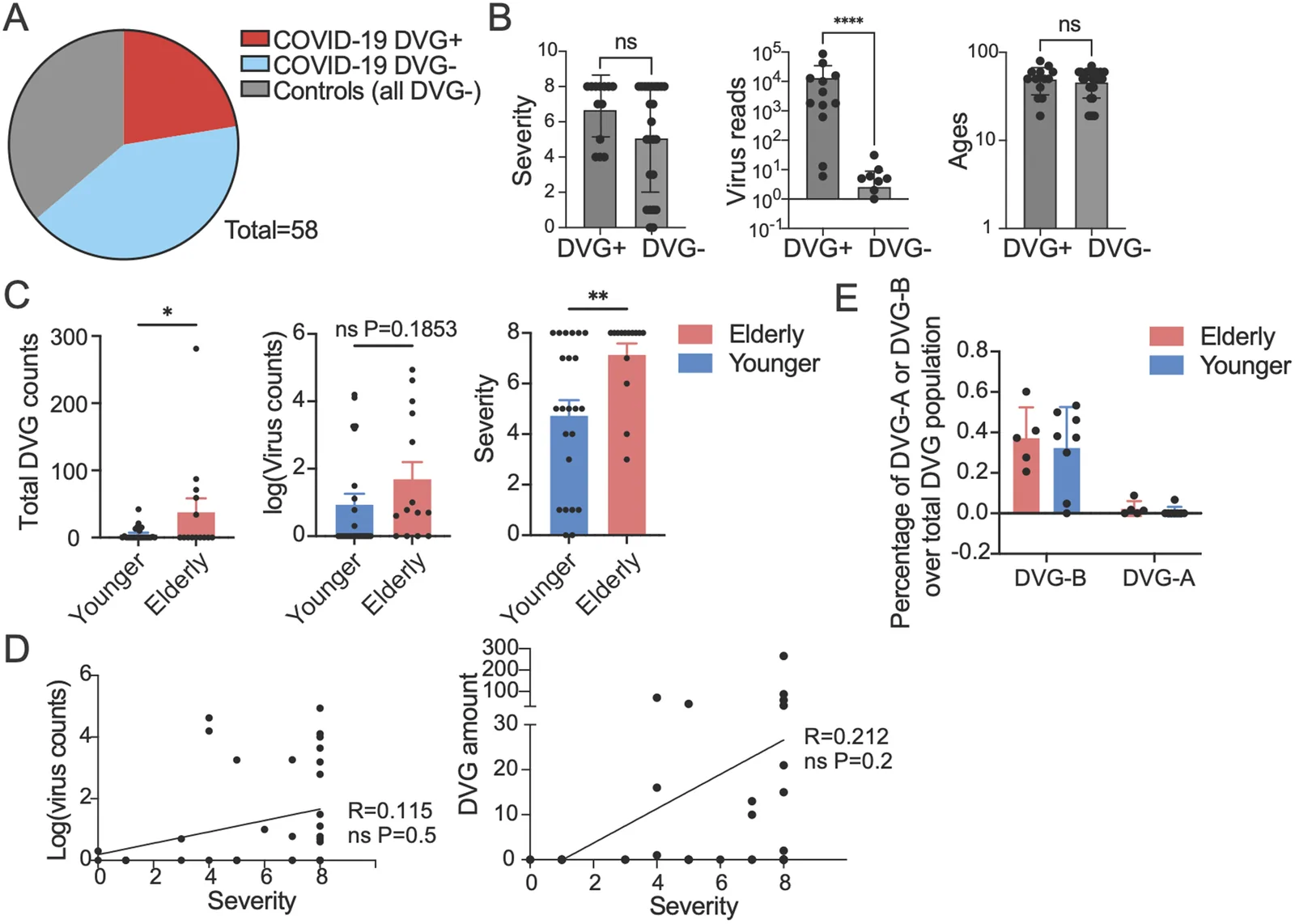
SARS-CoV-2 DVGs positively correlated with COVID-19 disease severity. (A) The pie chart shows the proportions of DVG- and DVG+ COVID-19 patients, along with control patients, in the cohort. (B) Bar graphs show the comparisons of disease severity score, virus reads, and ages between DVG+ and DVG- COVID-19 patients. (C) Bar graphs comparing the total DVG read counts, viral counts, and severity score between younger (<65 yrs old) and elderly (>65 yrs old) COVID-19 patients. * P < 0.05, ** P < 0.01, **** P < 0.0001 by Mann-Whitney test. Mean±SD (D) Shows the correlation between total viral read counts or DVG read counts and disease severity in COVID-19 patients. * P < 0.05 by Pearson r correlation. (E) The bar graph displays the percentages of DVGs in hotspots A and B among DVG+ COVID-19 patients from two different age groups.

### DVGs clustered in hotspot B, but not hotspot A, were associated with the induction of type I and type III IFNs

As a population, SARS-CoV-2 DVGs have been associated with enhanced type I and type III IFN production during *in vitro* infection [17]. To examine whether DVGs in different hotspots equally contribute to IFN responses, we analyzed one publicly available scRNA-seq dataset using primary adult human bronchial epithelial cells infected at an MOI of 0.01 (GSE166766 [31]). From this dataset we previously found that DVGs were majorly detected at days 2 and 3 post infection [17]. Focusing on 2 days post-infection (2 dpi), we examined DVG+ cells according to their DVG junction positions (E’ and V’ in Fig 2A) and assessed their association with single-cell expression levels of IFNB1, IL-29 (IFNL1), and Mx1. Surprisingly, we found that IFN-high-expressing cells (red dots) were clustered in DVG hotspot B but completely absent in hotspot A (Fig 2B). The same trend was observed in the three cell types containing the greatest amount of DVGs (Fig 2C). Although there were more cells positive for hotspot B than hotspot A, the average viral counts and DVG counts per cell remained the same between the two (Fig 2D). A similar trend was observed for samples at 3 dpi (S1 Fig.). Together, these results strongly suggest that DVGs in hotspot B, but not hotspot A, contribute to the induction of IFN responses during infection.

**Fig 2:**
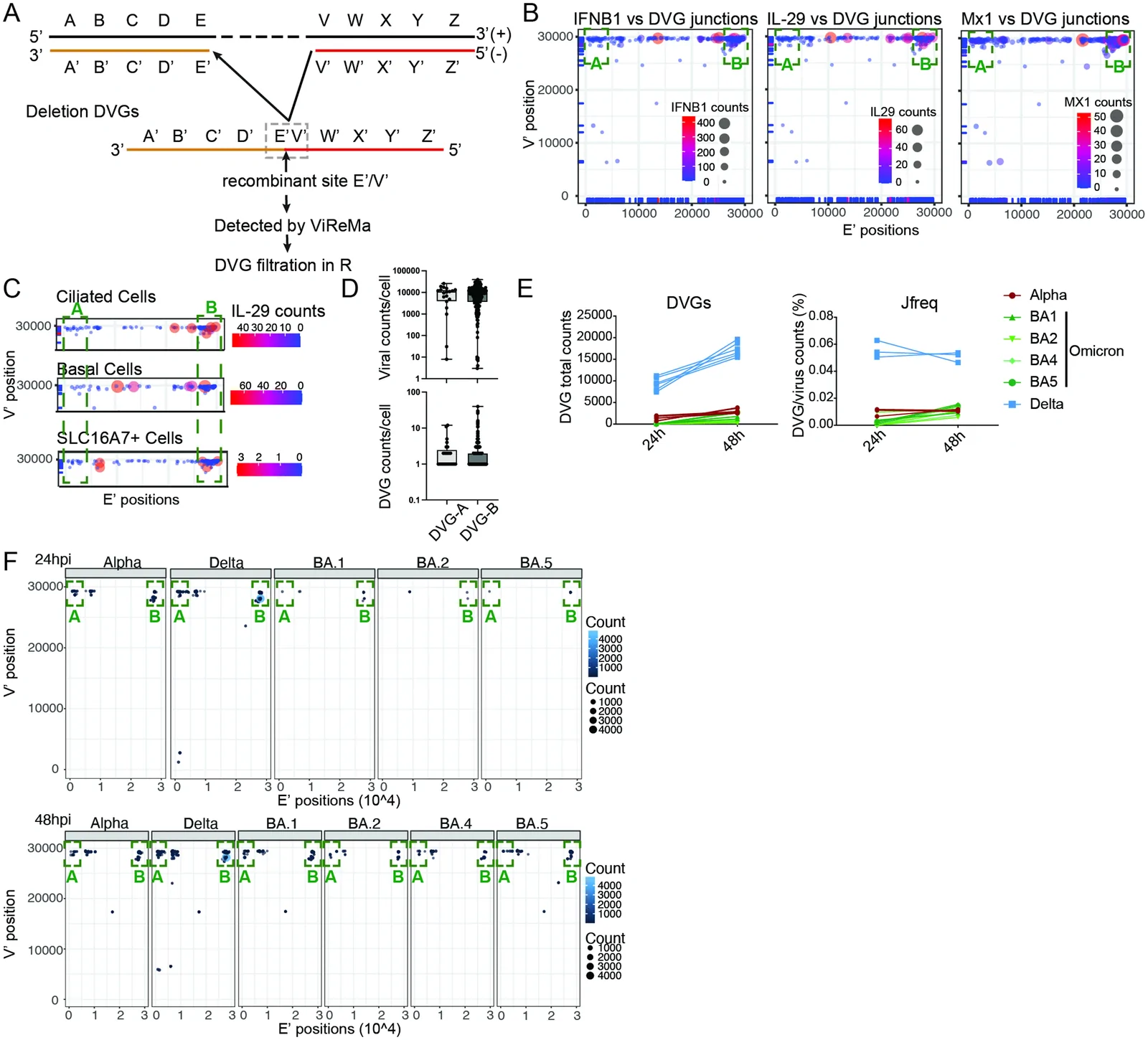
DVGs clustered in hotspot A and B showed different capacities to induce IFN responses via single-cell RNA-seq. (A) Schematic representation of DVG generation from the positive sense viral genome and the general principle of ViReMa identification of deletion DVGs. The V’ site represents the break point and the E’ site represents the rejoin point of the viral polymerase in the formation of DVGs. The gray dashed box marks the recombination site that distinguishes DVGs from full-length viral genomes, which are identified by ViReMa, and further filtered using a previously published R script. (B) Gene expression of IFNB1, IL-29, and Mx1 was correlated with DVG positions at a single cell level, 2 dpi in NHBE cells (GSE166766). Each dot represents an individual DVG+ cell. Both the color and size of dots were based on the expression level of indicated genes. Green brackets indicate the previously identified hotspots A and B. (C) Within the three major cell types positive for DVGs, gene expression of IL-29 (2 dpi) was correlated with DVG positions at the single cell level similarly as shown in (B). (D) At 2 dpi, the viral reads and DVG junction reads in cells positive for DVGs at hotspot A (DVG-A) were compared with those in cells positive for DVGs at hotspot B (DVG-B). DVG junction hotspots among different SARS-CoV-2 variants were then analyzed using the publicly available dataset GSE213759. (E) The total DVG junction read counts, and J_freq_ percentages were graphed for different variants of concern: Alpha, Delta and several Omicrons at 24 and 48 hpi in Calu3 cells. (F) Shows break point (V’) and rejoin point (E’) distributions for DVGs from (E) with the upper and lower panels indicating 24- and 48 hpi, respectively. Circle size and color intensity indicate DVG counts. Green brackets indicate the previously identified hotspot A and B.

### DVG junction hotspots were conserved among different SARS-CoV-2 variants

Because we observed different IFN induction capacities by DVGs from hotspot A and hotspot B, we asked whether these two hotspots are conserved among different SARS-CoV-2 variants. To do this, we obtained another publicly available NGS dataset (GSE213759), wherein Calu3 cells were infected with different variants of concern (VOC) at the same MOI [32]. We specifically analyzed DVG amounts and their genomic locations at 24- and 48-hours post infection (hpi). We detected greater total DVG amounts and Jfreq (DVG reads normalized to total viral reads) in the Delta variant compared with other variants (Fig 2E). This higher level may reflect more efficient production and/or enrichment of DVGs in the Delta variant virus stocks during passaging and virus stock preparation. Importantly, all variants showed DVG hotspot A and B at 24 or 48 hpi (Fig 2F), indicating that these two hotspots were conserved among different variants during *in vitro* infection.

### Synthetic DVG-A and DVG-B particles attenuated WT virus replication, but only DVG-B stimulated robust IFN responses

To directly examine the species-specific function of SARS-CoV-2 DVGs during infection, we synthesized SARS-CoV-2 virus-like particles (VLPs) specifically packaging a representative DVG from hotspot A (DVG-A) and hotspot B (DVG-B), respectively. To capture a broad diversity of DVG recombination junctions and the cumulative functional consequences of deletions arising within these hotspots, we selected the outer boundaries of these respective recombination junctions to construct DVG-A and DVG-B. Based on the outermost range of hotspot A recombination junctions, DVG-A harbors a deletion ablating genomic positions 700–29,674. Therefore, we constructed a DVG-A plasmid by replacing this deletion with the mKate2 gene and an identified virus packaging signal located in nsp15 and nsp16 (PS9) [33] (Fig 3A, right scheme). We then co-transfected the DVG-A plasmid with plasmids encoding the four SARS-CoV-2 structural proteins (E, M, S, and N) in HEK293T cells as previously reported [33], followed by purification using a 30% sucrose cushion. DVG-A titers were determined by RT-qPCR (Fig 3A, left). The representative DVG-B harbors a deletion spanning genomic positions 27,700–29,674. This deletion region encodes the orf7a/7b, orf8 and N genes. Among these genes, only N is essential, required for virus particle assembly. This deletion was introduced by replacing these viral sequences with the eGFP gene (under the N TRS-B sequence) in the corresponding circular polymerase extension reaction (CPER) fragment and the full-length DVG-B was constructed via the CPER approach [34] (Fig 3A, right scheme). We then co-transfected HEK293T cells with the DVG-B CPER products and plasmids encoding the viral structural proteins. Supernatants were filtered, concentrated, and subsequently passaged three times in VeroE6 cells constitutively expressing the N protein (VeroE6:N), followed by sucrose purification and titration via GFP FFU/ml determination in VeroE6:N cells (Fig 3A, left). To characterize DVG-A, we infected VeroAT cells and BSRT7 cells, which lack ACE2, with either the DVG-A stock (DVG-A+) or the supernatants from HEK293T cells transfected with the DVG-A encoding plasmid alone, without the essential structural proteins (DVG-A-). RT-qPCR was performed on infected cells at 24 hpi. DVG-A genomes were detected only in DVG-A+ infected VeroAT cells, indicating that DVG-A genomes were successfully packaged into SARS-CoV-2 like viral particles (Fig 3B). To characterize DVG-B, VeroAT cells were infected with supernatants from HEK293T cells co-transfected with the DVG-B CPER products and various combinations of the four structural protein encoding plasmids. Expression of all four structural proteins yielded the highest percentage of GFP+ cells, followed by the condition lacking spike. In contrast, transfection with E, M, and S but without N produced no GFP signal, given that DVG-B does not encode N and is therefore unable to assemble virus particles (Fig 3C). To further confirm that DVG-B particles cannot produce infectious virions in cells without N expression, VeroAT cells were infected with DVG-B, and the resulting supernatants were then used to infect new VeroAT cells. As expected, no GFP+ cells were detected by flow cytometry (Fig 3D, left). In contrast, serial passaging of DVG-B in VeroE6:N resulted in GFP+ cells (Fig 3D, right). To assess the expression of other viral proteins encoded by DVG-B, A549-ACE2 cells were infected with WT virus (Hong Kong strain, MOI = 1), DVG-B (MOI = 1), or DVG-A (MOI = 1). By Western blot, the spike protein was readily detected in DVG-B-infected cells, but not DVG-A-infected cells (Fig 3E). Spike protein levels in DVG-B–infected cells were markedly lower than in WT virus–infected cells (Fig 3E).

**Fig 3:**
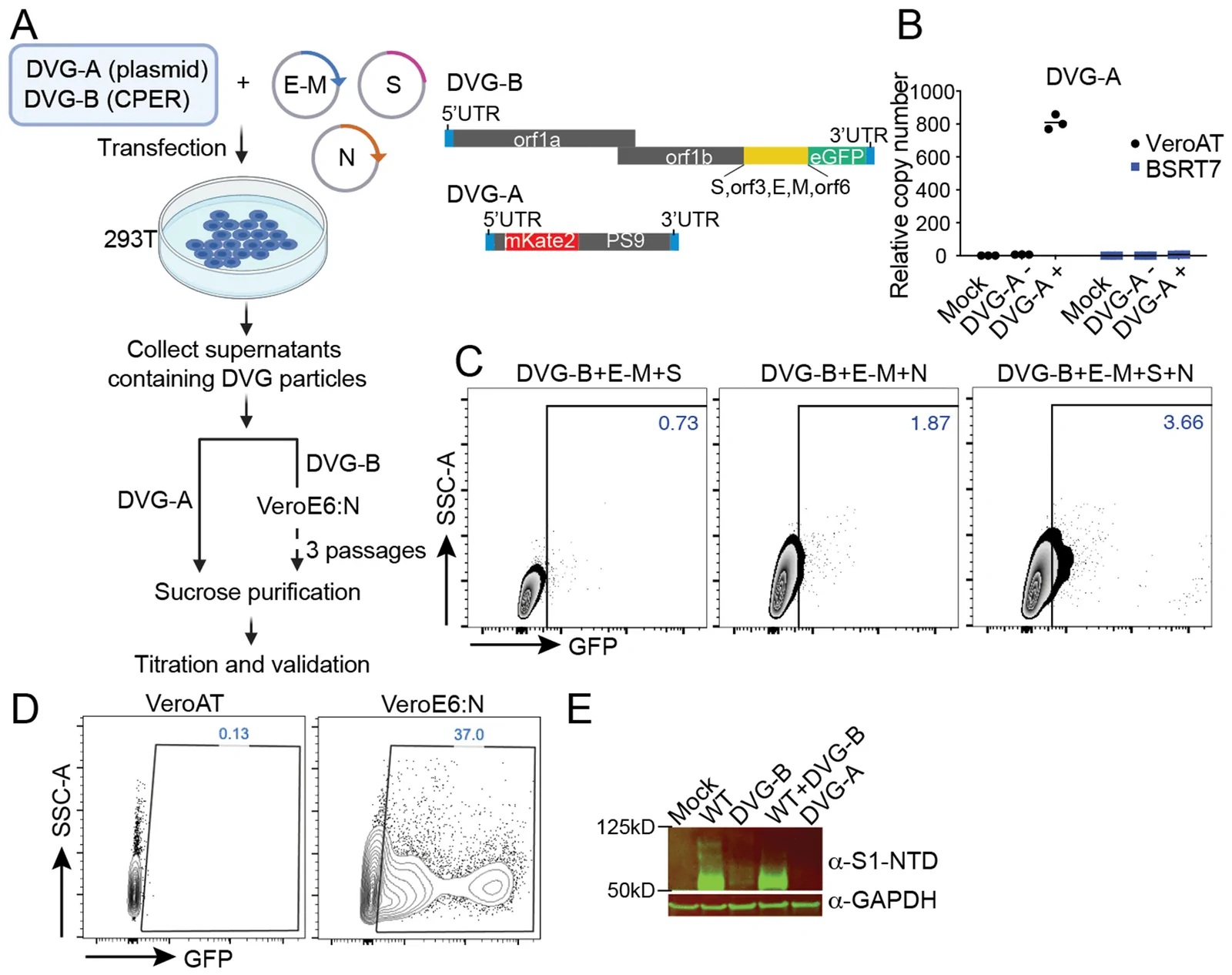
Generation and characterization of representative DVG particles from hotspot A (DVG-A) and hotspot B (DVG-B). (A) Schematic representation of the genomic architectures and production of DVG-A and DVG-B. Briefly, for DVG-B, genomic positions 27,700-29,674 were replaced with eGFP and rejoined to the remaining viral genome using CPER. For DVG-A, genomic positions 700-29,674 were replaced with the mKate2 reporter and PS9 and cloned into a plasmid under the CMV promoter. The DVG-B CPER product or the DVG-A plasmid was co-transfected with plasmids encoding the four viral structural proteins E, M, S, and N into HEK293T cells and DVG-particle-containing supernatants were collected. DVG-A particles were purified and concentrated by 30% sucrose cushion. DVG-B particles were further passaged in VeroE6:N cells for 3 passages to increase titer, followed by sucrose purification. Schematic created in BioRender. SUN, Y. (2026) https://BioRender.com/913okh4 is licensed under CC BY 4.0. (B) VeroAT and BSRT7 cells (ACE2-negative control) were infected with: mock, supernatants from HEK293T cells transfected with the DVG-A plasmid without the structural proteins (DVG-A-), or the DVG-A stock (DVG-A+). RNA was extracted at 24 hpi followed by RT-qPCR targeting DVG-A. (C) HEK293T cells were transfected with DVG-B CPER products and different combinations of E, M, S, and N encoding plasmids. Supernatants were collected and used to infect VeroAT cells. Representative flow plots show GFP + % VeroAT cells infected with indicated supernatants. (D) DVG-B was used to infect VeroAT or VeroE6:N cells. The subsequent supernatants were used to infect VeroAT or VeroE6:N cells. Infected cells were analyzed by flow cytometry to determine the percentage of GFP+ cells at 3 dpi. (E) A549-ACE2 cells were infected with WT SARS-CoV-2 (MOI = 1), DVG-B (MOI = 1), DVG-B + WT (ratio 1:1), and DVG-A (MOI = 1). Cells were collected at 24 hpi, followed by Western blotting using antibodies targeting the S1-NTD of the spike protein. The corresponding original, uncropped, Western blots are shown in S6A Fig.

We next characterized DVG-A and DVG-B (passage 3, P3) stocks to assess their direct effects on WT virus infection and IFN responses. A549-ACE2 cells were infected with WT virus (MOI = 1), DVG-A (MOI = 1), DVG-B (MOI = 1), WT + DVG-A (1:1), or WT + DVG-B (1:1). Cells were collected at 24 hpi for RT-qPCR analysis of IFN-related and viral gene expression. WT virus infection induced minimal IFN responses compared to mock infection. Despite reduced replication, DVG-B infection alone elicited significantly higher IFN responses than WT virus infection (Fig 4A). IFNB1 ELISA of A549-ACE2 supernatants confirmed the production of IFNB1 by DVG-B infection alone (Fig 4C). In contrast, DVG-A did not induce IFN responses during infection (Fig 4B). TCID₅₀ assays showed that both DVG-A and DVG-B suppressed WT virus replication in a dose-dependent manner (Fig 4D-4F). The suppressive effects plateaued at DVG:WT ratios of 5:1 for both DVG-A and DVG-B (Fig 4E–4F).

**Fig 4:**
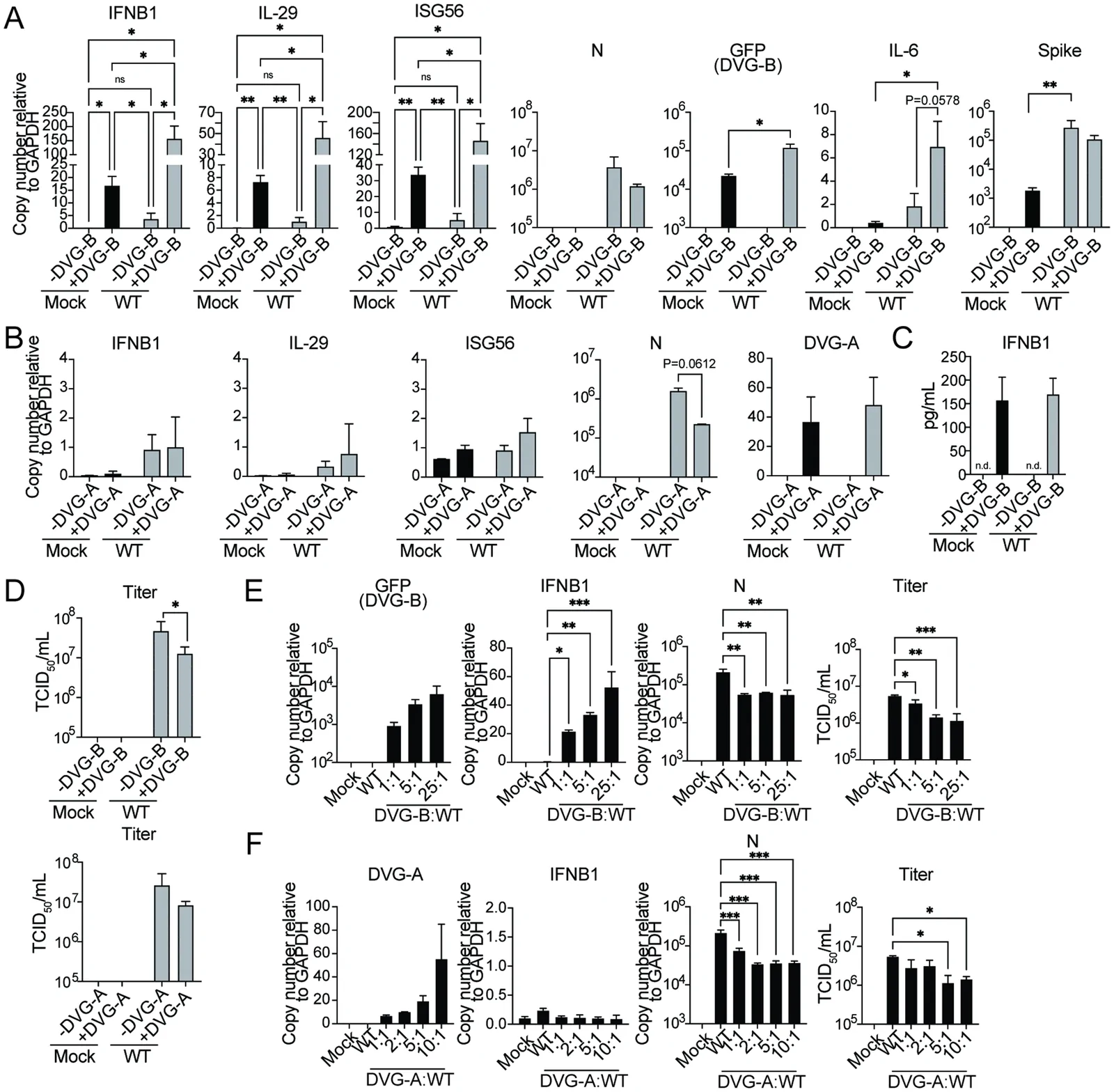
DVG-A and DVG-B containing VLPs antagonized WT SARS-CoV-2 infection. (A-B) A549-ACE2 cells were infected with SARS-CoV-2 (MOI 1), DVG-B (A, MOI 1), DVG-A (B, MOI 1) or co-infected with either DVG-B:SARS-CoV-2 (MOI 1: MOI 1) or DVG-A: SARS-CoV-2 (MOI 1: MOI 1) for 24 hours post-inoculation. Expression of host genes and viral genes/specific DVGs were calculated relative to GAPDH by RT-qPCR. *p < 0.05, **p < 0.01 by two-way ANOVA with Holm-Sidak’s multiple comparisons test. N = 4, mean±SD. (C) Supernatants from mock, DVG-B, SARS-CoV-2 and DVG-B/SARS-CoV-2 co-infected cells were collected and secreted IFNB1 levels were measured by ELISA. N = 3, mean±SD. (D) Progeny infectious virus production was determined from supernatants of SARS-CoV-2, DVG-B/SARS-CoV-2 and DVG-A/SARS-CoV-2 co-infected cells **(**A-B**)** by TCID_50_/mL. Viral titration was performed in VeroAT cells. *p < 0.05 by paired one-tailed t-test. N = 4, mean±SD. (E-F) Cells were infected with SARS-CoV-2 (MOI 0.1) with increasing ratios of DVG-B: SARS-CoV-2 (E, MOI 0.1, 0.5, 2.5: MOI 0.1) or DVG-A: SARS-CoV-2 (F, MOI 0.1, 0.2, 0.5, 1: MOI 0.1). IFNB1, N and specific DVG copy numbers were quantified relative to GAPDH by RT-qPCR. SARS-CoV-2 titers, with increasing input MOIs of DVG-B or DVG-A, were measured by TCID_50_/mL. *p < 0.05, **p < 0.01, ***p < 0.001 by one-way ANOVA with Dunnett’s multiple comparisons test. N = 2, mean±SD.

To further compare the impact of DVG and WT virus infections on the cellular transcriptome, bulk RNA-seq was performed on infected cells from Fig 4A-4B. Principal component analysis (PCA) separated DVG-B infection from mock, DVG-A, and WT virus infections, while DVG-A–infected cells exhibited a gene expression profile more similar to the mock controls (S2A Fig). First, comparison of DVG-B and WT virus single- and co-infections identified 1877 differentially expressed genes (DEGs). These genes were grouped into 4 clusters by heatmap (Fig 5A). The majority of DEGs were upregulated during WT virus infection (cluster 1), followed by upregulation during DVG-B infection (cluster 2). Gene ontology (GO) analysis revealed that cluster 1 genes were primarily enriched in TNFa-NFkB signaling, MAPK cascade, and IL-1 production pathways, all of which are related to inflammation (Fig 5B). Surprisingly, only 107 genes were upregulated in DVG-B–infected cells compared to mock infected controls. Consistent with our RT-qPCR results, antiviral response pathways were the most significantly enriched among genes upregulated in DVG-B-infected cells (Fig 5C). Very few genes were significantly downregulated in WT- and DVG-B-infected cells, and no pathways showed significant enrichment. We next performed similar comparisons to DVG-A vs. WT virus. Likewise, the majority of DEGs were associated with WT virus infection, and gene expression in co-infected cells was markedly reduced compared with WT virus infection alone, indicating that DVG-A significantly suppressed WT virus infection (S2B Fig). Consistent with the PCA results, very few genes were differentially expressed specifically in DVG-A–infected cells compared to mock. Taken together, our data directly demonstrate that both DVG-A and DVG-B suppressed WT virus replication. While the WT virus profoundly altered the host transcriptome, DVG-B, but not DVG-A, selectively upregulated type I and type III antiviral responses.

**Fig 5:**
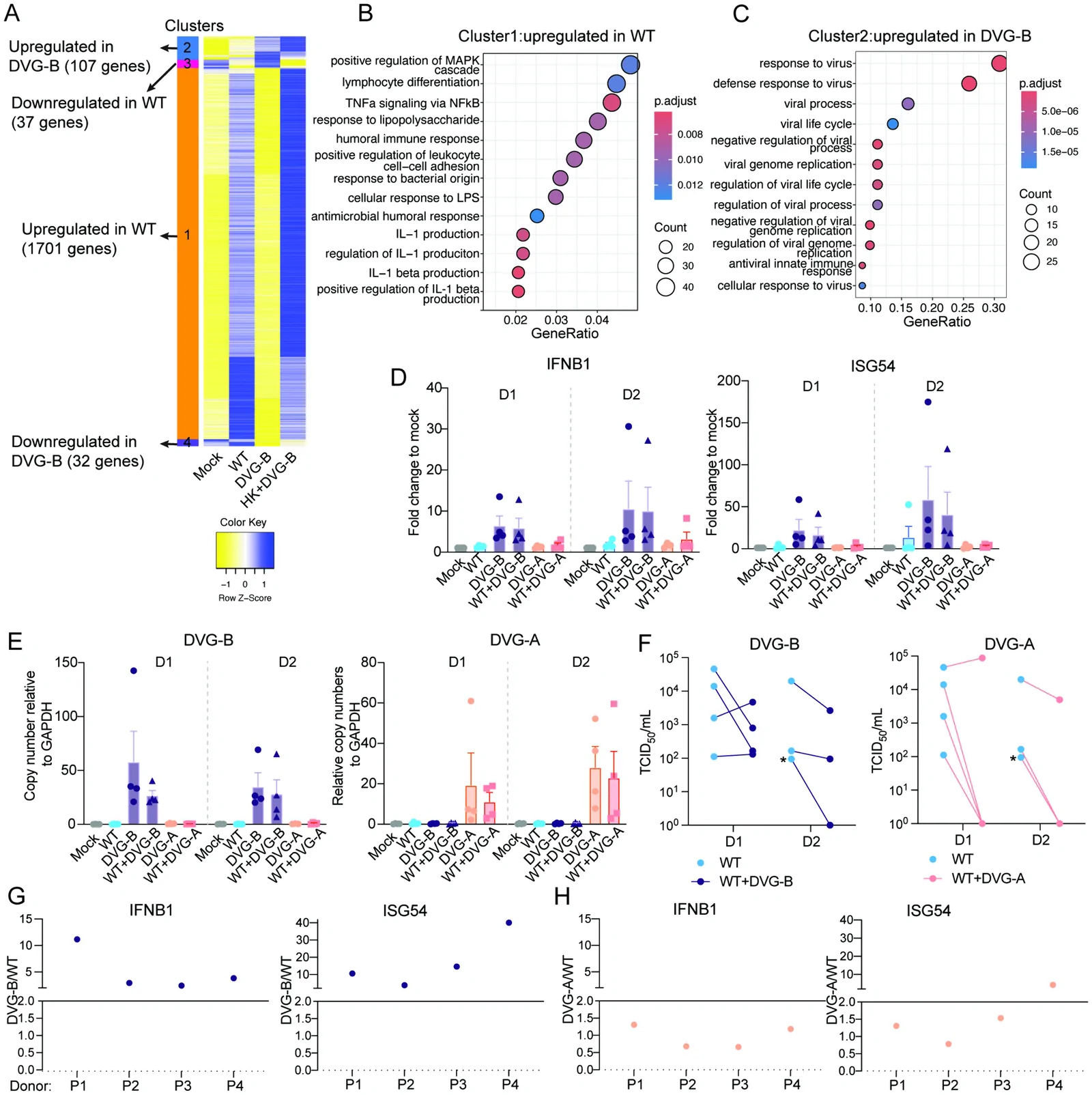
Transcriptomic profiling of SARS-CoV-2– and DVG-infected cells, and the effects of DVG-A and DVG-B infection in PCLS. (A-C) Total RNA from infected A549-ACE2 cells (Fig 4A-4B) was subjected to bulk RNA sequencing. Differentially expressed genes (DEGs, ≥ two-fold and ≤ 1% false discovery rate) were identified by comparing DVG-B–infected cells with mock-infected and SARS-CoV-2–infected cells. The heatmap (A) shows DEGs grouped into four clusters. Enriched pathways for cluster 1 and cluster 2 are shown in (B) and (C), respectively. Detailed DEG lists for each cluster are shown in S5-S8 Tables. (D-E) PCLS were either singly infected or co-infected with WT virus and DVG-A or DVG-B. Co-infections were performed using a WT:DVG-B ratio of 1:5 and a WT:DVG-A ratio of 1:20. Supernatants and tissue slices were harvested at 24 and 48 hpi. The expression of IFNB1 (D), ISG54 (D), DVG-B (E), and DVG-A (E) were examined in PCLS via RT-qPCR. N = 4, mean±SD. (F) Infectious virus titers from PCLS supernatants via TCID_50_/mL. * Indicates that there were two PCLS donors with the same output viral titers. (G-H) Ratio of gene expression in DVG-B (G) or DVG-A (H) and WT virus infected tissue from all 4 donors (P1-P4). Ratios >1 indicate that DVGs induced higher gene expression than WT virus. Each dot represents one individual donor.

### DVG-B particles stimulated IFN responses in ex vivo human PCLS

To further assess whether DVG-B induces IFN responses in a physiologically relevant system, human PCLS from four adult donors were infected with DVGs and WT virus. Previous studies have shown that WT virus titers in PCLS peaks between 1 and 2 dpi [35]; therefore, these time points were selected for analysis of IFN responses and infectious virus titers. We observed that DVG-B infection consistently elicited higher type I IFN responses compared with mock controls at both 1 and 2 dpi, whereas this trend was not observed following DVG-A infection (Fig 5D and 5E). Co-infection with the WT virus and either DVG-A or DVG-B resulted in reduced viral titers in all four donors at 2 dpi (Fig 5F), further supporting the suppressive effects of both DVGs on WT virus replication. Interestingly, despite donor variability in responsiveness, the ratios of DVG-B to WT virus for IFNB1 expression (4/4 donors) and ISG54 expression (3/4 donors) were higher than those observed for DVG-A relative to the WT virus (Fig 5G vs 5H), further indicating that DVG-B, but not DVG-A, robustly induced type I IFN responses *ex vivo*.

### DVG-B-derived dsRNA exhibited a distinct subcellular distribution and was associated with IRF3 nuclear translocation

Double-stranded RNA (dsRNA) is the genomic replication intermediate during CoV infection and can be recognized by MDA5 or RIG-I to trigger IFN responses [21,22]. However, dsRNA is normally sequestered within DMVs, also known as replication organelles (RO), which limits their detection via RLR pathways. We first examined the amount and cellular distribution of DVG-B-derived dsRNA by immunofluorescence microscopy and found that the total dsRNA levels were significantly lower during DVG-B infection compared to WT (Fig 6A; quantification in 6B), indicating reduced DVG-B replication levels. This is consistent with the markedly lower spike expression observed in DVG-B-infected cells compared with WT virus (Figs 3E and 6C). Interestingly, we found that while dsRNA in WT infected cells concentrated at the perinuclear region, DVG-B-derived dsRNA was more diffusely distributed throughout the cell. This distinct distribution became apparent at 12 hpi and was prominent by 24 hpi (Fig 6A), coinciding with the time point when DVG-B infection significantly induced IFNB1 expression (Fig 6C). We next co-stained DVG-B and WT-infected cells for IRF3 and dsRNA at 24 hpi. Consistently, despite fewer dsRNA-positive cells, DVG-B infection resulted in a higher proportion of dsRNA+ cells exhibiting nuclear IRF3 than WT virus infection, suggesting that DVG-B-derived dsRNA/RNA activated the RLR pathway more efficiently than WT (white arrows in Fig 6D left panel, quantification in Fig 6E). Because dsRNA formation requires viral genomic replication, dsRNA should not be detected when viral replication is inhibited. Therefore, we treated A549-ACE2 cells with remdesivir, an inhibitor of the SARS-CoV-2 RNA-dependent RNA-polymerase, before and during infection with DVG-B or WT virus. In the presence of remdesivir, no dsRNA was detected and IRF-3 remained majorly cytoplasmic at 24 hpi (Fig 6D, right panel). Correspondingly, DVG-B-induced IFN production was abolished (Fig 6F). To verify that remdesivir does not suppress virus entry, we infected remdesivir-treated cells with DVG-A particles. Because DVG-A cannot replicate its genome independently, the amount of DVG-A detected from infected cells represents the number of DVG-A particles that have entered the cells. As expected, remdesivir did not reduce DVG-A levels (Fig 6G). To confirm that DVG-B infection induced the RLR pathway, we infected MAVS knockout (KO) and Protein Kinase R (PKR) KO A549-ACE2 cells with DVG-B. As expected, MAVS KO, but not PKR KO, ablated IFN induction during DVG-B infection (Fig 6H). To determine which RLR sensor is responsible for recognizing DVG-B-derived dsRNA, we transfected A549-ACE2 cells with siRNAs targeting either RIG-I or MDA5, followed by DVG-B infection. Interestingly, although both sensors contributed to the antiviral response, RIG-I knockdown markedly reduced DVG-B-induced IFNB1 expression (Fig 6I–J), demonstrating that RIG-I is the primary sensor detecting DVG-B-derived dsRNA. Notably, MDA5 mRNA levels were also reduced in RIG-I-knockdown cells following DVG-B infection, likely as a secondary consequence of the marked reduction in IFNB1 induction compared with scrambled siRNA-treated cells. Additionally, under mock-infected conditions, only the targeted gene was reduced, indicating that the siRNA knockdown was specific (Fig 6I). Taken together, our data indicate that the replication products of DVG-B, likely dsRNA, activated the RLR pathway to stimulate strong IFN responses.

**Fig 6:**
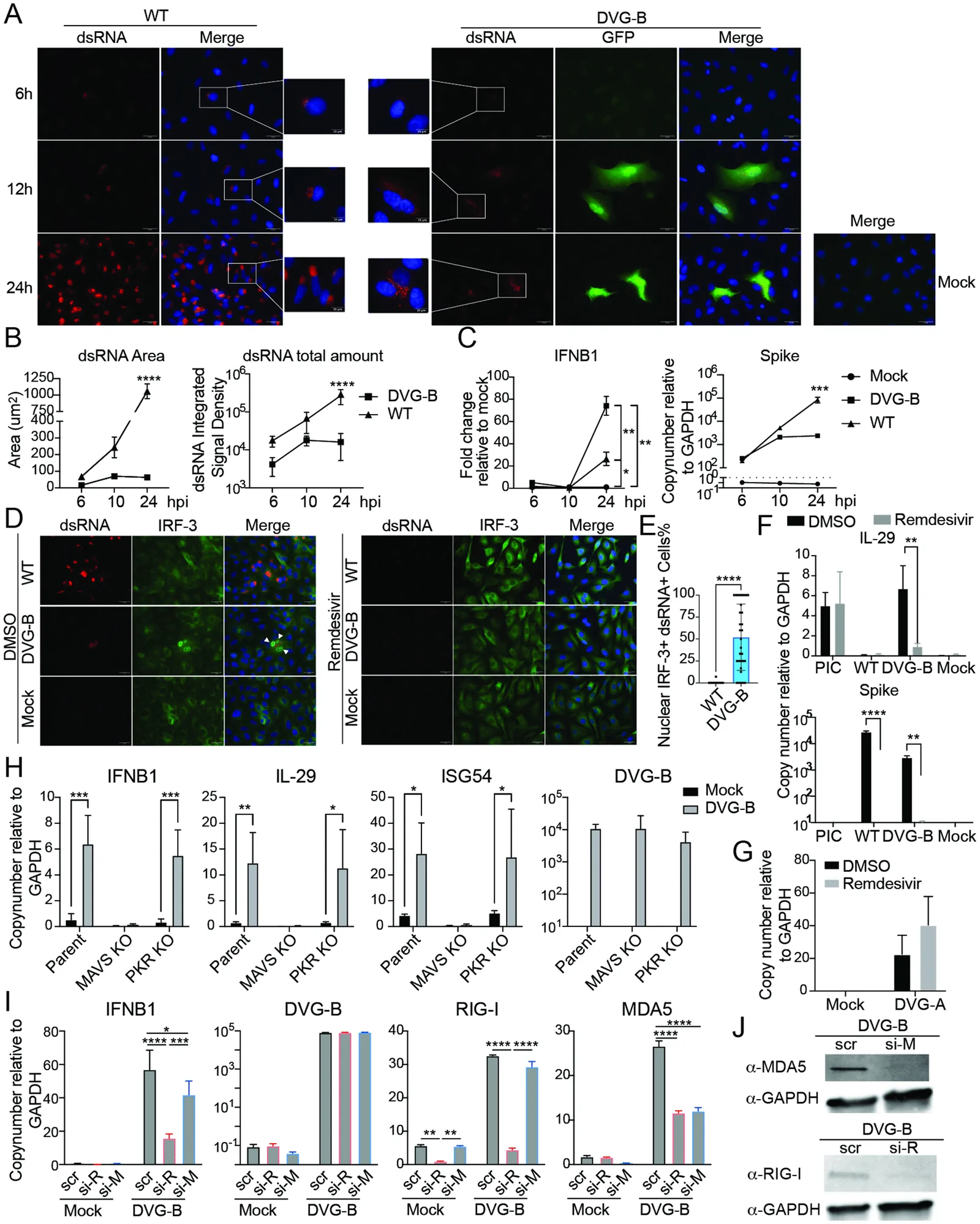
DVG-B-derived dsRNA localized to discrete puncta and induced significantly greater IRF-3 nuclear translocation compared to SARS-CoV-2. (A-C) A549-ACE2 cells were infected with SARS-CoV-2 or DVG-B (both MOI 1) and cells were collected at 6-, 10- and 24-hours post-inoculation for epifluorescence microscopy to (A) compare the cellular distribution of SARS-CoV-2 and DVG-B-derived dsRNA and (B) quantify the total area of dsRNA signal and total dsRNA signal intensity (integrated signal density). Insets depict magnified images of cells indicated by the white boxes. Scale bars indicate 10μm and 25μm, respectively. ****p < 0.0001 by two-way ANOVA with Bonferroni’s multiple comparisons test. 6 hpi N = 4 fields. 10 hpi N = 5 fields. 24 hpi, SARS-CoV-2 N = 16 fields, DVG-B N = 34 fields. Mean±SD. (C) IFNB1 and spike gene expression were calculated relative to GAPDH by RT-qPCR. IFNB1 copy number values were normalized to mock for each respective timepoint. *p < 0.05, **p < 0.01, ***p < 0.001 by two-way ANOVA with Tukey’s multiple comparisons test. N = 3, mean±SD. (D-E) A549-ACE2 cells treated with 10 μM remdesivir or an equal volume of DMSO were infected with SARS-CoV-2 or DVG-B (MOI 1) for 24-hours post-inoculation, respectively. N = 2 biological repeats (D) Epifluorescence images of dsRNA and IRF-3 staining in DMSO (left) and remdesivir (right) treated cells. Scale bars indicate 25 μm. Arrowheads indicate dsRNA+ cells with nuclear IRF-3. Remdesivir treated SARS-CoV-2 and DVG-B infections N = 3 fields, respectively. DMSO treated SARS-CoV-2 infections N = 14 fields, DVG-B infections N = 30 fields. (E) Quantification of the proportion of dsRNA+ cells with nuclear IRF-3 relative to the total number of dsRNA+ cells per field. Each point represents one field. ****p < 0.0001 by unpaired two-tailed t-test. SARS-CoV-2: 412 dsRNA+ cells among N = 14 fields. DVG-B: 120 dsRNA+ cells among N = 30 fields. (F) A549-ACE2 cells treated with 10 μM remdesivir or an equal volume of DMSO were infected with SARS-CoV-2 or DVG-B (MOI 1) or were transfected with low molecular weight poly(I:C) (PIC) for 24-hours post-inoculation, respectively. IL-29 and spike gene copy numbers were calculated relative to GAPDH by RT-qPCR. **p < 0.01, ****p < 0.0001 by mixed-effects analysis with Bonferroni’s multiple comparisons test. N = 3, mean±SD. (G) A549-ACE2 cells were treated with 10 μM remdesivir or an equal volume of DMSO and infected with DVG-A (MOI 1) for 24-hours post-inoculation. DVG-A levels were quantified relative to GAPDH by RT-qPCR. N = 4, mean±SD. (H) A549-ACE2 MAVS KO, PKR KO, or empty vector transduced control cells (parent) were infected with DVG-B (MOI 1) for 24-hours post-inoculation. IFNB1, IL-29, ISG54 and DVG-B copy numbers were quantified relative to GAPDH by RT-qPCR. *p < 0.05, **p < 0.01, ***p < 0.001 by two-way ANOVA with Bonferroni’s multiple comparisons test. N = 4, mean±SD. (I-J) A549-ACE2 cells were transfected with scramble siRNA (scr) and siRNAs targeting RIG-I (si-R) and MDA5 (si-M) for 24 hours, followed by DVG-B infection (MOI 1). (I) Total RNA was extracted 24 hpi. IFNB1, GFP (DVG-B), RIG-I, and MDA5 copy numbers were quantified relative to GAPDH by RT-qPCR. *p < 0.05, **p < 0.01, ***p < 0.001, ****p < 0.0001 by two-way ANOVA with Sidak’s multiple comparisons test. N = 2-3, mean±SD. (J) DVG-B-infected cells treated with indicated siRNAs were collected at 24 hpi, followed by Western blotting using antibodies targeting RIG-I, MDA5, and GAPDH. The corresponding original, uncropped Western blots are shown in S6B-S6C Fig.

To examine whether there were additional mutations and deletions in our DVG-B stocks, we performed RNA deep sequencing on passage 2 (P2) and passage 3 (P3) DVG-B stocks, aligned the RNA-seq reads to either the WT virus or DVG-B reference genomes, and visualized the aligned files with IGV (Integrative Genomics Viewer) [36]. The introduced deletion at 27,700–29,674 was observed when aligned to the WT virus reference genome, whereas this deletion was not observed in the alignment using DVG-B as the reference genome (S3A-B Fig.). Additional mutations were identified that were not present in the published sequences of the CPER constructs (S2 Table in Supporting Information). Among them, 9 substitution mutations (7 nonsynonymous) were considered major, given that these mutations comprised more than 50% of the ribonucleotides at those positions (* in S2 Table in Supporting Information). Among the 7 nonsynonymous substitutions, one occurred in nsp1 (no known function), one occurred in spike (adaptive mutation at S1/S2 cleavage site to enhance entry in Vero cells [37]), and the remaining 5 mutations were located in nsp12 (no known function). Based on the nsp12 crystal structure, the seven substitutions were clustered adjacent to nsp12 catalytic motif A [38]. Therefore, we introduced these mutations in nsp12 and further examined their effects on viral polymerase activity via a mini-replicon system [39]. This system is comprised of three helper plasmids expressing all 16 nonstructural proteins to form the SARS-CoV-2 viral polymerase complex (Vp), along with a backbone (BKB) plasmid containing the 5′UTR, a firefly luciferase reporter driven by the M gene TRS-B sequence, and the 3′UTR. A previous study demonstrated that firefly luciferase expression occurs only in the presence of the helper plasmids [39]. HEK293T cells were co-transfected with the BKB plasmid, a Renilla luciferase plasmid (transfection control), and either the WT Vp or nsp12 mutant Vp expressing plasmid. No significant difference in the firefly-to-Renilla luciferase activity ratio was observed between the WT and nsp12 mutant Vps (S3F Fig.), indicating that these nsp12 mutations do not substantially affect viral polymerase activity and are therefore unlikely to account for the altered dsRNA distribution.

In addition to point mutations, we identified additional deletions in the DVG-B stocks using the same pipeline [17]. These deletions were present at low levels in the P2 stock and became more enriched by P3 (S3C Fig.). The junctions were primarily located within orf6 and the inserted eGFP gene, near the 3′ end of the DVG-B genome (S3D Fig). To determine whether these deletions contributed to DVG-B’s IFN induction phenotype, we infected A549-ACE2 cells with P2 and P3 DVG-B stocks at MOI 1. Both P2 and P3 DVG-B induced comparable IFN responses (S3E Fig), indicating that these additional deletions did not alter DVG-B’s ability to induce IFN. Therefore, these analyses suggest that the absence of the viral proteins encoded between positions 27,700 and 29,674 (the deletion range of hotspot B) were likely the major determinant of DVG-B’s distinct dsRNA distribution and robust IFN induction.

### Trans-complementation of the SARS-CoV-2 N protein partially restored the DVG-B-derived dsRNA pattern to the WT dsRNA phenotype, but not the IFN phenotype

The SARS-CoV-2 nucleocapsid (N) protein is required for ribonucleocapsid formation and virus particle assembly. Additionally, multiple other key functions have been described for the N protein including undergoing liquid-liquid phase separation, interacting with the DMV via nsp3, and innate immune antagonism [40–43]. Therefore, to determine whether N complementation alters DVG-B dsRNA distribution, VeroAT or VeroE6:N cells were infected with DVG-B for 24 hours. Confocal microscopy showed that DVG-B–derived dsRNA was widely distributed in VeroAT cells, similar to our observations in A549-ACE2 cells (Figs 6A and S4A). In contrast, in VeroE6:N cells, most dsRNA localized to the perinuclear region and was closely associated with N (S4A Fig). It should be noted that although N protein complementation dramatically altered the distribution of DVG-B-derived dsRNA, regions of dsRNA signal with no apparent colocalizing N protein were more abundant than in WT virus infected VeroE6:N cells.

Because Vero cells are deficient in type I IFN responses [44], we generated two A549-ACE2 cell lines constitutively expressing the SARS-CoV-2 N protein. The first expressed a strep-II tagged N-protein (A549:N, S4B Fig) while the second lacked a protein tag (A549:N:NPT). A cell line transduced with an empty lentiviral vector encoding the puromycin resistance gene (A549:EV) was generated as a control. In A549:EV cells, WT-derived dsRNA localized to the perinuclear region and colocalized with N (R = 0.67; Fig 7C, upper panel), whereas DVG-B–derived dsRNA remained dispersed (Fig 7A). N complementation increased dsRNA levels and largely restored a WT-like distribution with N–dsRNA colocalization (Fig 7A, quantification in 7C upper panel). Similar results were observed in A549:N:NPT cells, indicating that the strep-II tag did not affect dsRNA redistribution (S4D Fig). Next, we examined the location of dsRNA in relation to DMVs. We used nsp6 as an approximation for DMV localization given that nsp6 zippered endoplasmic reticulum membranes reportedly connect to DMVs [45]. In WT infection, dsRNA strongly colocalized with nsp6 in the perinuclear region (R = 0.74; Fig 7B, quantification in Fig 7C lower panel). During DVG-B infection in A549:EV cells, the nsp6 signal and its correlation with dsRNA were reduced (R = 0.41). N complementation increased nsp6 abundance and partially restored the dsRNA–nsp6 association (R = 0.53, Fig 7B), though not to WT levels. N also strongly correlated with nsp6 during DVG-B infection in A549:N cells (R = 0.76, S4C Fig). It should be noted that in both A549:N and VeroE6:N cells, the dsRNA and nsp6 patterns did not fully recapitulate the WT phenotype during DVG-B infection. Nonetheless, our confocal data indicate that N complementation largely restored DVG-B-derived dsRNA to the WT virus distribution pattern and suggest that the N protein influences DMV organization or maintenance.

**Fig 7:**
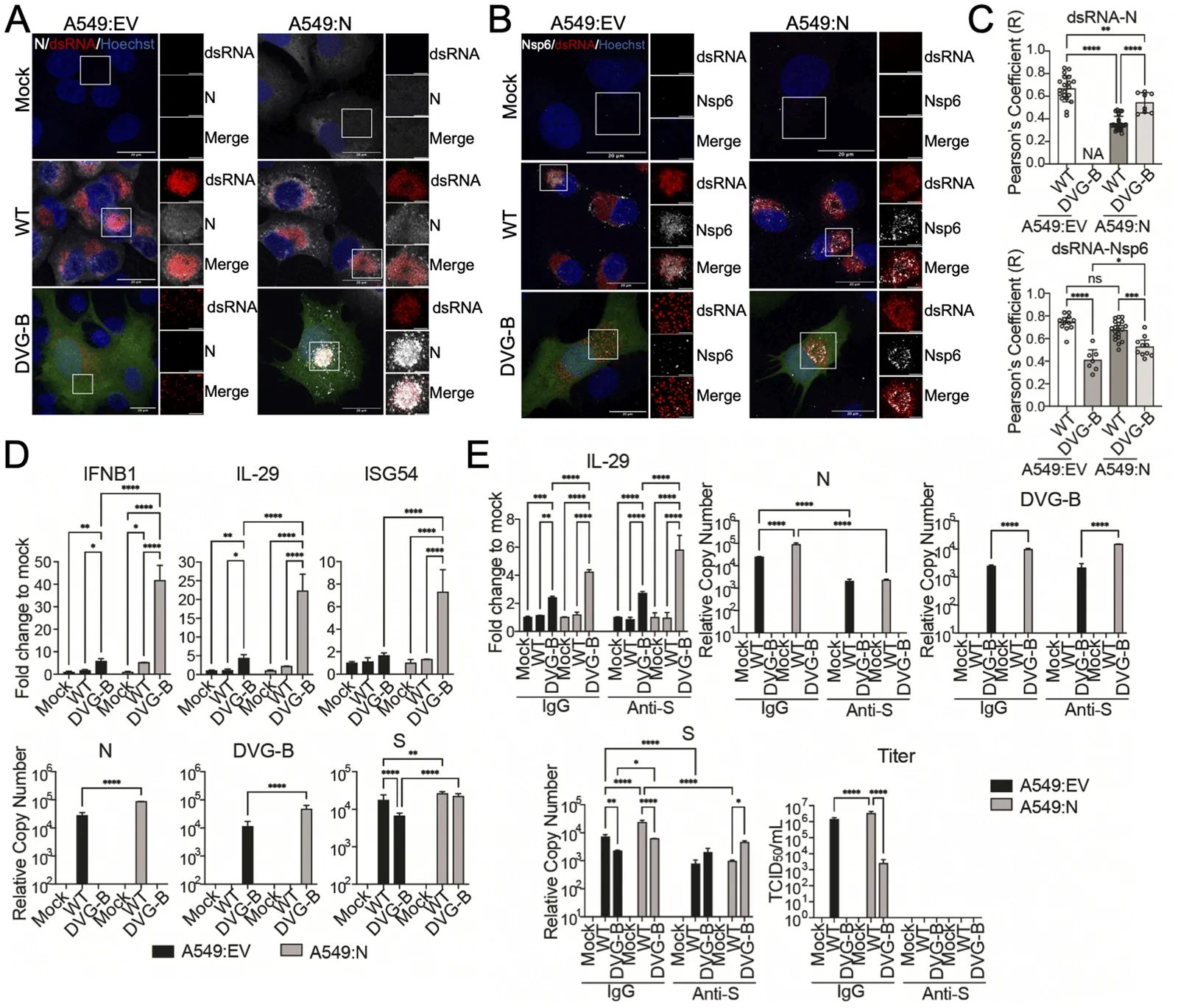
Trans-complementation of the SARS-CoV-2 N protein partially restored the DVG-B-derived dsRNA pattern to the SARS-CoV-2 dsRNA phenotype. (A-B) A549:EV and A549:N cells were infected with WT (MOI 0.1) or DVG-B (MOI 1), respectively, and cells were collected 24 hours post-inoculation for confocal microscopy. Maximum intensity projections of merged dsRNA (A-B, red) staining with the N protein (A, white) or nsp6 (B, white) in A549-EV and A549:N cells. Nuclei were Hoechst stained (blue). Note that DVG-B encoded GFP is depicted (green). Scale bar indicates 20μm. Insets depict magnified cell regions indicated by the white boxes of dsRNA and N protein co-staining (A) or dsRNA and nsp6 co-staining (B). Scale bar indicates 5μm. Graphs indicate the Pearson’s correlation coefficient (*R)* values for the co-localization of dsRNA and N (C**,** above) or dsRNA and nsp6 (C**,** below) in A549:EV and A549:N cells, respectively. Individual values are shown. *p < 0.05, **p < 0.01, ***p < 0.001, ****p < 0.0001 by one-way ANOVA with Tukey’s multiple comparisons test. Mean±SD. For quantification in dsRNA-N co-staining, N = 20 fields of WT in A549:EV, N = 27 fields of WT and N = 8 fields of DVG-B in A549:N. For quantification of dsRNA-nsp6 co-staining, N = 12 fields of WT and N = 7 fields of DVG-B in A549:EV, N = 19 fields of WT and N = 10 fields of DVG-B in A549:N. (D) A549:EV and A549:N cells were infected with WT (MOI 0.1) or DVG-B (MOI 1), respectively, and cells were collected 24-hours post-inoculation for RT-qPCR. Expression of host genes and viral genes/DVG-B were calculated relative to GAPDH. IFNB1, IL-29 and ISG54 relative copy number values were normalized to mock for A549:EV and A549:N, respectively. Because the A549:N cell line expresses codon-optimized N, our qPCR primers specifically detect virus-derived N transcripts. *p < 0.05, **p < 0.01, ***p < 0.001, ****p < 0.0001 by two-way ANOVA with Tukey’s multiple comparisons test. N = 2 biological repeats, mean±SD. (E) A549:EV and A549:N cells were inoculated as in (D). Immediately following 2 hours of inoculation, 10mg/mL of a neutralizing anti-SARS-CoV-2 spike antibody or 10mg/mL of an IgG isotype control antibody were added to the infected cell cultures. 24-hours post inoculation cell supernatants were collected for virus titer determination by TCID_50_/mL quantification. Cells were collected for RT-qPCR. Expression of host genes and viral genes/DVG-B were calculated relative to GAPDH. IL-29 relative copy number values were normalized to mock for A549:EV and A549:N, respectively. *p < 0.05, **p < 0.01, ***p < 0.001, ****p < 0.0001 by two-way ANOVA with Tukey’s multiple comparisons test. N = 3 biological repeats, mean±SD. Note that the SARS-CoV-2 MOI was reduced to MOI 0.1 in these experiments due to significantly greater cell death in the A549:N cells at MOI 1, likely due to enhanced gene expression.

To determine whether N complementation alters DVG-B–induced IFN responses, A549:EV and A549:N cells were infected with WT virus or DVG-B. RT-qPCR analysis revealed that in A549:EV cells, DVG-B induced higher IFNB1 and IL-29 expression than WT virus in both cell types, whereas the WT virus triggered minimal antiviral responses (Fig 7D). Unexpectedly, IFN responses were further increased in A549:N cells during DVG-B infection compared to A549:EV cells, correlating with increased viral RNA levels. Because complementation of the N protein to DVG-B infection now allows for virus particle formation, we hypothesized that the increase in IFN induction by DVG-B may be due to the formation and dissemination of infectious DVG-B particles. To address this, following SARS-CoV-2 or DVG-B inoculation, a spike neutralizing antibody (Anti-S) or IgG isotype control was added to infected cell cultures to prevent virus-particle-dependent spread. Similarly, we observed elevated IFN responses in A549:N cells compared to A549:EV cells at 24 hours post DVG-B infection in the presence of IgG (Fig 7E). As expected, neutralization successfully blocked the infectivity of virus particles in supernatants from both WT and DVG-B in A549:N cells (Fig 7E, titer). Neutralization, however, did not reduce IFN induction or viral RNA levels in DVG-B–infected cells (Fig 7E), suggesting that virus particle dissemination minimally contributes to the overall DVG-B RNA level even in the presence of N at 24 hpi. In contrast, for WT virus, neutralizing antibody treatment significantly reduced the levels of N and S in both cell lines, while IL-29 levels remained similar to mock. Overall, these data indicate that, in our system, complementation of the N protein alone was sufficient to remodel the distribution of dsRNA/DMVs during DVG-B infection. Despite these changes, N complementation was insufficient to reduce DVG-B-induced antiviral gene expression to a level similar to that of the WT virus.

### DVG-B exhibited reduced SMV abundance and a distinct spike protein distribution

To further assess intracellular membrane rearrangements during DVG-B infection, transmission electron microscopy (TEM) was performed on WT virus– and DVG-B–infected A549:EV and A549:N cells, respectively (Fig 8A). At 24 hours post WT infection, numerous single-membrane vesicles (SMVs; indicated by *) were observed in infected cells of both cell types. Some SMVs contained virus particle-like structures, suggesting that they are the sites of virus particle assembly. In contrast, DVG-B–infected A549:EV cells showed a marked reduction in SMV abundance, suggesting that DVG-B is defective in the formation or maintenance of the virus particle assembly site. Instead, these cells contained multiple large electron-dense membrane-bound structures with internal substructures, consistent with description of tertiary lysosomes (indicated by * in Fig 8A). In DVG-B–infected A549:N cells, however, SMV abundance was partially restored. Notably, although tertiary lysosome–like structures were predominantly observed in DVG-B–infected A549:EV cells, they were also detected, albeit less frequently, in DVG-B–infected A549:N cells. Interestingly, we did not observe SMVs or lysosome-like structures in mock-infected cells of either cell type, strongly suggesting that N is required, but independently insufficient, for SMV formation or maintenance. Together, our TEM data indicate that DVG-B is defective in SMV formation or maintenance, which can be partially rescued by N complementation.

**Fig 8:**
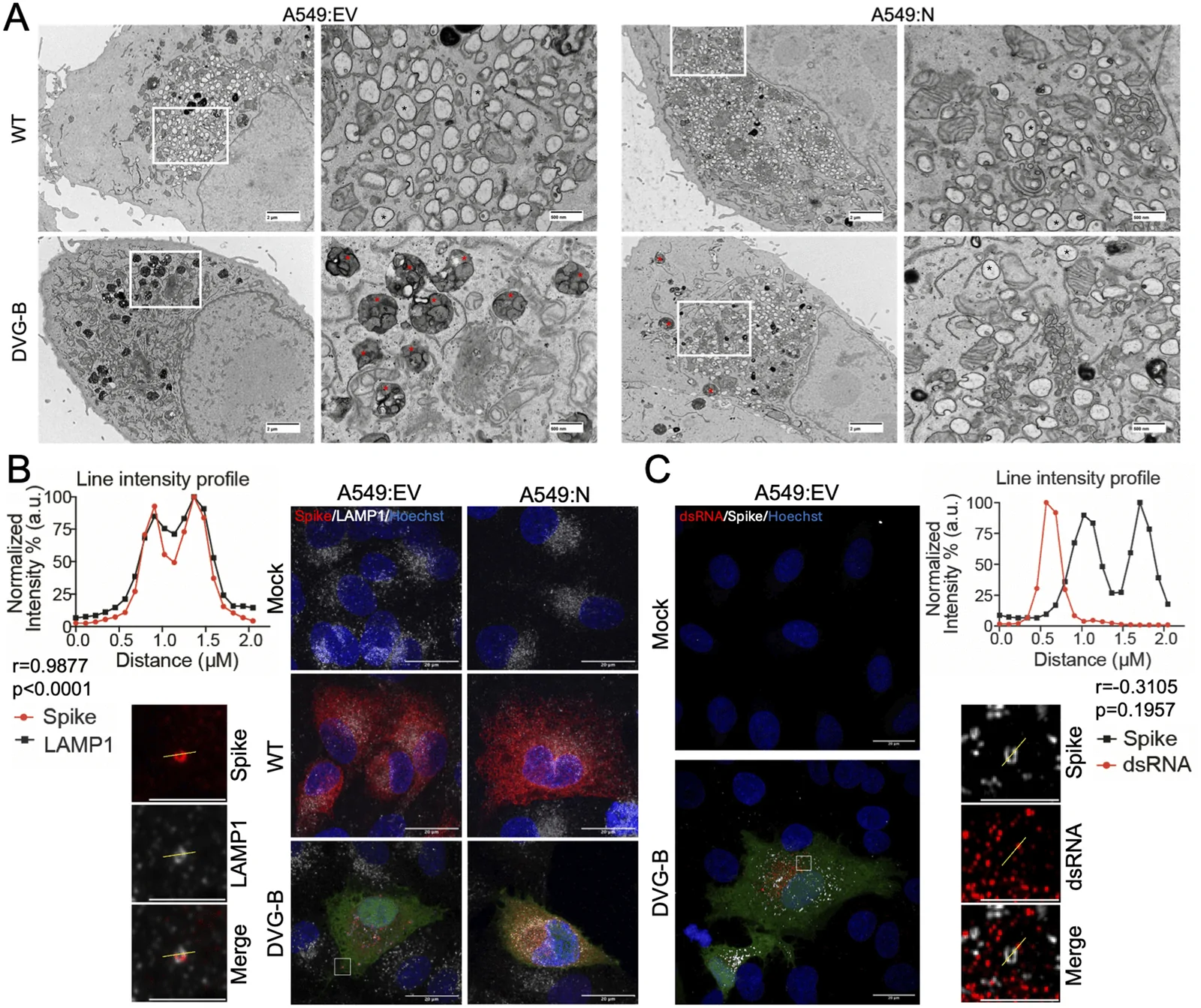
DVG-B exhibited reduced SMV abundance and a distinct spike distribution. (A) A549:EV (left) and A549:N cells (right) were infected with WT (MOI 1) or DVG-B (MOI 50 to maximize the number of DVG-B-infected cells), respectively, and cells were collected 24-hours post-inoculation for fixation and processing for transmission electron microscopy. Scale bars indicate 2μm. Insets depict magnified cell regions indicated by the white boxes. Scale bars indicate 500nm. Black asterisks indicate single membrane vesicles, red asterisks indicate lysosome-like structures. (B) A549:EV and A549:N cells were infected with WT (MOI 0.1) or DVG-B (MOI 1), respectively, and cells were collected 24-hours post-inoculation for confocal microscopy. Representative maximum intensity projections of merged spike (red) staining with the LAMP1 (white) in A549-EV and A549:N cells. Nuclei were Hoechst stained (blue). Scale bar indicates 20μm. Insets depict magnified cell regions indicated by the white boxes of spike and LAMP1 co-staining (left, below). Scale bar indicates 5μm. (Left, above) representative line intensity analysis of spike (red) and LAMP1 (black) co-occurrence. Spike and LAMP1 intensities normalized to their greatest, respective, pixel values along the path of the line (yellow, below) are depicted. Spearman’s correlation (r) value between normalized spike and LAMP1 and two-tailed p-value were shown. N = 10 spike and LAMP1 co-occurring structures. (C) A549:EV cells were infected with DVG-B (MOI 1) and cells were collected 24-hours post-inoculation for confocal microscopy. Representative maximum intensity projections of merged dsRNA (red) and spike (white) staining. Nuclei were Hoechst stained (blue). Scale bar indicates 20μm. Insets depict magnified cell regions indicated by the white boxes of dsRNA and spike co-staining (right, below). Scale bar indicates 5μm. (Right, above) representative line intensity analysis of dsRNA (red) and spike (black) at spike containing structures. dsRNA and spike intensities normalized to their greatest, respective, pixel value along the path of the line (yellow, below) were depicted. Spearman’s correlation (r) values between dsRNA and spike and two-tailed p-value were shown. N = 6 spike containing structures.

Because our TEM methods yielded results that were inherently qualitative, we sought to confirm the identity of the tertiary lysosome-like structures present in DVG-B-infected cells via immunofluorescence assays. Given the inverse relationship in the abundance of SMVs and tertiary-lysosome like structures in DVG-B-infected cells, we used spike and LAMP-1 to mark SMVs and lysosomes, respectively. At 24 hpi, WT virus-derived spike protein was observed widely distributed throughout the cell, likely marking the ER, ERGIC, exocytic vesicles and the plasma membrane. DVG-B-derived spike protein, however, was less abundant overall and localized to circular hollow structures additionally containing LAMP1 (Fig 8B). Line intensity analysis showed that spike and LAMP1 strongly correlated with one another in these structures (Fig 8B, left). While N protein complementation did not impact spike distribution in WT virus infected cells, among DVG-B-infected cells we observed that N largely restored the spike protein distribution to that of SARS-CoV-2. Notably, N protein complementation yielded a loss of these spike/LAMP1 containing-lysosomal structures during DVG-B infection. Furthermore, we determined that DVG-B-derived dsRNA localizes to a distinct compartment relative to spike containing lysosomes (Fig 8C). Overall, our data indicate that absence of the N protein drastically altered the cellular distribution of the spike protein and further suggest that the N protein is required for optimal SMV maintenance.

### Absence of the N protein is responsible for DVG-B’s dsRNA distribution pattern and IFN induction phenotypes

DVG-B lacks four proteins in addition to the absence of the N protein. To assess whether the lack of orf7a, orf7b, or orf8 contribute to DVG-B’s interferon induction activity or its dsRNA distribution profile, we generated SARS-CoV-2 VLPs packaging a recombinant SARS-CoV-2 lacking the N open reading frame but retaining orf7a, orf7b and orf8 (DVG-ΔN, Fig 9A). DVG-ΔN was passaged similarly as DVG-B in VeroE6:N cells. Deep sequencing of DVG-ΔN P3 stocks exhibited the introduced deletion when aligned to the WT viral reference genome (S5A Fig), whereas this deletion was not observed in the alignment using DVG-ΔN as the reference genome (S5B Fig). Additionally, DVG-ΔN showed comparable levels and similar distributions of additional deletions as DVG-B P3 (S5C Fig vs S3C Fig). Interestingly, the nsp12 point mutations observed in the DVG-B stock were again observed in the DVG-ΔN stock (S2 Table in Supporting Information). To compare the IFN-induction activity of DVG-ΔN and DVG-B, A549-ACE2 cells were infected with WT, DVG-B, or DVG-ΔN (MOI 1) for 24 hours. RT-qPCR showed that both DVG-B and DVG-ΔN, but not the WT virus, induced similar levels of IFNB1, IL-29, and ISG54 expression (Fig 9B). Consistently, bulk RNA-seq revealed highly similar host transcriptional responses between DVG-B and DVG-ΔN infected cells, with only 29 differentially expressed genes identified. PCA analysis showed that DVG-B and DVG-ΔN clustered together (S5E Fig, corresponding heatmap shown in S5F Fig). Confocal microscopy further showed a similar discrete dsRNA distribution pattern in DVG-B- and DVG-ΔN-infected cells (Fig 9C), indicating that deletion of the N open reading frame largely recapitulates the IFN induction and dsRNA distribution phenotypes observed during DVG-B infection. It should be noted that because orf9b is translated from an alternative start codon in N encoding mRNA, as a consequence of leaky ribosomal scanning, DVG-B and DVG-ΔN also lack orf9b [46]. Orf9b reportedly interacts with the Translocase of the Outer Mitochondrial Membrane protein 70 (TOM70) to suppress RLR-mediated IFN induction [47,48]. Thus, we sought to determine whether the complementation of orf9b impacts IFN induction during DVG-ΔN infection. A549-ACE2 cells were transduced with lentiviral vectors encoding SARS-CoV-2 orf9b or an empty lentiviral vector (EV) control for 48 hours (S5G Fig) followed by DVG-ΔN infection. 24 hours post DVG-ΔN infection, orf9b complementation yielded a slight reduction in IFNB1 and IL-29 expression, while no differences in ISG54 expression were observed compared to the EV transduced cells (Fig 9D). Overall, our data support the conclusion that while the absence of orf9b modestly contributes to DVG-B’s IFN induction activities, the loss of the N protein, rather than orf7a, orf7b, or orf8, is primarily responsible for DVG-B’s IFN induction activity and its distinct dsRNA distribution.

**Fig 9:**
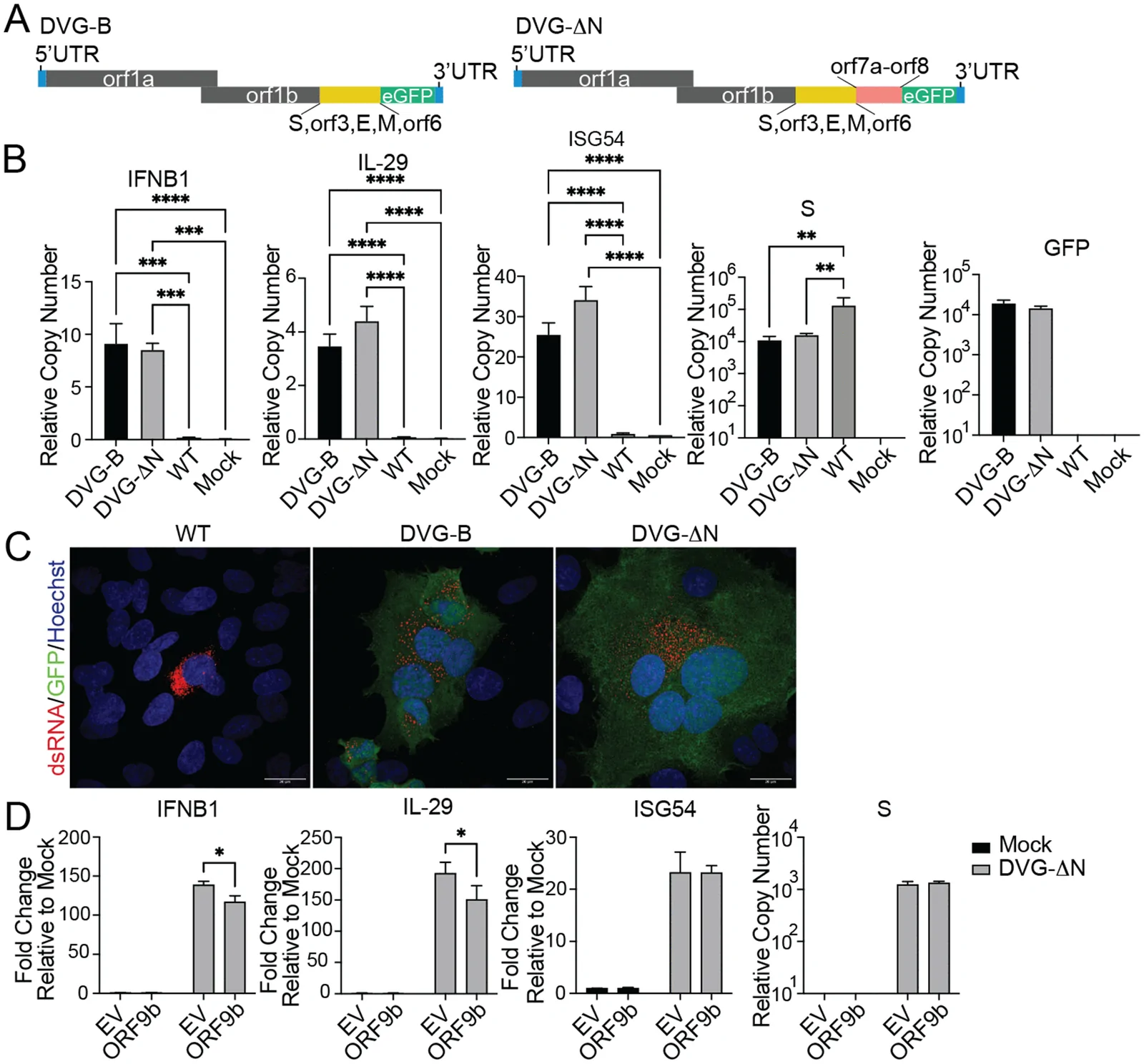
Absence of the N protein was primarily responsible for DVG-B’s dsRNA distribution pattern and IFN induction phenotypes. (A) Genome schematics of DVG-B (left) and DVG-ΔN (right). (B) A549-ACE2 cells were infected with WT, DVG-B or DVG-ΔN at MOI 1 and cells were collected 24-hours post-infection for RT-qPCR. Expression of host genes and viral genes/eGFP were calculated relative to GAPDH. **p < 0.01, ***p < 0.001, ****p < 0.0001 by one-way ANOVA with Tukey’s multiple comparisons test. N = 3, mean±SD. (C) A549-ACE2 cells were infected with WT (MOI 0.1), DVG-B or DVG-ΔN (MOI 1) and cells were collected 24-hours post-inoculation for confocal microscopy. Representative maximum intensity projections of merged dsRNA (red) staining. Nuclei were Hoechst stained (blue). GFP (green) indicates DVG-B and DVG-ΔN, respectively. Scale bar indicates 20μm. N = 4 fields for WT, DVG-B and DVG-ΔN, respectively. (D) A549-ACE2 cells were transduced with lentiviral vectors encoding SARS-CoV-2 orf9b (ORF9b) or an empty lentiviral vector control (EV). 48 hours post-transduction, cells were infected with DVG-ΔN (MOI 1). 24 hpi cells were collected for RT-qPCR. Expression of host and viral genes were calculated relative to GAPDH. *p < 0.05, by two-way ANOVA with Bonferroni’s multiple comparisons test. N = 3, mean±SD.

## Discussion

As DVG populations are heterogeneous and have multiple roles in viral pathogenesis, it is suspected that different DVG species may exert distinct functions during infection. In this study, we demonstrate that DVGs generated from different regions of the SARS-CoV-2 genome exhibit distinct capacities to stimulate IFN responses. Although both DVGs suppressed WT virus replication when supplemented at the onset of infection, DVG-A, generated from hotspot A, did not induce IFN responses, whereas DVG-B, generated from hotspot B, triggered robust IFN induction. Our data suggest that the IFN stimulation by DVG-B requires RNA-synthesis activity. In contrast, DVG-A’s inability to induce the IFN response is likely because it lacks the majority of the viral genome and therefore cannot independently replicate its genome. Due to this replicative defect, the abundance of DVG-A following supplementation was consistently lower than that of DVG-B, raising the possibility that insufficient DVG-A levels may account for the lack of IFN induction. To address this, we infected A549-ACE2 cells with DVG-A at an MOI of 80, exceeding the level of DVG-B detected in PCLS experiments, yet still did not observe any IFN stimulation compared to mock (S4E Fig). Consistently, during *in vitro* viral infection (Fig 2), cells positive for DVGs in hotspot A had a comparable level of DVGs compared to cells positive for DVGs in hotspot B, yet they exhibited a much lower level of IFN expression. Taken together, these findings strongly suggest that DVGs generated from hotspots A and B differ in their ability to stimulate IFN responses. Further *in vivo* studies will be required to validate this conclusion.

The distinct capacities of DVG-A and DVG-B to induce IFN suggest that these two DVGs suppress the WT virus through different mechanisms. Because DVG-B strongly induces IFN responses, its robust IFN induction likely contributes to the suppression of WT virus replication. In addition, although DVG-B can express nonstructural proteins, it lacks the essential assembly component, N. Therefore, DVG-B may compete with WT viral genomes for assembly machinery, thereby reducing overall WT virus production during coinfection. Further investigation of WT virus suppression by DVG-B in IFN-deficient cells, such as Vero E6 cells, will be needed to determine the relative contributions of IFN-dependent and IFN-independent mechanisms of DVG-B in suppressing WT virus replication. For DVG-A, our data clearly indicate that it suppresses WT virus through an IFN-independent mechanism. Consistent with this observation, a previous study demonstrated that a synthetic DVG with a genomic structure similar to DVG-A can be developed into a therapeutic interfering particle (TIP) that effectively suppresses wild-type SARS-CoV-2 infection in Syrian golden hamsters without inducing IFN responses [49]. The molecular mechanism by which DVG-A inhibits WT virus replication remains unknown. Because DVG-A is relatively short and retains both the 5′ and 3′ UTRs, it may compete with WT viral genomes for replication and assembly. DVG-A particles may alternatively compete with SARS-CoV-2 for ACE2 at entry. In addition, the DVG-A genome may form unique secondary structures and generate distinct siRNAs that could further target WT viral genomes.

We observed that DVG-B-derived dsRNA was distributed throughout the cytoplasm, whereas WT virus-derived dsRNA was primarily clustered in the perinuclear region. Both RIG-I and MDA5 play roles in detecting dsRNA to induce IFN expression via MAVS (Fig 6). Therefore, we initially hypothesized that this difference in dsRNA localization contributed to DVG-B’s robust IFN induction. Complementation of the N protein during DVG-B infection partially restored its dsRNA distribution to a pattern resembling that observed in WT virus infection and redistributed nsp6 and N into close proximity with dsRNA. This restoration, however, did not reduce IFN expression to levels observed during WT virus infection. Instead, N complementation further enhanced IFN responses induced by DVG-B compared to infection in the absence of N, suggesting a more complex relationship between dsRNA localization and innate immune sensing.

These observations raise several questions regarding the relationship between dsRNA distribution and RLR sensing. First, is RLR signaling saturated in our system? We consider this unlikely given that N complementation enhanced, rather than maintained, DVG-B-induced IFN responses, suggesting that RLR availability is not limiting under these conditions. Second, is dsRNA spatial organization uncoupled from RLR sensing? The rJ2 antibody used in this study recognizes a minimum dsRNA epitope of ~40 bp. Because SARS-CoV-2 generates dsRNAs that can reach several kilobases in length, our microscopy analyses are inherently biased toward detecting these long dsRNA species. It is unlikely that our immunofluorescent images fully reflect the subcellular distribution of shorter dsRNAs. Thus, the apparent disconnect between dsRNA localization and IFN induction may reflect differential sensing of distinct viral dsRNA ligands by RIG-I and MDA5.

MDA5 reportedly detects long dsRNAs (replication intermediates) generated during SARS-CoV-2 genomic replication [21,22,50]. However, our RIG-I and MDA5 knockdown data suggest that RIG-I is the major sensor for detecting DVG-B-derived dsRNA (Fig 6I). RIG-I preferentially recognizes short blunt-ended dsRNAs with a 5′-triphosphate or 5′-diphosphate, typically 10–300 bp in length [51,52]. Our data suggest that such short dsRNAs are the major IFN-inducing stimuli during DVG-B infection. Given the absence of N, RIG-I may detect short dsRNA species formed as a consequence of genomic RNA degradation following its export from the DMV. Like SARS-CoV-2, DVG-B’s long dsRNA replication intermediates may be detected by MDA5, whose access to such dsRNAs may be enhanced in the absence of N. While we consistently observed that N protein complementation redistributed the pattern of long dsRNA species detected by the rJ2 antibody during DVG-B infection, we suspect that the N protein also drove greater production of shorter dsRNA species, detected by RIG-I to further elevate IFN responses. Therefore, we hypothesize that the sensing of long dsRNAs, perhaps by MDA5, is coupled to their spatial organization. In contrast, RIG-I activation may depend on the abundance of short dsRNAs and be independent of the spatial distribution of long dsRNA species. Additionally, because our analyses assessed IFN induction at 24 hpi, it is possible that the relative contributions of RIG-I and MDA5 differ at different stages of viral infection. For example, at time points greater than 24 hpi, as dsRNA genomic replication intermediates accumulate, MDA5 may play a relatively larger role in IFN induction. Future studies are necessary to identify the specific DVG-B derived dsRNA species responsible for activating RIG-I and/or MDA5, determine how N protein complementation affects their abundance and localization, and clarify the relative contributions of RIG-I and MDA5 to IFN induction throughout the course of infection in the presence and absence of N.

DVG-B and DVG-ΔN induced comparable levels of IFN, strongly suggesting that their IFN-inducing activity is primarily attributable to the absence of the N protein. N protein antagonism of the RLR pathway has been previously described [53–56]. Interestingly, the level of N expression is also reported to impact N-mediated antagonism of IFN induction. While low doses of N suppress IFN and ISG induction, high doses of N promote their expression [57]. Consequently, it is possible that the N protein level in our A549:N cells inherently promote IFN induction during DVG-B infection, independent of enhanced viral RNA synthesis activity. Alternatively, it is possible that the kinetics of N protein production, and its accumulation over time, are important for its antagonism of IFN induction. To our knowledge, this is the first study demonstrating that the absence of N enhances IFN induction in the context of SARS-CoV-2 infection. Nonetheless, our observations support previous descriptions identifying the N protein as an antagonist of the RLR pathway.

Our data additionally highlight a critical role of N in viral structural protein trafficking and virus particle assembly. The large lysosome-like structures observed in DVG-B–infected cells are intriguing. The co-staining of spike and LAMP-1 revealed that DVG-B infection resulted in a markedly altered spike distribution pattern, characterized by hollow spike-containing structures associated with LAMP-1 staining. Given the striking reduction in SMV abundance, and the corresponding increase in these lysosomal-like structures, in DVG-B-infected cells compared to WT, we speculate that these structures form as a consequence of aberrant SMV formation or maintenance. Previous studies have reported that β-coronaviruses utilize a lysosomal Arl8b-dependent exocytic pathway for virus egress [58]. Additionally, SARS-CoV-2 protein-human protein interactome data has previously identified the post-ER trafficking proteins: GOLGA8F, GIPC2, and ARPC3 as N interactors [59]. It is therefore possible that the N protein plays a role in maintaining the stability of the SMV or regulating post-ERGIC pathways involved in SARS-CoV-2 virus particle trafficking. Consequently, loss of the N protein may ultimately result in localization of the SMV to the lysosome. To better assess how the N protein, or its lack thereof, impacts SMV formation and maintenance, future experiments may seek to evaluate their morphology at various time points post-infection and assess whether the viral envelope and membrane proteins also localize to the lysosome. Given the conservation of N among CoVs, this data raises the question as to whether those MERS-CoV or SARS-CoV DVGs lacking N show similar patterns of dsRNA spatial distributions, SMV dysregulation and lysosome-like structure formation [60]. Importantly, we found that DVG-B’s dsRNA puncta do not co-localize with spike containing lysosomes, suggesting that the N protein regulates dsRNA distribution and virus particle assembly as two separate functions.

Finally, we analyzed the DVG population in a COVID-19 cohort with reported disease severity scores. We observed a weak positive correlation between DVG abundance and disease severity. This modest association may reflect limitations of the dataset, including a relatively small sample size, a single time point of sample collection, and a cohort skewed toward severe cases. Nevertheless, approximately 40% of the DVG population mapped to hotspot B, indicating that this region is the dominant site of DVG generation compared with other genomic locations in patients. DVG+ patients also exhibited significantly higher viral loads than DVG- patients, suggesting that these DVGs likely arise as a consequence of high WT virus replication. In other words, in DVG+ elderly individuals with severe disease, higher levels of viral replication may lead to increased production of DVGs. Interestingly, our correlation analyses indicate that the association between DVG abundance and disease severity is stronger than that between viral load and disease severity (Fig 1D). These findings suggest that both elevated viral load and increased DVG levels may contribute to severe disease. Therefore, it is possible that DVGs are generated relatively late during infection in the elderly and their IFN-stimulating activity arises too late to effectively suppress WT virus replication. Instead, their presence may further amplify the inflammatory environment associated with high viral loads, thereby contributing to more severe disease outcomes. Consequently, it is important to emphasize that the association between DVG abundance and disease severity may be strictly correlative and does not necessarily constitute evidence that DVGs directly contribute to disease severity. Larger, longitudinal cohorts will be necessary to better define the relationship between *de novo* DVG generation and COVID-19 severity.

There are several limitations in this study. The representative DVG-A and DVG-B used in this study are synthetic and produced via different approaches. This raises the question as to whether these synthetic DVG-A and DVG-B species capture the diversity of naturally produced DVGs. In our previous study, we identified two major genomic regions, hotspot A and hotspot B, where DVG recombination sites cluster. Hotspot A is defined by a rejoin point (E’) ranging from 700 to 2500 and a break site (V’) from 28,200–29,750, whereas hotspot B includes a rejoin point from 27,700–29,400 with similar break sites. To capture the full range of DVGs within these two hotspots, we used the outer boundaries of these respective recombination junctions to generate representative DVGs, DVG-A (700–29,750) and DVG-B (27,700–29,750). To ensure efficient replication and packaging for downstream functional analysis, we retained a portion of the orf10 sequence (thereby changing the 5′ end to 29,674) and incorporated a PS9 packaging signal in DVG-A. Although our synthetic DVGs cannot represent the extensive diversity of DVG junctions generated during natural infection, we anticipate that they recapitulate the key functional properties of their respective hotspots. In particular, DVGs derived from hotspot B are expected to similarly induce IFN responses if N expression is absent. This is further supported by the similar IFN induction and dsRNA distribution between DVG-ΔN and DVG-B. Nonetheless, further validation using authentic DVGs is warranted. Future studies, such as enriching DVGs in WT virus stock through high-MOI passaging and constructing additional DVGs with different deletions identified from natural infection will help address this point.

DVG-A is a relatively minor species in patients compared to DVG-B, and its abundance during infection is substantially lower, suggesting that it plays a limited role in viral pathogenesis in the patient cohort analyzed here. While DVG-A is one of the major hotspots *in vitro*, we do not consider DVG-A a strictly tissue culture artifact. DVG-A can be consistently detected across multiple *in vitro* infection systems, including primary human bronchial epithelial cells and among different VOCs. DVG-A can be detected in some DVG+ patients with low abundance and is predominantly detected in infected cells rather than in supernatants [17]. These observations suggest that while DVGs in hotspot A are readily generated, they may be less efficient in packaging. Indeed, authentic DVG-A lacks the PS9 sequence, a reported packaging signal for SARS-CoV-2 [33]. This could result in less efficient dissemination and consequently a low abundance in clinical samples. Importantly, despite DVG-A’s low abundance in patient samples, it has great therapeutic potential. Our data show that DVG-A particles can suppress WT viral infection when administered concurrently and, notably, do not elicit strong IFN responses during infection. These data indicate that DVG-A suppresses WT virus replication through an IFN-independent mechanism. Consistent with this concept, synthetic SARS-CoV-2 RNAs with similar genomic structures have recently been developed as TIPs and shown to provide potent antiviral protection in the golden hamster model [24]. Thus, despite its low abundance in patients, the inclusion of DVG-A in this study provides valuable insights into its translational applications and underscores the need for further studies to elucidate the mechanisms by which it suppresses the WT virus.

While our VeroE6:N and A549:N cell systems yielded considerable shifts in the distribution of DVG-B-derived dsRNA, we consistently observed that N complementation failed to fully recapitulate the SARS-CoV-2 dsRNA pattern. This likely reflects fundamental differences between constitutive N expression and viral infection. Our cell lines may produce non-physiological levels of the N protein and inherently lack the regulated temporal control of N expression that occurs during SARS-CoV-2 infection. Moreover, N is normally expressed in concert with other viral proteins that collectively coordinate RO formation and membrane remodeling. Viral infection of a cell line with pre-existing levels of N may alter the efficiency of these coordinated viral processes limiting the ability of N complementation to fully restore the WT phenotype during DVG-B infection. This may also potentially explain why dsRNA and N colocalization was significantly reduced during SARS-CoV-2 WT virus infection in A549:N cells compared to A549:EV cells. For the same reason, the titer of DVG-B particles in A549:N cells was more than 3 logs lower than that of WT virus, suggesting suboptimal assembly during DVG-B infection even in the presence of N. Consequently, DVG-B particle release may be slower or less efficient, making early DVG-B dissemination less dependent on virus particle production.

In summary, our study provides direct evidence that SARS-CoV-2 DVGs generated from two distinct genomic regions exhibit different capacities to stimulate IFN responses and implicate the N protein as a key player impacting dsRNA/DMV distribution and SMV regulation. These findings provide a foundation for future studies to further elucidate the roles of DVGs in CoV pathogenesis and offer new insights into how DVGs may be harnessed as antiviral therapeutics.

## Materials and methods

### Cells, precision cut lung slices and viruses

All cell lines were cultured at 37°C with 5% CO_2_ in tissue culture media (TCM) containing Dulbecco’s Modified Eagle’s Media (DMEM, Gibco) with 10% fetal bovine serum (FBS), 1mM sodium pyruvate (Gibco), 2mM L-glutamate (Gibco) and 50μg/ml gentamicin (Gibco). A549-ACE2 cells were obtained from Dr. Ruth Serra-Moreno (URMC) and were cultured in TCM supplemented with 10μg/mL blasticidin (Gibco). VeroE6 cells constitutively expressing ACE2 and TMPRSS2 (VeroAT) were cultured in TCM supplemented with 10μg/mL puromycin. A549-ACE2: MAVS KO, PKR KO, and empty vector control cells were obtained from Dr. Susan R. Weiss [50] (University of Pennsylvania) and were maintained at 37°C with 5% CO_2_ in RPMI 1640 (Gibco catalog no. 11875–093) containing 10% FBS, 100U/ml penicillin, and 100μg/mL streptomycin. All cells were treated with mycoplasma removal agent before experimentation (MP Biomedicals). Human precision cut lung slices (PCLS) were obtained from LungMAP BRINDL biorepository (URMC) and were processed according to protocols published elsewhere [61,62]. All four PCLS donors were between 20–60 years of age. PCLS were maintained at 37°C with 5% CO_2_ in DMEM containing 10% FBS, 1 mM sodium pyruvate, 1X non-essential amino acids, 15 mM HEPES with 0.25μg/mL of amphotericin B (Gibco), and 80ng/mL gentamicin (Gibco). After 24 hours at 37°C, media was replaced and PCLS were incubated for an additional 24 hours prior to infection. SARS-CoV-2 hCoV-19/Hong Kong/VM20001061/2020 (HK, BEI NR-52282) was obtained through BEI resources. Viruses were propagated in VeroE6 cells in the presence of 5% FBS TCM. To limit the accumulation of DVGs, cells were infected at MOI 0.001 and virus containing supernatants were collected 3 days post-infection and filtered (0.45mm).

### DVG identification and association with IFN responses from bulk and scRNA-seq datasets

For Fig 2B-2D, we used the publicly available dataset from Ravindra et al. 2021 accessed through the NCBI database (GSE166766). This study consisted of single-cell RNA-seq (scRNA-seq) data from human bronchial epithelial cells (NHBEs) infected with SARS-CoV-2 that were harvested 1 day post infection (dpi), 2 dpi, and 3 dpi. The detailed approach for how we identify DVGs and DVG+ cells from scRNA-seq has been previously published elsewhere [17]. Briefly, we first used Cell Ranger [63] to construct gene expression matrices for each sample. To identify the number of viral transcripts, the SARS-CoV-2 reference sequence was concatenated to the end of the human genome reference as one additional gene. The gene expression matrices were then loaded into the Seurat package in R [64]. Cells were then clustered and annotated based on the gene markers used in the original publication of this dataset. To identify DVGs, we used UMI-tools [65] to associate the cell barcodes and UMIs with each corresponding read name. We used Bowtie2 [66], ViReMa, and a custom R filtering script for DVG identification. We then used the filtered ViReMa output to re-quantify DVG count based on the UMIs associated with each cell barcode. Specifically, we counted the number of unique UMIs with the same cell barcode as the total number of DVG reads per cell. The numbers of DVG UMIs of each cell barcode and the junction positions were then added to the gene expression matrix created by Cell Ranger. Cells with more than one DVG UMI (virus positive cells) were grouped as DVG + . Dot plots associating DVG junction positions and gene expression in DVG+ cells were graphed in R with the raw spreadsheet containing junction positions and IFN expression shown in S4 Table. Cells positive for DVG-A or DVG-B were defined based on the junction positions of the DVGs they contained. The boundaries of DVG-A and DVG-B were determined according to a previously published study [17]. Note that multiple DVGs can be present in one single cell (sharing the same cell barcode), and therefore a single DVG+ cell may be represented by multiple points corresponding to different DVG recombination sites in the plots. Cells containing multiple DVG species were counted multiple times in the comparison, regardless of whether the cells were positive for DVG-A or DVG-B. For bioinformatic analysis of the human cohort (Fig 1), we used the publicly available dataset (SCP1289 [30]). This study consisted of scRNA-seq data from a cohort study within known WHO severity scores (0–8) and age information. This cohort study consisted of nasal samples from 58 donors, 37 COVID-19 patients and 21 control patients. Within 37 COVID-19 patients, 23 individuals were less than 65 years of age (younger) and 14 elderly with age > 65. We used a similar approach, illustrated above, to identify DVGs from this scRNA-seq dataset. Patients with at least one unique DVG UMI were considered as DVG+ patients. The raw value of DVGs for individual patients were summarized in S3 Table.

For Fig 2E-2F, we used the publicly available dataset accessed through the NCBI database (GSE213759). This study consisted of bulk RNA-seq data from Calu3 cells infected with 2000 E copies of different SARS-CoV-2 variants of concern. Analyzed samples were infected cells collected at 24 – and 48-hpi. The detailed approach for how we identify DVGs from bulk RNA-seq has been previously published elsewhere [17]. Briefly, we first used Bowtie2 (v. 2.2.9 [66],) to align the reads to the GRCh38 human reference genome. The unmapped reads were then applied to ViReMa (Viral-Recombination-Mapper v. 0.21) to identify recombination junction sites and their corresponding read counts using SARS-CoV-2 reference genome (GenBank ID MT020881.1). A custom filtering script was developed in R to identify junction reads that met our criteria. We stipulated that the positions of both sites (break and rejoin) of junction reads are larger than 85 nts, as TRS-L is reported to be located with the first 85 nts of the SARS-CoV-2 genome. Additionally, we stipulated that deletions must be greater than 100 nts to ensure that the truncated viral RNAs are deficient in replication. We included all deletions with >10 counts. The number of viral reads (reads fully aligned to WT viral reference genome) in each bulk RNA-seq sample was quantified using the RSubread Bioconductor package. The junction frequency (Jfreq) was calculated by normalizing DVG counts to viral read counts.

### Generation of specific DVG particles and co-infection with WT virus

Representative DVG-A and DVG-B constructs were designed using the outermost deletion boundaries identified within hotspot A and hotspot B, respectively. The outermost boundaries were selected to capture a diversity of DVG recombination junctions thereby preserving the cumulative functional consequences associated with diverse sequence deletions in each DVG class. The DVG-A rejoin and break point boundaries are positions 700, located within the nsp1 gene sequence, and 29,674, within the 3’UTR, respectively. The 5′UTR–700 nt fragment (5′–700) and the 29,674–3′UTR fragment (29,674–3′) were amplified from CPER fragments [34] (kindly provided by Dr. Yoshiharu Matsuura of Osaka University). The mKate2 gene and the PS9 sequence (viral packaging signal) were inserted between the 5′–700 and 29,674–3′ fragments by overlapping PCR. The final construct was cloned into the pcDNA3.1 expression vector. To generate SARS-CoV-2 VLPs specifically packaging DVG-A, 3x10^6^ HEK293T cells were plated on poly-d-lysine (50μg/mL) treated 10 cm dishes 1 day prior to transfection. Each dish was transfected with 3.35μg pCDNA3.1-M-IRES-E (addgene #177938), 6.65μg pCDNA3.1-N (addgene #177937), 0.25μg pCDNA3.1-S (addgene #177939), and 10μg pCDNA3.1-DVG-A at a 3:1(v/w) PEI (1mg/mL):DNA(mg) ratio. Transfection complexes were added dropwise and cells were incubated for 24 hours at 37°C. 1-day post-transfection, the transfection media was aspirated and 10% TCM without antibiotics was added and cells were incubated for 24 hours at 37°C. 2 days post-transfection cell supernatants were collected and quick frozen, and 10% FBS TCM without antibiotics was added and cells were incubated for 24 hours at 37°C. Supernatant collection proceeded for 5 days. DVG-A containing supernatants were filtered (0.45μm) and centrifuged at 5000 x g, 4°C, for 16 hours. The DVG-A containing pellet was resuspended in 5% TCM and ultracentrifuged using an SW-32 Ti rotor (Beckman Coulter) through a sterile filtered 30% sucrose cushion (50mM Tris-HCL, 100mM NaCl, 50mM maleic acid, 5mM EDTA, in nuclease free water, pH = 6) at 100,000 x g, 4°C, for 2 hours. The DVG-A containing pellet was washed 5 times with HBSS prior to resuspension in 5% FBS TCM.

The generation of DVG-B was adapted from the CPER method to rescue recombinant SARS-CoV-2 infectious clones described by Torii et al.[34] and is derived from SARS-CoV-2/Hu/DP/Kng/19–020. Briefly, the orf7a-N deletion found in DVG-B was introduced into gene fragment F9/10 by replacing the orf7a-N genes with the enhanced green fluorescent protein (eGFP) gene under the N TRS-B sequence. To generate SARS-CoV-2 VLPs specifically packaging DVG-B, per well of a 6-well plate, half of the DVG-B CPER products (25μl), together with different combinations of pCDNA3.1-M-IRES-E (0.67μg), pCDNA3.1-N (1.32μg), or pCDNA3.1-S (0.02μg), were transfected into HEK293T cells using lipofectamine 2000 similarly as the approach used to package DVG-A. Supernatants were collected daily from days 2–8 post transfection. All supernatants were then pooled, filtered (0.45μm), and concentrated by centrifugation at 5000 x g 4°C, for 16 hours. Concentrated DVG-B was then serially passaged three times in VeroE6:N cells at MOI 0.001 for 5–6 days per passage. The working stock of DVG-B was purified via 30% sucrose cushion before usage.

To generate DVG-ΔN, the full-length genomic cDNA of SARS-CoV-2 was assembled into the bacterial artificial chromosome (BAC) backbone vector, pBeloBAC11. The pBeloBAC11 vector was modified to include a cytomegalovirus (CMV) immediate early promoter for transcription in mammalian cells, as well as yeast elements including the 2μ origin of replication and the URA3 selection cassette (promoter and marker), which were derived from the plasmid pYES2-Ub-M-GFP. The SARS-CoV-2 genome was placed under the control of the CMV promoter at the 5′ end. The 3′ terminus of the viral genome was engineered to include a 35-nucleotide poly(A) sequence, followed by the hepatitis delta virus ribozyme (HDVRz) and the bovine growth hormone (bGH) transcriptional terminator. The N gene was replaced by eGFP and full viral cDNAs were assembled from seven overlapping fragments. The modified pBeloBAC11 vector was amplified as two overlapping fragments by PCR (primers in S1 Table in Supporting Information). All DNA fragments were purified using a PCR purification kit (QIAquick PCR Purification Kit, Qiagen) according to the manufacturer’s instructions. The full-length pBeloBAC11-DVG-ΔN construct was assembled using a yeast-based homologous recombination system as published elsewhere [67]. DVG-ΔN plasmid was purified using a Qiagen plasmid extraction kit, followed by sequence confirmation via whole plasmid sequencing (Plasmidsaurus). To rescue the DVG-ΔN, 4 µg of plasmid DNA was transfected into VeroE6:N cells (5x10^5^ cells per plasmid) with Lipofectamine 2000 (3:1 plasmid to Lipofectamine 2000 ratio). The plasmid and Lipofectamine 2000 mix was incubated in Opti-MEM at room temperature for 30 minutes. Transfection complexes were then added to the cells in 10% FBS antibiotics-free TCM. 24 hours post-transfection the media was replaced with antibiotics-free 5% TCM. Cells were monitored for cytopathic effect (CPE) and GFP expression, and supernatants were harvested at ~50% CPE (approximately 15 days post-transfection). Supernatants were titered and passaged as DVG-B.

For infection, cells were inoculated with DVG-A, DVG-B, or DVG-ΔN alone, or co-infected with SARS-CoV-2 plus DVG-A or DVG-B, for 3 h at 37 °C in 5% FBS TCM, using the MOIs and DVG-to-SARS-CoV-2 MOI ratios specified in the corresponding figure legends. Each PCLS was inoculated with 1x10^5^ TCID_50_ SARS-CoV-2, 5x10^5^ FFU DVG-B or 2x10^6^ genome copy numbers of DVG-A, either alone or in combination, in PCLS maintenance media (5% FBS) for 3 hours at 37°C. Post inoculation, PCLS were washed three times with DPBS and cultured in 5% FBS PCLS maintenance medium. Supernatants and PCLS were harvested at 24- or 48 hpi. To harvest PCLS, tissues were homogenized at 4°C in 1mL Trizol and total RNA was extracted.

### Virus and DVG titration

SARS-CoV-2 titers were determined by median tissue culture infectious dose (TCID_50_). Briefly, VeroAT cells were infected with serially diluted viral supernatants and CPE was measured 3 days post-infection. TCID_50_/mL was quantified using the Reed-Muench method. DVG-B titers were calculated by determining the fluorescent forming units (FFU)/mL. Briefly, VeroE6:N cells were infected with serially diluted DVG containing supernatants and GFP+ cells were quantified 24 hours post-infection. To determine the DVG-A titer, total RNA was extracted from DVG-A stocks by Trizol-LS and purified RNA (2mg) was Turbo DNaseI (2U/mg RNA) treated at 37°C for 30 minutes. DVG-A copy number/mL was quantified relative to a standard curve of serially diluted DVG-A encoding plasmid by RT-qPCR.

### Remdesivir treatment

1 day prior to infection A549-ACE2 cells were treated with 10μM remdesivir (MedChemExpress catalog no. GS-5734) or and equal volume of DMSO in 10% TCM. Cells were inoculated with SARS-CoV-2 (Hong Kong, MOI 0.2) or DVG-B (MOI 1) in 5% TCM for 3 hours in the absence of remdesivir or DMSO. Following inoculation, 5% TCM containing 10μM remdesivir or an equal volume of DMSO was added to the cells. Alternatively, cells were transfected with 1μg/mL low molecular weight poly(I:C) (InvivoGen catalog no. tlrl-picw) via Lipofectamine 2000. 24 hours post-inoculation/transfection cells were lysed in Trizol for total RNA extraction or fixed in 4% paraformaldehyde for immunostaining. DVG-A (MOI 1) infections were performed identically followed by Trizol lysis for total RNA extraction.

### RNA extraction and RT-qPCR

Tissue culture cell lines/supernatants were lysed in Trizol or Trizol LS (Invitrogen) at the indicated timepoints and total RNA was extracted according to the manufacturer’s specifications, respectively. Total RNA (100ng-1μg) was reversed transcribed using the high-capacity RNA to cDNA kit (Applied Biosystems). cDNA was diluted and qPCR reactions were performed in triplicate using specific RT-qPCR primers (S1 Table in Supporting Information) and Power Track SYBR Green PCR Master Mix (Applied Biosystems) in a Viia7 Light-cycler (Applied Biosystems). Relative copy numbers for viral and host gene expression levels were calculated relative to GAPDH.

### RNA-seq of A549-ACE2 infections and DVG-B/DVG-ΔN stocks and host transcriptome analysis

RNA from SARS-CoV-2 and DVG infected A549-ACE2 cells were extracted, followed by DNase I digestion. 100–300 ng of total RNA was used for single-end bulk RNA-seq (Plasmidsaurus). Quality of the fastq files was assessed using FastQC v0.12.1. Reads were then quality filtered using fastp v0.24.0 with poly-X tail trimming, 3’ quality-based tail trimming, a minimum Phred quality score of 15, and a minimum length requirement of 50 bp. For transcriptome analyses, quality-filtered reads were aligned to the human reference genome (hg38) using bowtie2. Gene-level expression quantification was performed using featureCounts (subread package v2.1.1) with strand-specific counting, multi-mapping read fractional assignment, exons and three prime UTR as the feature identifiers, and grouped by gene_id. Final gene counts were annotated with gene biotype and other metadata extracted from the reference GTF file. Sample-to-sample correlations for heatmap generation and PCA were calculated on normalized counts (TMM, trimmed mean of M-values) using Pearson correlation. Differential expression was done with edgeR v4.0.16 using standard practice including filtering for low-expressed genes. Functional enrichment, when available, was performed using gene set enrichment analysis. Detailed differential gene lists for designated clusters were shown in S5-S9 Tables.

For P2 and P3 DVG-B and P3 DVG-ΔN stocks, total RNA was extracted using Trizol followed by Turbo DNaseI treatment. RNA quality was assessed using the RNA Pico 6000 module on an Agilent Tapestation 2100 (Agilent Technologies) prior to cDNA library preparation. 40–100 ng of total cDNA libraries were prepared using the Illumina TruSeq Stranded Total RNA LT kit with Ribo-Zero Gold, according to the manufacturer’s instructions. Samples were run on Illumina NextSeq 500 to generate 150 bp, paired-end reads, resulting in 20–50 million reads per sample, with an average Q30 score ≥ 96.8%. Reads were aligned to DVG-B reference genome and WT virus genome (MT020881). The resulting BAM files were used for visualization in IGV, and unmapped reads were analyzed using ViReMa for DVG identification, as described in the previous DVG identification section.

### Transduction of cells to constitutively express SARS-CoV-2 N and orf9b

VeroE6 cells constitutively expressing the SARS-CoV-2 N protein (VeroE6:N) were generated by lentiviral transduction using the pLVX-EF1a-IRES-Puro (Takara) lentiviral expression vector followed by puromycin selection and single clone propagation. VeroE6:N cells were cultured in 10% FBS tissue culture media supplemented with 8 mg/ml puromycin. A549-ACE2 cells constitutively expressing SARS-CoV-2 N (A549:N) or an empty vector control (A549:EV) were similarly generated by lentiviral transduction followed by puromycin selection and single clone propagation. A549:N and A549:EV cells were cultured in 10% TCM containing 10μg/ml blasticidin and 1.5μg/ml puromycin. A549-ACE2 cells constitutively expressing SARS-CoV-2 orf9b or an EV control were transduced with lentiviral vectors for 48 hours, in the absence of selection, prior to experimentation. Lentiviral constructs were obtained from addgene (https://doi.org/10.1038/s41586-020-2286-9).

### RNA interference

siRNAs for human RIG-I (23586, ON-TARGETplus 2.0 smart pool), siRNA for human MDA5 (64135, ON-TARGETplus 2.0 smart pool) and ON-TARGETplus 2.0 non-targeting control pool were purchased from GE health, Dharmacon. 10^5^ A549-ACE2 cells were transfected with 50 μM of different siRNAs with Lipofectamine RNAiMAX complexes (Invitrogen) according to the manufacturer’s instruction (reverse transfection). After 16 hours of incubation, media was replaced with TCM without antibiotics. 24 hours post-transfection, cells were infected with DVG-B (MOI 1) for 24 hours. Cells were harvested with either Trizol for RNA extraction or RIPA lysis buffer for protein analysis.

### Immunofluorescent assay, confocal microscopy, and image quantification

For widefield and confocal microscopy, at the indicated times post-infection, cells were fixed on #1.5 coverslips with 4% paraformaldehyde for 15 minutes at room temperature and washed 3 times with 1X PBS. Fixed cells were permeabilized and blocked in 1X PBS containing 0.3% Triton X-100 and 5% goat serum (Cell Signaling) for 1 hour at room temperature. Primary and secondary antibodies were diluted in 1X PBS containing 1% bovine serum albumin and 0.3% Triton X-100. Primary antibody incubations were carried out for 18 hours at 4°C. Secondary antibody incubations were carried out for 2 hours at room temperature. See S1 Table in Supporting Information for antibody concentrations. Cells were then incubated with 1μg/mL Hoechst (Invitrogen H3570, 10mg/mL) for 10 minutes at room temperature. Cover slips were mounted onto microscope slides using the ProLong Gold Antifade mountant (Invitrogen). Widefield images were taken with a Leica DMIRB inverted fluorescence microscope with a cooled charge-coupled device (Cooke) on Image-Pro Plus software (Media Cybernetics). The proportion of dsRNA+ cells containing nuclear IRF-3 in SARS-CoV-2 and DVG-B infections was quantified by normalizing the number of dsRNA+nuclear IRF-3 + cells to the total number of dsRNA+ cells per image. Confocal microscopy images of WT and DVG-B infected A549:EV and A549:N expressing cells were captured using a Nikon A1R HD laser scanning confocal microscope with TIRF using a 60X/1.49 oil objective. 405nm, 488nm, 561nm and 640nm excitation diodes to excite Hoechst, eGFP, dsRNA/N and N/nsp6, respectively. Confocal images of WT and DVG-B infected VeroAT and VeroE6:N expressing cells were captured using a Leica Stellaris 5 inverted confocal microscope with WLL using a 63X/1.40 oil objective. Widefield and confocal microscopy images were analyzed using Fiji/ImageJ. Pearson correlation (*R*) values were determined using the Coloc 2 Fiji plugin on the average-intensity projections of z-stacks obtained by confocal microscopy. Line intensity analysis was performed by normalizing signal intensity values to the greatest pixel value along the path of the line for spike and LAMP1, respectively.

### Transmission electron microscopy

Infected cells cultured on glass chamber slides were fixed in 0.1M Millonigs phosphate buffer containing 5% glutaraldehyde overnight at 4°C. The slides were washed 3X with 0.1M phosphate buffer, post-fixed for 20 minutes in buffered 1.0% osmium tetroxide/1.5% potassium ferrocyanide, dehydrated on the slides (chamber removed) through a graded series of ethanol (10-minute incubations) to 100%. Then 1:1 100% ethanol/Spurr’s resin for 15 minutes before being transferred to 100% Spurr’s epoxy resin twice, once for an hour and secondarily overnight. The next day, size 3 BEEM capsules were filled with fresh Spurr’s resin and inverted and placed over the cells of interest on the slides. The slides/capsules were polymerized for 24 hours at 65^0^C. The next day the slides were dipped several times in liquid nitrogen to break the surface tension between the glass and the polymerized capsules until they “popped off” the slide [68]. The capsules with the entrapped cells were trimmed down to a trapezoid and thin sectioned with a diamond knife at 70nm and placed onto 2x1 mm slotted formvar/carbon copper grids. The grids were stained with uranyl acetate and lead citrate and examined using a Hitachi (Pleasanton, CA) camera and an attached AMT Nanosprint (Woburn, MA) 12-megapixel digital camera.

### ELISA

IFNB1 concentrations within the supernatants of SARS-CoV-2, DVG-B and SARS-CoV-2/DVG-B co-infected cells were determined using the VeriKine-HS Human Interferon-Beta TCM ELISA Kit (PBL Assay Science catalog no. 41435) according to the manufacturer’s instructions. Absorbance at 450nm was quantified using a SpectraMax Mini multi-mode microplate reader. A 4-parameter logistic plot with 1/y^2^ weighted analysis was used for the results calculation.

### Neutralization assay

A549:EV and A549:N cells were infected with WT or DVG-B at designated MOIs similarly. Post inoculation, cells were washed three times with PBS and cultured in 5% TCM with 10μg/mL of spike neutralizing antibody (40592-R001, Sino Biological, obtained from BEI resources). Both supernatants and infected cells were harvested 24 hpi.

### Statistical analysis

All statistical analyses were performed with GraphPad Prism version 5.0 (GraphPad Software, San Diego, CA) and *R* v*3.4.1*. A statistically significant difference was defined as a *p*-value <0.05 and was determined using one-way or two-way analysis of variance (ANOVA) with a *post hoc* test to correct for multiple comparisons, or Student’s t-test, as specified in the corresponding figure legends.

## Supporting information

S1 Fig DVGs clustered in hotspot A and B showed different IFN induction ability via single-cell RNA-seq. (A) Gene expression of IFNB1, IL-29, and Mx1 were correlated with DVG junction positions at the single cell level at 3 dpi in NHBE cells (GSE166766). (B) At 3 dpi, gene expression of IL-29 and Mx1 were correlated with DVG junction positions at the single cell level in three major cell types positive for DVGs. Each dot represents an individual DVG+ cell. Both the color and size of dots are based on the expression level of indicated genes. Green brackets indicate the previously identified hotspot A and B. (C) Plots the correlation between viral reads (left) or DVG junction reads (right) and DVG junction positions. Each dot represents an individual DVG+ cell. Both the color and size of dots are based on the abundance of either viral reads or DVG junction reads.
(TIF)

S2 Fig Transcriptome profiling of WT virus and DVG infected A549-ACE2 cells. (A) PCA plot of DVG-A, DVG-B and SARS-CoV-2 single infections or DVG-A and DVG-B co-infections with SARS-CoV-2. (B) Specific genes that are differentially expressed between DVG-A and WT. All DEGs were identified using criteria ≥two-fold and ≤ 1% false discovery rate.
(TIF)

S3 Fig RNA sequencing analyses of DVG-B stocks. DVG-B P2 and P3 were used for deep sequencing. (A-B) RNA-seq reads from P2 and P3 DVG-B stocks were aligned to the WT SARS-CoV-2 genomic reference (A) or the DVG-B reference genome (B), respectively, via subread. Viral read coverage was graphed via IGV. Mutations accounting for more than 20% of total reads aligned to that position are colored. Red square indicates the expected deletion in DVG-B. (C) J_freq_ percentages in the P2 and P3 DVG-B stocks. (D) Dot plots illustrating additional deletions identified in the P2 and P3 DVG-B stocks. (E) A549-ACE2 cells were infected with P2 DVG-B and two different stocks of P3 DVG-B at MOI 1. Infected cells were harvested 24 hpi to examine the expression of IL-29 by RT-qPCR. (F) Estimated Vp activity was measured by calculating the firefly-to Renilla ratio in the presence of WT and nsp12 mutant Vp.
(TIF)

S4 Fig Validation of DVG-B’s dysregulated dsRNA distribution phenotype. (A) VeroAT and VeroE6:N cells were infected with WT (MOI 1) or DVG-B (MOI 1) and cells were collected 24 hpi for confocal microscopy. Maximum intensity projections of dsRNA (red) staining and merged images of dsRNA and the N protein (white). Nuclei were Hoechst stained (blue). Scale bar indicates 10μm. (B) Widefield microscopy image of single clone selected A549:N cells with anti-Strep tag II staining. Scale bar indicates 25μm. (C) A549:N cells were infected with WT (MOI 0.1) or DVG-B (MOI 1) and cells were collected 24-hours post-inoculation for confocal microscopy. Maximum intensity projections of merged N protein (red) staining with nsp6 (white). Nuclei were Hoechst stained (blue). Note that DVG-B encoded GFP is depicted (green). Scale bar indicates 20μm. Insets depict magnified cell regions indicated by the white boxes of N and nsp6 co-staining. Scale bar indicates 5μm. Graph indicates the Pearson’s correlation coefficient (*R)* values for the co-localization of N and Nsp6. Individual values are shown. **p < 0.01 by one-way ANOVA with Tukey’s multiple comparisons test. Mean±SD. (D) A549-ACE2 cells were transduced to constitutively express the SARS-CoV-2 N lacking any protein tag (A549:N:NPT) and transductants were single clone propagated. Cells were infected with WT (MOI 0.1) or DVG-B (MOI 1) and cells were collected 24-hours post-inoculation for widefield microscopy. dsRNA (red) and N (white) merged and single channel images are shown. Nuclei were Hoechst stained (blue). Note that DVG-B encoded GFP is depicted (green). Scale bar indicates 20μm. (E) A549-ACE2 cells were infected with DVG-A (MOI 80), DVG-A levels and IFNB1 were examined via RT-qPCR at 24 hpi.
(TIF)

S5 Fig RNA sequencing analyses of DVG-ΔN stock and transcriptome profiling of DVG-ΔN infected A549-ACE2 cells. DVG-ΔN P3 were used for deep sequencing. (A-B) RNA-seq reads from P3 DVG-ΔN stock were aligned to the WT SARS-CoV-2 genomic reference (A) or the DVG-ΔN reference genome (B), respectively, via bowtie2. Viral read coverage was graphed via IGV. Mutations accounting for more than 50% of total reads aligned to that position are colored. The black square indicates the expected deletion in DVG-ΔN. (C) J_freq_ percentages in the P3 DVG-ΔN stock (average of R1 and R2). (D) Representative dot plot of additional deletions (positive sense) identified in P3 DVG-ΔN R1. (E-F) A549-ACE2 cells were infected with DVG-ΔN, DVG-B, or WT. Infected cells were harvested at 24 hpi, followed by deep sequencing. PCA plot (E) and specific genes that are differentially expressed between DVG-B and DVG-ΔN (F) are shown. All DEGs are identified using criteria ≥two-fold and ≤ 1% false discovery rate. (G) Representative image of A549:EV and A549:ORF9b cells. A549-ACE2 cells were transduced with lentiviral vectors encoding a strep II tagged SARS-CoV-2 orf9b or an empty lentiviral vector control. 48 hours post-transduction, cells were collected for widefield microscopy staining for the strep II tag (green). Scale bar indicates 100μm. N = 5 fields.
(TIF)

S6 Fig Raw images. (A) The original, uncropped Western blot images corresponding to Fig 3E are shown. The upper image was exposed for a longer duration to visualize spike protein expression, whereas the lower image shows the same membrane with a shorter exposure time to better visualize GAPDH expression across all samples. The samples labeled as “X” were not a part of this experiment and are therefore not included in the analysis. (B-C) The original, uncropped Western blot images corresponding to Fig 6J are shown.
(TIF)

S1 Table Oligo primer and antibody list. (DOCX)

S2 Table Mutations identified in DVG-B and DVG-ΔN stocks. (DOCX)

S3 Table Total DVG abundance and individual species identified from Cohort analysis. (TXT)

S4 Table DVG junctions associated with their single-cell IFN expression at 2 dpi. (CSV)

S5 Table DEG lists of different clusters from Fig 5. (TXT)

S6 Table DEG lists of different clusters from Fig 5. (TXT)

S7 Table DEG lists of different clusters from Fig 5. (TXT)

S8 Table DEG lists of different clusters from Fig 5. (TXT)

S9 Table DEG lists of different clusters from Fig 5. (TXT)

## References

[1] Von Magnus P. Studies on interferenee in experimental influenza. I. Biological observations. Arkiv Kemi, Mineralogi Och Geologi. 1947;7.

[2] Li D, Lott WB, Lowry K, Jones A, Thu HM, Aaskov J. Defective interfering viral particles in acute dengue infections. PLoS One. 2011;6(4):e19447. doi: 10.1371/journal.pone.0019447 21559384 PMC3084866

[3] Calain P, Monroe MC, Nichol ST. Ebola virus defective interfering particles and persistent infection. Virology. 1999;262(1):114–28. doi: 10.1006/viro.1999.9915 10489346

[4] Von Magnus P. Incomplete forms of influenza virus. Adv Virus Res. 1954;2:59–79. doi: 10.1016/s0065-3527(08)60529-1 13228257

[5] Calain P, Roux L. Generation of measles virus defective interfering particles and their presence in a preparation of attenuated live-virus vaccine. J Virol. 1988;62(8):2859–66. doi: 10.1128/JVI.62.8.2859-2866.1988 3392771 PMC253722

[6] Treuhaft MW, Beem MO. Defective interfering particles of respiratory syncytial virus. Infect Immun. 1982;37(2):439–44. doi: 10.1128/iai.37.2.439-444.1982 6288562 PMC347553

[7] Girgis S, Xu Z, Oikonomopoulos S, Fedorova AD, Tchesnokov EP, Gordon CJ, et al. Evolution of naturally arising SARS-CoV-2 defective interfering particles. Commun Biol. 2022;5(1):1140. doi: 10.1038/s42003-022-04058-5 36302891 PMC9610340

[8] Tapia K, Kim W-K, Sun Y, Mercado-López X, Dunay E, Wise M, et al. Defective viral genomes arising in vivo provide critical danger signals for the triggering of lung antiviral immunity. PLoS Pathog. 2013;9(10):e1003703. doi: 10.1371/journal.ppat.1003703 24204261 PMC3814336

[9] Sun Y, Jain D, Koziol-White CJ, Genoyer E, Gilbert M, Tapia K, et al. Immunostimulatory defective viral genomes from respiratory syncytial virus promote a strong innate antiviral response during infection in mice and humans. PLoS Pathog. 2015;11(9):e1005122. doi: 10.1371/journal.ppat.1005122 26336095 PMC4559413

[10] Johnston MD. The characteristics required for a Sendai virus preparation to induce high levels of interferon in human lymphoblastoid cells. J Gen Virol. 1981;56(Pt 1):175–84. doi: 10.1099/0022-1317-56-1-175 6170730

[11] Marcus PI, Gaccione C. Interferon induction by viruses. XIX. Vesicular stomatitis virus--New Jersey: high multiplicity passages generate interferon-inducing, defective-interfering particles. Virology. 1989;171(2):630–3. doi: 10.1016/0042-6822(89)90637-5 2474895

[12] Strähle L, Marq J-B, Brini A, Hausmann S, Kolakofsky D, Garcin D. Activation of the beta interferon promoter by unnatural Sendai virus infection requires RIG-I and is inhibited by viral C proteins. J Virol. 2007;81(22):12227–37. doi: 10.1128/JVI.01300-07 17804509 PMC2169027

[13] Yount JS, Gitlin L, Moran TM, López CB. MDA5 participates in the detection of paramyxovirus infection and is essential for the early activation of dendritic cells in response to Sendai Virus defective interfering particles. J Immunol. 2008;180(7):4910–8. doi: 10.4049/jimmunol.180.7.4910 18354215

[14] Shingai M, Ebihara T, Begum NA, Kato A, Honma T, Matsumoto K, et al. Differential type I IFN-inducing abilities of wild-type versus vaccine strains of measles virus. J Immunol. 2007;179(9):6123–33. doi: 10.4049/jimmunol.179.9.6123 17947687

[15] Felt SA, Sun Y, Jozwik A, Paras A, Habibi MS, Nickle D, et al. Detection of respiratory syncytial virus defective genomes in nasal secretions is associated with distinct clinical outcomes. Nat Microbiol. 2021;6(5):672–81. doi: 10.1038/s41564-021-00882-3 33795879 PMC9098209

[16] Xu J, Sun Y, Li Y, Ruthel G, Weiss SR, Raj A, et al. Replication defective viral genomes exploit a cellular pro-survival mechanism to establish paramyxovirus persistence. Nat Commun. 2017;8(1):799. doi: 10.1038/s41467-017-00909-6 28986577 PMC5630589

[17] Zhou T, Gilliam NJ, Li S, Spandau S, Osborn RM, Connor S, et al. Generation and functional analysis of defective viral genomes during SARS-CoV-2 infection. mBio. 2023;14(3):e0025023. doi: 10.1128/mbio.00250-23 37074178 PMC10294654

[18] Llanes A, Restrepo CM, Caballero Z, Rajeev S, Kennedy MA, Lleonart R. Betacoronavirus genomes: how genomic information has been used to deal with past outbreaks and the COVID-19 pandemic. Int J Mol Sci. 2020;21(12). doi: 10.3390/ijms21124546 32604724 PMC7352669

[19] Perlman S, Netland J. Coronaviruses post-SARS: update on replication and pathogenesis. Nat Rev Microbiol. 2009;7(6):439–50. doi: 10.1038/nrmicro2147 19430490 PMC2830095

[20] Magnen M, You R, Rao AA, Davis RT, Rodriguez L, Bernard O, et al. Immediate myeloid depot for SARS-CoV-2 in the human lung. Sci Adv. 2024;10(31):eadm8836. doi: 10.1126/sciadv.adm8836 39083602 PMC11290487

[21] Yin X, Riva L, Pu Y, Martin-Sancho L, Kanamune J, Yamamoto Y, et al. MDA5 governs the innate immune response to SARS-CoV-2 in lung epithelial cells. Cell Rep. 2021;34(2):108628. doi: 10.1016/j.celrep.2020.108628 33440148 PMC7832566

[22] Minkoff JM, tenOever B. Innate immune evasion strategies of SARS-CoV-2. Nat Rev Microbiol. 2023;21(3):178–94. doi: 10.1038/s41579-022-00839-1 36631691 PMC9838430

[23] Hadjadj J, Yatim N, Barnabei L, Corneau A, Boussier J, Smith N, et al. Impaired type I interferon activity and inflammatory responses in severe COVID-19 patients. Science. 2020;369(6504):718–24. doi: 10.1126/science.abc6027 32661059 PMC7402632

[24] Chaturvedi S, Vasen G, Pablo M, Chen X, Beutler N, Kumar A, et al. Identification of a therapeutic interfering particle-A single-dose SARS-CoV-2 antiviral intervention with a high barrier to resistance. Cell. 2021;184(25):6022-6036.e18. doi: 10.1016/j.cell.2021.11.004 34838159 PMC8577993

[25] Rand U, Kupke SY, Shkarlet H, Hein MD, Hirsch T, Marichal-Gallardo P, et al. Antiviral activity of influenza A virus defective interfering particles against SARS-CoV-2 replication in vitro through stimulation of innate immunity. Cells. 2021;10(7):1756. doi: 10.3390/cells10071756 34359926 PMC8303422

[26] Xiao Y, Lidsky PV, Shirogane Y, Aviner R, Wu C-T, Li W, et al. A defective viral genome strategy elicits broad protective immunity against respiratory viruses. Cell. 2021;184(25):6037-6051.e14. doi: 10.1016/j.cell.2021.11.023 34852237 PMC8598942

[27] Wong CH, Ngan CY, Goldfeder RL, Idol J, Kuhlberg C, Maurya R, et al. Reduced subgenomic RNA expression is a molecular indicator of asymptomatic SARS-CoV-2 infection. Commun Med (Lond). 2021;1:33. doi: 10.1038/s43856-021-00034-y 35602196 PMC9053197

[28] Gribble J, Stevens LJ, Agostini ML, Anderson-Daniels J, Chappell JD, Lu X, et al. The coronavirus proofreading exoribonuclease mediates extensive viral recombination. PLoS Pathog. 2021;17(1):e1009226. doi: 10.1371/journal.ppat.1009226 33465137 PMC7846108

[29] Brennan JW, Sun Y. Defective viral genomes: advances in understanding their generation, function, and impact on infection outcomes. mBio. 2024;15(5):e0069224. doi: 10.1128/mbio.00692-24 38567955 PMC11077978

[30] Ziegler CGK, Miao VN, Owings AH, Navia AW, Tang Y, Bromley JD, et al. Impaired local intrinsic immunity to SARS-CoV-2 infection in severe COVID-19. Cell. 2021;184(18):4713-4733.e22. doi: 10.1016/j.cell.2021.07.023 34352228 PMC8299217

[31] Ravindra NG, Alfajaro MM, Gasque V, Huston NC, Wan H, Szigeti-Buck K, et al. Single-cell longitudinal analysis of SARS-CoV-2 infection in human airway epithelium identifies target cells, alterations in gene expression, and cell state changes. PLoS Biol. 2021;19(3):e3001143. doi: 10.1371/journal.pbio.3001143 33730024 PMC8007021

[32] Bouhaddou M, Reuschl A-K, Polacco BJ, Thorne LG, Ummadi MR, Ye C, et al. SARS-CoV-2 variants evolve convergent strategies to remodel the host response. Cell. 2023;186(21):4597-4614.e26. doi: 10.1016/j.cell.2023.08.026 37738970 PMC10604369

[33] Syed AM, Taha TY, Tabata T, Chen IP, Ciling A, Khalid MM, et al. Rapid assessment of SARS-CoV-2-evolved variants using virus-like particles. Science. 2021;374(6575):1626–32. doi: 10.1126/science.abl6184 34735219 PMC9005165

[34] Torii S, Ono C, Suzuki R, Morioka Y, Anzai I, Fauzyah Y, et al. Establishment of a reverse genetics system for SARS-CoV-2 using circular polymerase extension reaction. Cell Rep. 2021;35(3):109014. doi: 10.1016/j.celrep.2021.109014 33838744 PMC8015404

[35] Pechous RD, Malaviarachchi PA, Banerjee SK, Byrum SD, Alkam DH, Ghaffarieh A, et al. An ex vivo human precision-cut lung slice platform provides insight into SARS-CoV-2 pathogenesis and antiviral drug efficacy. J Virol. 2024;98(7):e0079424. doi: 10.1128/jvi.00794-24 38940558 PMC11265413

[36] Robinson JT, Thorvaldsdóttir H, Winckler W, Guttman M, Lander ES, Getz G, et al. Integrative genomics viewer. Nat Biotechnol. 2011;29(1):24–6. doi: 10.1038/nbt.1754 21221095 PMC3346182

[37] Offersgaard A, Duarte Hernandez CR, Pihl AF, Costa R, Venkatesan NP, Lin X, et al. SARS-CoV-2 production in a scalable high cell density bioreactor. Vaccines (Basel). 2021;9(7):706. doi: 10.3390/vaccines9070706 34209694 PMC8310283

[38] Gao Y, Yan L, Huang Y, Liu F, Zhao Y, Cao L, et al. Structure of the RNA-dependent RNA polymerase from COVID-19 virus. Science. 2020;368(6492):779–82. doi: 10.1126/science.abb7498 32277040 PMC7164392

[39] Luo Y, Yu F, Zhou M, Liu Y, Xia B, Zhang X, et al. Engineering a reliable and convenient SARS-CoV-2 replicon system for analysis of viral RNA synthesis and screening of antiviral inhibitors. mBio. 2021;12(1):e02754-20. doi: 10.1128/mBio.02754-20 33468688 PMC7845634

[40] Gu L, Wang Z-J, Zhang X-R, Liu Y, Zhao M, Jiang S-Z, et al. Targeting the liquid-liquid phase separation of nucleocapsid broadly inhibits the replication of SARS-CoV-2 strains. Biochem Biophys Res Commun. 2025;756:151594. doi: 10.1016/j.bbrc.2025.151594 40086356

[41] Zheng Y, Deng J, Han L, Zhuang M-W, Xu Y, Zhang J, et al. SARS-CoV-2 NSP5 and N protein counteract the RIG-I signaling pathway by suppressing the formation of stress granules. Signal Transduct Target Ther. 2022;7(1):22. doi: 10.1038/s41392-022-00878-3 35075101 PMC8785035

[42] Bessa LM, Guseva S, Camacho-Zarco AR, Salvi N, Maurin D, Perez LM, et al. The intrinsically disordered SARS-CoV-2 nucleoprotein in dynamic complex with its viral partner nsp3a. Sci Adv. 2022;8(3):eabm4034. doi: 10.1126/sciadv.abm4034 35044811 PMC8769549

[43] Cubuk J, Alston JJ, Incicco JJ, Singh S, Stuchell-Brereton MD, Ward MD, et al. The SARS-CoV-2 nucleocapsid protein is dynamic, disordered, and phase separates with RNA. Nat Commun. 2021;12(1):1936. doi: 10.1038/s41467-021-21953-3 33782395 PMC8007728

[44] Desmyter J, Melnick JL, Rawls WE. Defectiveness of interferon production and of rubella virus interference in a line of African green monkey kidney cells (Vero). J Virol. 1968;2(10):955–61. doi: 10.1128/JVI.2.10.955-961.1968 4302013 PMC375423

[45] Ricciardi S, Guarino AM, Giaquinto L, Polishchuk EV, Santoro M, Di Tullio G, et al. The role of NSP6 in the biogenesis of the SARS-CoV-2 replication organelle. Nature. 2022;606(7915):761–8. doi: 10.1038/s41586-022-04835-6 35551511 PMC7612910

[46] Xu K, Zheng B-J, Zeng R, Lu W, Lin Y-P, Xue L, et al. Severe acute respiratory syndrome coronavirus accessory protein 9b is a virion-associated protein. Virology. 2009;388(2):279–85. doi: 10.1016/j.virol.2009.03.032 19394665 PMC7103405

[47] Gao X, Zhu K, Qin B, Olieric V, Wang M, Cui S. Crystal structure of SARS-CoV-2 Orf9b in complex with human TOM70 suggests unusual virus-host interactions. Nat Commun. 2021;12(1):2843. doi: 10.1038/s41467-021-23118-8 33990585 PMC8121815

[48] Jiang H-W, Zhang H-N, Meng Q-F, Xie J, Li Y, Chen H, et al. SARS-CoV-2 Orf9b suppresses type I interferon responses by targeting TOM70. Cell Mol Immunol. 2020;17(9):998–1000. doi: 10.1038/s41423-020-0514-8 32728199 PMC7387808

[49] Chaturvedi S, Vasen G, Pablo M, Chen X, Beutler N, Kumar A, et al. Identification of a therapeutic interfering particle-A single-dose SARS-CoV-2 antiviral intervention with a high barrier to resistance. Cell. 2021;184(25):6022-6036.e18. doi: 10.1016/j.cell.2021.11.004 34838159 PMC8577993

[50] Li Y, Renner DM, Comar CE, Whelan JN, Reyes HM, Cardenas-Diaz FL, et al. SARS-CoV-2 induces double-stranded RNA-mediated innate immune responses in respiratory epithelial-derived cells and cardiomyocytes. Proc Natl Acad Sci U S A. 2021;118(16):e2022643118. doi: 10.1073/pnas.2022643118 33811184 PMC8072330

[51] Pichlmair A, Schulz O, Tan CP, Näslund TI, Liljeström P, Weber F, et al. RIG-I-mediated antiviral responses to single-stranded RNA bearing 5’-phosphates. Science. 2006;314(5801):997–1001. doi: 10.1126/science.1132998 17038589

[52] Schlee M, Roth A, Hornung V, Hagmann CA, Wimmenauer V, Barchet W, et al. Recognition of 5’ triphosphate by RIG-I helicase requires short blunt double-stranded RNA as contained in panhandle of negative-strand virus. Immunity. 2009;31(1):25–34. doi: 10.1016/j.immuni.2009.05.008 19576794 PMC2824854

[53] Aloise C, Schipper JG, van Vliet A, Oymans J, Donselaar T, Hurdiss DL, et al. SARS-CoV-2 nucleocapsid protein inhibits the PKR-mediated integrated stress response through RNA-binding domain N2b. PLoS Pathog. 2023;19(8):e1011582. doi: 10.1371/journal.ppat.1011582 37607209 PMC10473545

[54] Gori Savellini G, Anichini G, Gandolfo C, Cusi MG. SARS-CoV-2 N protein targets TRIM25-mediated RIG-I activation to suppress innate immunity. Viruses. 2021;13(8):Epub 2021-07-23. doi: 10.3390/v13081439 34452305 PMC8402637

[55] Oh SJ, Shin OS. SARS-CoV-2 nucleocapsid protein targets RIG-I-like receptor pathways to inhibit the induction of interferon response. Cells. 2021;10(3):530. doi: 10.3390/cells10030530 33801464 PMC7999926

[56] Chen K, Xiao F, Hu D, Ge W, Tian M, Wang W, et al. SARS-CoV-2 nucleocapsid protein interacts with RIG-I and represses RIG-mediated IFN-β production. Viruses. 2020;13(1):47. doi: 10.3390/v13010047 33396605 PMC7823417

[57] Zhao Y, Sui L, Wu P, Wang W, Wang Z, Yu Y, et al. A dual-role of SARS-CoV-2 nucleocapsid protein in regulating innate immune response. Signal Transduct Target Ther. 2021;6(1):331. doi: 10.1038/s41392-021-00742-w 34471099 PMC8409078

[58] Ghosh S, Dellibovi-Ragheb TA, Kerviel A, Pak E, Qiu Q, Fisher M, et al. β-Coronaviruses Use Lysosomes for Egress Instead of the Biosynthetic Secretory Pathway. Cell. 2020;183(6):1520-1535.e14. doi: 10.1016/j.cell.2020.10.039 33157038 PMC7590812

[59] Zhou Y, Liu Y, Gupta S, Paramo MI, Hou Y, Mao C, et al. A comprehensive SARS-CoV-2-human protein-protein interactome reveals COVID-19 pathobiology and potential host therapeutic targets. Nat Biotechnol. 2023;41(1):128–39. doi: 10.1038/s41587-022-01474-0 36217030 PMC9851973

[60] Tilocca B, Soggiu A, Sanguinetti M, Musella V, Britti D, Bonizzi L, et al. Comparative computational analysis of SARS-CoV-2 nucleocapsid protein epitopes in taxonomically related coronaviruses. Microbes Infect. 2020;22(4–5):188–94. doi: 10.1016/j.micinf.2020.04.002 32302675 PMC7156246

[61] Pryhuber GS, Huyck H. URMC_HTC_whole lung and lobe processing. 2020.

[62] Pitonzo ACA, Huyck H, Pryhuber GS. HTC_Precision_Cut_Lung_Slices V.2. 2025.

[63] Zheng GXY, Terry JM, Belgrader P, Ryvkin P, Bent ZW, Wilson R, et al. Massively parallel digital transcriptional profiling of single cells. Nat Commun. 2017;8:14049. doi: 10.1038/ncomms14049 28091601 PMC5241818

[64] Satija R, Farrell JA, Gennert D, Schier AF, Regev A. Spatial reconstruction of single-cell gene expression data. Nat Biotechnol. 2015;33(5):495–502. doi: 10.1038/nbt.3192 25867923 PMC4430369

[65] Smith T, Heger A, Sudbery I. UMI-tools: modeling sequencing errors in Unique Molecular Identifiers to improve quantification accuracy. Genome Res. 2017;27(3):491–9. doi: 10.1101/gr.209601.116 28100584 PMC5340976

[66] Langmead B, Salzberg SL. Fast gapped-read alignment with Bowtie 2. Nat Methods. 2012;9(4):357–9. doi: 10.1038/nmeth.1923 22388286 PMC3322381

[67] Xu J, Chamblee M, Hsu CC, Jiang F, Chen P, Zhang Y, et al. A rapid yeast-based reverse genetics system reveals SARS-CoV-2 Omicron BA.2.86 variant spreads faster than Omicron JN.1 variant in primary human nasal and bronchial epithelial airway cultures. mBio. 2026;17(6):e0012226. doi: 10.1128/mbio.00122-26 42112831 PMC13251420

[68] di Sant’Agnese PA, De Mesy-Jensen KL. Diagnostic electron microscopy on reembedded (“popped off”) areas of large Spurr epoxy sections. Ultrastruct Pathol. 1984;6(2–3):247–53. doi: 10.3109/01913128409018580 6380070

