# Supplementary material for: SARS-CoV-2 defective viral genomes from distinct genomic regions drive divergent interferon responses
Source: PLoS Pathog. 2026 Sep 23;22(9):e1014099. doi: 10.1371/journal.ppat.1014099 (PMC13626462; doi:10.1371/journal.ppat.1014099)
Supplement: Supplementary file 1 [file ppat.1014099.s007.docx]

**Supplementary Information**

**SI materials and methods:**

**Construction of nsp12 mutations and examination of their effects in SARS-CoV-2 mini-replicon system**

The mini-replicon system is published elsewhere[1], which contains 3 helper plasmids (ps2AN ps2AC, and ps2B) expressing all 16 nonstructural proteins to form the SARS-CoV-2 viral polymerase complex (Vp), along with a backbone (BKB) plasmid containing the 5′UTR, a firefly luciferase reporter driven by the M gene transcription regulatory sequence, and the 3′UTR. All seven mutations were introduced into the nsp12 gene of ps2B using overlap PCR. 2.5x10^5^ HEK293T cells were transfected with 20 ng Renilla luciferase plasmid (R-luc), 100 ng BKB, 25 ng ps2AN, 200 ng ps2AC, and 200 ng WT ps2B or nsp12-mutant ps2B. Dual-luciferase assays were performed 48 h post-transfection according to manufacturer’s instruction (Promega). Because Renilla luciferase is expressed under the CMV promoter it serves as a control for transfection efficiency. Viral polymerase activity was estimated by normalizing firefly luciferase activity to Renilla luciferase activity.

Reference:

1.	Luo Y, Yu F, Zhou M, Liu Y, Xia B, Zhang X, et al. Engineering a Reliable and Convenient SARS-CoV-2 Replicon System for Analysis of Viral RNA Synthesis and Screening of Antiviral Inhibitors. mBio. 2021;12(1). Epub 20210119. doi: 10.1128/mBio.02754-20. PubMed PMID: 33468688; PubMed Central PMCID: PMCPMC7845634.

**S1 Table. Oligo primer and antibody list.**

| **Primer name** | **Sequence** | **Purpose** |
| --- | --- | --- |
| Human GAPDH-F | 5’ GCAAATTCCATGGCACCGT 3’ | qPCR for host genes |
| Human GAPDH-R | 5’ TCGCCCCACTTGATTTTGG 3’ | qPCR for host genes |
| Human ISG56-F | 5’ GGATTCTGTACAATACACTAGAAACCA 3’ | qPCR for host genes |
| Human ISG56-R | 5’ CTTTTGGTTACTTTTCCCCTATCC 3’ | qPCR for host genes |
| Human IFNB1-F | 5’ GTCAGAGTGGAAATCCTAAG 3’ | qPCR for host genes |
| Human IFNB1-R | 5’ ACAGCATCTGCTGGTTGAAG 3’ | qPCR for host genes |
| Human IL29-F | 5’ CGCCTTGGAAGAGTCACTCA 3’ | qPCR for host genes |
| Human IL29-R | 5’ GAAGCCTCAGGTCCCAATTC 3’ | qPCR for host genes |
| Human ISG54-F | 5’ ATCCCCCATCGCTTATCTCT 3’ | qPCR for host genes |
| Human ISG54-R | 5’ CCACCTCAATTAATCAGGCACT 3’ | qPCR for host genes |
| Human IL-6-F | 5’ GCCCAGCTATGAACTCCTTCT 3’ | qPCR for host genes |
| Human IL-6-R | 5’ GCGGCTACATCTTTGGAATCT 3’ | qPCR for host genes |
| Human RIG-I-F | 5’ GCTGATGAAGGCATTGACATTG 3’ | qPCR for host genes |
| Human RIG-I-R | 5’ CAGCATTACTAGTCAGAAGGAAGCA 3’ | qPCR for host genes |
| Human MDA5-F | 5’ GAGGAATCAGCACGAGGAATAA 3’ | qPCR for host genes |
| Human MDA5-R | 5’ TCAGATGGTGGGCTTTGAC 3’ | qPCR for host genes |
| Spike-F | 5’ CTTGCAGATGCTGGCTTCAT 3’ | qPCR for WT SARS-CoV-2 |
| Spike-R | 5’ CTGTGAGCAAAGGTGGCAAA 3’ | qPCR for WT SARS-CoV-2 |
| N-F | 5’ GACCCCAAAATCAGCGAAAT 3’ | qPCR for WT SARS-CoV-2 |
| N-R | 5’ TCTGGTTACTGCCAGTTGAATCTG 3’ | qPCR for WT SARS-CoV-2 |
| qGFP-F | 5’ GAGCGCACCATCTTCTTCAA 3’ | qPCR for DVG-B |
| qGFP-R | 5’ TCCTTGAAGTCGATGCCCTT 3’ | qPCR for DVG-B |
| Nsp1-F | 5’ GCTTACCGCAAGGTTCTTCT 3’ | qPCR for DVG-A |
| Nsp1-mKate2-R | 5’ ATCAGCTCGCTCACCATTAAG 3’ | qPCR for DVG-A |
| DVGB-F | 5’ TGGACGAGCTGTACAAGTAA 3’ | qPCR for DVG-B |
| DVGB-R | 5’ CATTCTGCACAAGAGTAGAC 3’ | qPCR for DVG-B |
| SARS2-F1-F | 5' CAGGAAGATTGTATAAGCAAATATTTGACATTGATTATTGACTAGTTATTAATAG 3' | Generate DVG-ΔN |
| SARS2-F1-R | 5’ GCTGTAACAGCATCAGGTGAAGAAACAGAAACTGTAGCTGGCACTTTGAGAG 3’ | Generate DVG-ΔN |
| SARS2-F2-F | 5' CTCTCAAAGTGCCAGCTACAGTTTCTGTTTCTTCACCTGATGCTGTTACAGC 3' | Generate DVG-ΔN |
| SARS2-F2-R | 5’ CAAACACCAGCTTCTGATCTTTCACAAGTGCCGTGCCTACAGTACTCAGAATC 3’ | Generate DVG-ΔN |
| SARS2-F3-F | 5' GATTCTGAGTACTGTAGGCACGGCACTTGTGAAAGATCAGAAGCTGGTGTTTG 3' | Generate DVG-ΔN |
| SARS2-F3-R | 5’ GCAGAATGCATCTGTCATCCAAACAGTTAACACAATTTGGGTGGTATGTCTG 3’ | Generate DVG-ΔN |
| SARS2-F4-F | 5' CAGACATACCACCCAAATTGTGTTAACTGTTTGGATGACAGATGCATTCTGC 3' | Generate DVG-ΔN |
| SARS2-F4-R | 5’ GCTGGTGTGTGGAATGCATGTTTATTTACATACAAACTGCCACCATCACAACC 3’ | Generate DVG-ΔN |
| SARS2-F5-F | 5' GGTTGTGATGGTGGCAGTTTGTATGTAAATAAACATGCATTCCACACACCAGC 3' | Generate DVG-ΔN |
| SARS2-F5-R | 5’ CAGCAGGATCCACAAGAACAACAGCCCTTGAGACAACTACAGCAACTGG 3’ | Generate DVG-ΔN |
| SARS2-F6-F | 5' CCAGTTGCTGTAGTTGTCTCAAGGGCTGTTGTTCTTGTGGATCCTGCTG 3' | Generate DVG-ΔN |
| SARS2-F6-R | 5’ ATGCCATGCCGACCCTTTTTTTTTTTTTTTTTTTTTTTTTTTTTTTTTGTCATTCTCCTAAG 3’ | Generate DVG-ΔN |
| SARS2-F7-F | 5' CTTAGGAGAATGACAAAAAAAAAAAAAAAAAAAAAAAAAAAAAAAAAGGGTCGGCATGGCAT 3' | Generate DVG-ΔN |
| Plasmid-F7-R | 5’ CATGGAAGTGATATCGCGGAAGGAAAATACGATATGAGTCGTCTGGCGGCC 3’ | Generate DVG-ΔN |
| Plasmid-F8-F | 5' GGCCGCCAGACGACTCATATCGTATTTTCCTTCCGCGATATCACTTCCATG 3' | Generate DVG-ΔN |
| Plasmid-F8(URA3)-R | 5’ GTTATTACCCATTGAAAAAGGAAGAGTCATGTCAATACTTGCCCTTGACAGGC 3’ | Generate DVG-ΔN |
| Plasmid-F9(URA3)-7 | 5’ GCCTGTCAAGGGCAAGTATTGACATGACTCTTCCTTTTTCAATGGGTAATAAC 3’ | Generate DVG-ΔN |
| Plasmid-F9-R | 5’ CTATTAATAACTAGTCAATAATCAATGTCAAATATTTGCTTATACAATCTTCCTG 3’ | Generate DVG-ΔN |
| **Antibody name** | **Dilution used** | **Catalog (company)** |
| Anti-dsRNA, clone rJ2 | 1:2000 | MABE1134 (Millipore-Sigma) |
| Anti-SARS-CoV-2 Nucleocapsid protein | 1:2000 | 26369S (Cell Signaling Technology) |
| Anti-SARS-CoV-2 Nucleocapsid protein | 1:500 | E8R1L (Cell Signaling Technology) |
| Anti-IRF3 | 1:200 | A27272 (ABclonal Technology) |
| Anti-SARS-CoV-2 Nsp6 | 1:100 | 9177 (ProSci) |
| Anti-Strep Tag II | 1:2000 | A02342S (GenScript) |
| AlexaFluor 568 goat anti-mouse IgG2a | 1:2000 | A21134 (Invitrogen) |
| AlexaFluor 647 goat anti-rabbit IgG (H+L) | 1:2000 | A21244 (Invitrogen) |
| AlexaFluor 594 donkey anti-mouse IgG (H+L) | 1:2000 | A21203 (Invitrogen) |
| Anti-SARS-CoV-2 Spike neutralizing antibody | 10μg/mL | 40592-R001 (SinoBiological) |
| Anti-SARS-CoV-2 Spike S1-NTD antibody | 1:500 | 42172  (Cell signaling) |
| Anti-LAMP1  Rabbit monoclonal antibody | 1:500 | 9091  (Cell signaling) |
| Anti-RIG-I polyclonal antibody | 1:2000 | 25068-1-AP (proteintech) |
| Anti-MDA5 polyclonal antibosy | 1:2000 | 21775-1-AP (proteintech) |
| Anti-GAPDH monoclonal antibody | 1:5000 | ab8245  (Abcam) |
