# Supplementary material for: SARS-CoV-2 defective viral genomes from distinct genomic regions drive divergent interferon responses
Source: PLoS Pathog. 2026 Sep 23;22(9):e1014099. doi: 10.1371/journal.ppat.1014099 (PMC13626462; doi:10.1371/journal.ppat.1014099)
Supplement: Supplementary file 2 [file ppat.1014099.s008.docx]

**S2 Table. Mutations identified in DVG-B and DVG-ΔN stocks.**

|  | P2 | | P3 | | Genes | aa change |
| --- | --- | --- | --- | --- | --- | --- |
| DVG-B position | original | mutation | original | mutation |  |  |
| 308 | C (92%) | G (4%) | C (76%) | G (13%) | nsp1 |  |
| 567 | G (0%) | A (99%)* | G (0%) | A (98%)* | nsp1  (NTD) | G101D |
| 11236 | T (77%) | C (20%) | T (77%) | C (20%) |  |  |
| 11240 | T (79%) | A (20%) | T (81%) | A (18%) |  |  |
| 15182 | C (52%) | A (43%) | C (37%) | A (58%)* | nsp12 | A to D or E (4973 of pp1ab) |
| 15183 | C (50%) | G (47%) | C (34%) | G (63%)* | nsp12 | A to D or E (4973 of pp1ab) |
| 15185 | C (49%) | T (46%) | C (34%) | T (60%)* | nsp12 | T to I (4974 of pp1ab) |
| 15186 | T (48%) | C (45%) | T (27%) | C (60%)* |  | synonymous |
| 15187 | A (52%) | G (45%) | A (28%) | G (67%)* | nsp12 | R to G (4975 of pp1ab) |
| 15190 | G (60%) | A (39%) | G (32%) | A (67%)* | nsp12 | G to R (4976 of pp1ab) |
| 15195 | T (86%) | G (12%) | T (71%) | G (25%) |  | synonymous |
| 15196 | A (74%) | C (14%) | A (48%) | C (28%) | nsp12 | T to P or R (4978 of pp1ab) |
| 15197 | C (86%) | G (13%) | C (73%) | G (26%) | nsp12 | T to S (4978 of pp1ab) |
| 15203 | T (80%) | G (9%) | T (68%) | A (14%) |  |  |
| 15207 | T (81%) | C (18%) | T (77%) | C (22%) |  |  |
| 15208 | G (79%) | C (16%) | G (73%) | C (21%) |  |  |
| 15211 | A (83%) | T (13%) | A (78%) | T (17%) |  |  |
| 15213 | A (81%) | T (8%) | A (74%) | T (11%) |  |  |
| 15214 | A (85%) | T (12%) | A (79%) | T (18%) |  |  |
| 20246 | T (83%) | G (14%) | T (76%) | G (20%) |  |  |
| 23615 | C | A (100%)* | C | A (100%)* | S | R to S (685 of Spike) |
| 25312 | T | C (100%)* | T | C (100%)* | S | synonymous |
|  |  |  | **P3** | | **GENES** | **AA CHANGE** |
| DVG-ΔN position |  |  | original | mutation |  |  |
| 9996 |  |  | C (40%) | T (60%)* | nsp4 | S to L (3244 of pp1a) |
| 15182 |  |  | C (36%) | A (59%)* | nsp12 | A to D or E (4973 of pp1ab) |
| 15183 |  |  | C (35%) | G (63%)* | nsp12 | A to D or E (4973 of pp1ab) |
| 15185 |  |  | C (36%) | T (59%)* | nsp12 | T to I (4974 of pp1ab) |
| 15186 |  |  | T (34%) | C (59%)* |  | synonymous |
| 15187 |  |  | A (35%) | G (62%)* | nsp12 | R to G (4975 of pp1ab) |
| 15190 |  |  | G (38%) | A (61%)* | nsp12 | G to R (4976 of pp1ab) |
| 25721 |  |  | C (17%) | T (83%)* | orf3a | A to V (110 of orf3a) |
| 26557 |  |  | A (32%) | G (68%)* | M | E to G (12 of M) |

* highlighted all mutations with >50% of total reads aligned to that position.
