# Supplementary material for: SARS-CoV-2 defective viral genomes from distinct genomic regions drive divergent interferon responses
Source: PLoS Pathog. 2026 Sep 23;22(9):e1014099. doi: 10.1371/journal.ppat.1014099 (PMC13626462; doi:10.1371/journal.ppat.1014099)
Supplement: Supplementary file 3 [file ppat.1014099.s018.docx]

**Reviewers' Comments:**

Reviewer's Responses to Questions

**Part I - Summary**
Please use this section to discuss strengths/weaknesses of study, novelty/significance, general execution and scholarship.

Reviewer #1: This manuscript investigates the functional heterogeneity of defective viral genomes (DVGs) generated during SARS-CoV-2 infection. The authors identify two major genomic hotspots (A and B) for DVG formation and demonstrate that:

DVG abundance correlates with COVID-19 severity
DVGs from hotspot B (but not A) strongly induce type I interferon responses
Both DVG-A and DVG-B suppress wild-type virus replication
DVG-B generates dsRNA with distinct subcellular localization and enhanced immunostimulatory capacity

These findings suggest that genomic origin determines the immunological function of DVGs, providing insight into how DVGs contribute to both antiviral responses and disease severity.

Reviewer #2: In this manuscript, the authors follow-up on the analysis of Defective Viral Genomes (DVG) that were identified in a previous study. This previous study identified two dominant DVG, DVG-A and DVG-B. DVG-A was predominantly found in vitro with little/no detection in vivo.

This manuscript presents two different stories. The first story is the comparison of DVG- A and DVG-B, to define the differential role of these DVGs. However the relevance of DVG-A is not clear as detected to very low/undetectable levels in patient lung samples.

The second story is the role of DVG-B in viral infection and the mechanism of IFN induction. This aspect of the manuscript is novel and interesting given the in vivo relevance of DVG-B with detection in patient lung samples. However this aspect of the manuscript and mechanistic link to N expression is somewhat clouded by the loss of 4 viral proteins in DVG-B. The molecular linkage of N to RLR inhibition has been described before, (see critique and paper below), but there is still a novel story here. To my knowledge, this would be the first paper to demonstrate how N regulates IFN dynamics in the context of a viral infection.

Overall by combining these two stories, this manuscript is convoluted. For these reasons I would support this manuscript with a focus on just DVG-B and perform additional studies with a N deletion (or N mutant) virus. See below for more specific details.

Reviewer #3: Overall, this is a well-executed, thorough, and well-described study. The finding that DVGs with specific genetic architectures is a novel, interesting and potentially impactful finding affecting our understanding of host responses to SARS-CoV-2 as well as knowledge on how to deploy/design TIPs. Minor limitations to the study include the breath/diversity of DVG types studies. It would be interesting to characterize other specific DVGs comprising recombination junctions found the 'B' region of the DVG landscape rather than just one specific construct. But the findings of the study stand as is.

Though this is a complex and in-depth study, I have no comments or concerns with the data presented nor interpretations thereof. The studies include suitable controls and approaches that support the findings. I have only some minor comments to help on clarity of the figures/description:

In figure 2B/C, S1A and similar, it's a little unclear how cell point corresponds to a single cell with a specific DVG. Is the cells assigned according to the most abundant/prevalent DVG observed? Or, if multiple DVGs are found in cell (which seems likely), are multiple points (one for each observed DVG) plotted for a single cell?

A: We thank the reviewer for raising this point. It is the latter: multiple DVGs can be present in the same cell (sharing the same cell barcode), and therefore a single positive cell may be represented by multiple points corresponding to different DVG recombination sites. We have clarified this in the Methods section.

I found the headings on Figure 5G a little confusing. I believe this represents the ratio of IFNB1 (for example) found in WT vs DVGB cell carrying cells, but as labelled this look like a plot of the ratio of Wt genomic RNA to DVG-B RNA.

A: We agree with the reviewer and have removed the DVG-A and DVG-B labels from the figure headings and reorganized the data such that the ratios of IFNB1 or ISG54 in DVG-B versus WT are presented in Fig. 5G, while the corresponding ratios for DVG-A versus WT are shown in Fig. 5H.

The separation of the Wt sample data point in the PCA analysis in Figure S2A is concerning and may reduce the accuracy/power of the DE analyses presented in figure 5 and related. Is the dispersion/variability of these Wt data when using all 4 replicates higher than the other samples in this analysis? Or rather, are a small number of genes driving this separation (e.g. some cells might be in a later stage of infection and proceeding to CPE/death). Ideally, if feasible, these RNAseq libraries should be repeated. But since the Wt point do not co-cluster with other data points and that the findings are generally robust and consistent with expectations, this is only of minor concern and would be a 'good-to-have' rather than a core issue.

A: We thank the reviewer for raising this point. To address the concern, we performed deep sequencing on three additional WT virus–infected samples (24hpi) under the same conditions. Combined with the original four WT samples, PCA analysis revealed that two earlier replicates (repeat 1 and repeat 2) were outliers compared to the rest of the WT samples (panel A, below). We therefore excluded these two samples and included the three new replicates to reduce within-group variability, and repeated the downstream analyses (updated PCA shown in panel B, also included in new Fig. S2A). To further minimize batch effects, we applied additional correction using the duplicateCorrelation function in the limma package. Using this refined dataset, we identified approximately 1,800 differentially expressed genes (DEGs) across mock, DVG-B, WT, and WT+DVG-B conditions. Of these, ~1,700 DEGs were upregulated in WT alone, while ~100 were upregulated in DVG-B alone. Compared to our previous analysis, the total number of DEGs decreased substantially, likely reflecting reduced within-group variation. Despite this reduction, the enriched pathways associated with WT and DVG-B upregulated genes remained consistent with our prior findings (new Fig. 5A-C). Further, consistent with previous results, very few genes were significantly downregulated in WT and DVG-B, and no pathways showed significant enrichment. The conclusions for DVG-A also remained unchanged (Fig. S2B). We have updated the heatmaps, GO analyses, and DEG lists in new Fig. 5, Fig. S2, and Table S5-S9, and revised the reported DEG numbers accordingly in the results.

**Part II – Major Issues: Key Experiments Required for Acceptance**
Please use this section to detail the key new experiments or modifications of existing experiments that should be absolutely required to validate study conclusions.

Generally, there should be no more than 3 such required experiments or major modifications for a "Major Revision" recommendation. If more than 3 experiments are necessary to validate the study conclusions, then you are encouraged to recommend "Reject".

Reviewer #1: (No Response)

Reviewer #2: Comparison of DVG-A vs DVG-B is a bit contrived. Method of synthesis of these two DVGs are very different and the level of RNA resulting from these will be massively different inside the host cell as DVG-B is replication competent and DVG-A is not. Also DVG-A appears largely to be an artifact of tissue culture as there is little/no detection in lung patient samples. Recommend to cut DVG-A comparison from the manuscript.

A: We agree with the reviewer that the preparation methods for DVG-A and DVG-B differ. DVG-A is a relatively minor species in patients compared to DVG-B, and its abundance during infection is substantially lower, suggesting that it might play a limited role in viral pathogenesis in the patient cohort we studied. Our intention was not to directly compare their antiviral potency or conclude that they have equivalent roles in SARS-CoV-2 pathogenesis. Rather, we included DVG-A because we believe it provides important translational insights that complement our characterization of DVG-B. Firstly, DVG-A is one of the major hotspots in vitro and we do not consider DVG-A to be an artifact of tissue culture. DVG-A can be consistently detected across multiple in vitro infection systems, including primary human bronchial epithelial cells and different variants of concern. They can be detected in some DVG+ patients with low abundance. They are predominantly detected in infected cells rather than in supernatants. All these observations suggest that while DVGs in hotspot A are readily generated, they may be less efficient in packaging. Indeed, natural DVG-A lacks PS9 sequence, one reported packaging signal for SARS-CoV-2. This could result in less efficient dissemination and consequently low abundance in clinical samples. Secondly, despite DVG-A’s low abundance in patient samples, it has great therapeutic potential. Our data show that DVG-A particles can suppress WT viral infection when administered concurrently and, notably, do not elicit strong interferon responses at the current level of infection. These data indicate that DVG-A suppresses WT virus replication through an interferon-independent mechanism that is mechanistically distinct from DVG-B. Consistent with this concept, synthetic SARS-CoV-2 RNAs with similar genomic structures have previously been developed as therapeutic interfering particles (TIPs) and shown to provide potent antiviral protection in the golden hamster model [1]. Thus, despite its lower abundance relative to DVG-B, inclusion of DVG-A in this study provides valuable insight into its translational applications and underscores the need for further studies to elucidate the mechanisms by which it suppresses WT virus. We have incorporated the above information into the revised Discussion (Line 648-667) to emphasize the translational, rather than pathogenic, significance of DVG-A.

Authors attempt to ascribe the majority of IFN induction to the loss of N. This is supported by the rescue data and the previous description of N function (see above). However the conclusions with DVG-B are somewhat clouded as there is a loss of Orf7a/7b, Orf8, and N in DVG-B. To more directly assess the role of N in DVG-B gene regulation, the authors should create a KO virus with just N deleted (or active site mutations) but retaining expression of Orf7a/7b and Orf8. A complete repeat of all studies with the new virus is not necessary, but the authors should perform studies to validate their findings and link the IFN induction specifically to a loss of N.

A: We agree that our original writing implied that the absence of the nucleocapsid protein was responsible for DVG-B’s interferon induction without explicitly demonstrating this point. We have since generated new Figure 9, Figure S5, and the corresponding text in the results section (Lines 467-500).

“DVG-B lacks four proteins in addition to the absence of the N protein. To assess whether the lack of orf7a, orf7b, or orf8 contribute to DVG-B’s interferon induction activity or its dsRNA distribution profile, we generated SARS-CoV-2 virus-like particles packaging a recombinant SARS-CoV-2 lacking the N open reading frame but retaining orf7a, orf7b and orf8 (DVG-ΔN, Fig. 9A). DVG-ΔN was passaged similarly as DVG-B in VeroE6:N cells. Deep sequencing of DVG-ΔN P3 stocks exhibited the introduced deletion when aligned to the WT viral reference genome (Fig. S5A), whereas this deletion was not observed in the alignment using DVG-ΔN as the reference genome (Fig. S5B). Additionally, DVG-ΔN showed comparable levels and similar distributions of additional deletions as DVG-B P3 (Fig. S5C vs Fig. S3C). Interestingly, the nsp12 point mutations observed in the DVG-B stock were again observed in the DVG-ΔN stock (Table S2). To compare the IFN-induction activity of DVG-ΔN and DVG-B, A549-ACE2 cells were infected with WT, DVG-B, or DVG-ΔN (MOI 1) for 24 h. RT-qPCR showed that both DVG-B and DVG-ΔN, but not WT virus, induced similar level of IFNB1, IL-29, and ISG54 expression (Fig. 9B). Consistently, bulk RNA-seq revealed highly similar host transcriptional responses between DVG-B and DVG-ΔN infected cells, with only 29 differentially expressed genes identified (PCA analysis showed that DVG-B and DVG-ΔN clustered together in Fig. S5E and heatmap shown in Fig. S5F). Confocal microscopy further showed a similar pattern of discrete dsRNA distribution in DVG-B- and DVG-ΔN-infected cells (Fig. 9C), indicating that deletion of the N open reading frame largely recapitulates the IFN induction and dsRNA distribution observed with DVG-B. It should be noted that because orf9b is translated from an alternative start codon in N encoding mRNA, as a consequence of leaky ribosomal scanning, DVG-B and DVG-ΔN also lack orf9b[2]. Orf9b reportedly interacts with the Translocase of the Outer Mitochondrial Membrane protein 70 (TOM70) to suppress RLR mediated IFN induction[3, 4]. Thus, we sought to determine whether the complementation of orf9b impacts IFN induction during DVG-ΔN infection. A549-ACE2 cells were transduced with lentiviral vectors encoding SARS-CoV-2 orf9b or an empty lentiviral vector (EV) control for 48-hours (9b expression shown in Fig. S5G) followed by DVG-ΔN infection. 24-hours post DVG-ΔN infection, orf9b complementation yielded a slight reduction in IFNB1 and IL-29 expression, while no differences in ISG54 expression were observed compared to EV transduced cells (Fig. 9D). Overall, our data support the conclusion that while the absence of orf9b modestly contributes to DVG-B’s IFN induction activities, the loss of the N protein, rather than orf7a, orf7b, or orf8, is primarily responsible for DVG-B’s IFN induction activity and its distinct dsRNA distribution.”

Reviewer #3: (No Response)

**Part III – Minor Issues: Editorial and Data Presentation Modifications**
Please use this section for editorial suggestions as well as relatively minor modifications of existing data that would enhance clarity.

Reviewer #1: 1. Mechanistic depth: What makes DVG-B more immunostimulatory?
The central conclusion is that DVG-B induces stronger IFN responses, but the mechanism remains somewhat descriptive.
• The authors show distinct dsRNA localization and IRF3 activation
• However, it is unclear which sensor(s) are responsible (e.g., MDA5 vs RIG-I)
Suggestion:
• Include or discuss:
o MAVS dependency (KO or inhibition)
o MDA5 vs RIG-I contribution
• Even discussion-level clarification would strengthen mechanistic insight

A: Thank you for this comment. We agree that while we demonstrated that DVG-B’s IFN induction is MAVS dependent (Figure 6H), the respective contributions of MDA5 and RIG-I to DVG-B’s IFN induction are key mechanistic questions. To address this, we have since added Figure 6I and 6J and the corresponding lines in the results section (Lines 328-338).

“To determine which RLR sensor is responsible for recognizing DVG-B-derived dsRNA, we transfected A549-ACE2 cells with siRNAs targeting either RIG-I or MDA5, followed by DVG-B infection. Interestingly, although both sensors contributed to the antiviral response, RIG-I knockdown markedly reduced DVG-B-induced IFNB1 expression (Fig. 6I–J), demonstrating that RIG-I is the primary sensor detecting DVG-B-derived dsRNA. Notably, MDA5 mRNA levels were also reduced in RIG-I-knockdown cells following DVG-B infection, likely as a secondary consequence of the marked reduction in IFNB1 induction compared with scrambled siRNA-treated cells. Additionally, under mock-infected conditions, only the targeted gene was reduced, indicating that the siRNA knockdown was specific (Fig. 6I).”

2. DVG abundance vs disease severity – causation vs correlation
The manuscript reports that DVG levels correlate with COVID-19 severity
However:
• Severe disease may simply reflect higher viral replication → more DVGs
Suggestion:
• Clarify this limitation explicitly in Discussion
• If possible:
o Normalize DVGs to viral load (Jfreq is mentioned but not emphasized enough)
• A short paragraph would greatly improve interpretation rigor

A: We agree with the reviewer and incorporated the following sentences in discussion (Line 617-620).

“DVG+ patients also exhibited significantly higher viral loads than DVG- patients, suggesting that these DVGs likely arise as a consequence of high WT virus replication. In other words, in DVG+ elderly individuals with severe diseases, higher levels of viral replication may lead to increased production of DVGs.”

3. Physiological relevance of engineered DVGs
The authors construct representative DVG-A and DVG-B and test them functionally.
Concern:
• Do these constructs fully recapitulate natural DVG diversity?
Suggestion:
• Clarify:
o How representative these constructs are of endogenous DVG populations
• Possibly include:
o Sequence variability or structural predictions

A: We thank the reviewer for raising this important point. In this study, we generated representative DVGs using the outer boundaries of each hotspot to capture the largest viral protein deletions within each hotspot. Unfortunately, due to the high diversity of DVG junctions within each hotspot, systematically evaluating the sequence variability or predicting the RNA structures of every individual DVG species would require extensive additional work beyond the scope of the current study. We therefore have included the below paragraph in discussion to point out this limitation in this study (Line629-647).

“In our previous study, we identified two major genomic regions—hotspot A and hotspot B—where DVG recombination sites cluster. Hotspot A is defined by a rejoin point (E’) ranging from 700 to 2500 and a break site (V’) from 28,200 to 29,750, whereas hotspot B includes a rejoin point from 27,700 to 29,400 with similar break sites. To capture the full range of DVGs within these two hotspots, we used the outer boundaries of these respective recombination junctions to generate representative DVGs, DVG-A (700–29,750) and DVG-B (27,700–29,750). To ensure efficient replication and packaging for downstream functional analysis, we retained a portion of the orf10 sequence (thereby changing the 5′ end to 29,674) and incorporated a PS9 packaging signal in DVG-A. Although our synthetic DVGs cannot represent the extensive diversity of DVG junctions generated during natural infection, we anticipate that they recapitulate the key functional properties of their respective hotspots. In particular, DVGs derived from hotspot B are expected to similarly induce IFN responses if N expression is absent. This is further supported by the similar IFN and dsRNA distribution between DVG-ΔN and DVG-B. Nonetheless, further validation using authentic DVGs is warranted. Future studies, such as enriching DVGs in WT virus stock through high-MOI passaging, constructing other DVGs with different deletions identified from natural infection will help address this point.”

4. Role of nucleocapsid (N) protein—interpretation needs refinement
The authors show:
• N complementation changes dsRNA localization
• But does not change IFN response
This is interesting but under-discussed.
❗ Key question:
Suggestion:
• Expand discussion:
o Is spatial organization uncoupled from sensing?
o Or is sensing already saturated?
This could be a conceptually important point

A: Thank you for this thoughtful comment. To address these suggestions, we have added the following in the discussion section (Line550-569).

“First, is RLR signaling saturated in our system? We disfavor this hypothesis given that N complementation enhanced, rather than maintained, DVG-B-induced IFN responses, indicating that there is a sufficient amount of RLRs available to detect additional immunostimulatory RNA species. Second, is dsRNA spatial organization uncoupled from RLR sensing? The rJ2 antibody used in this study recognizes a minimum dsRNA epitope of ~40 bp and therefore cannot reliably detect shorter dsRNA species. In addition, because SARS-CoV-2 generates dsRNAs that can reach several kilobases in length, our microscopy analyses are inherently biased toward detecting these long dsRNA species. Our RIG-I and MDA5 knockdown data suggest that RIG-I is the major sensor for DVG-B-derived dsRNA (Fig. 6I). Given that RIG-I preferentially recognizes short blunt-ended dsRNAs with a 5′-triphosphate (5′ppp) or 5′-diphosphate (5′pp) end (typically 10bp-300bp)[5, 6], our immunofluorescent images cannot fully reflect the cellular distribution of these shorter dsRNAs. While we consistently observed that N protein complementation redistributed the pattern of, presumably long, dsRNA species during DVG-B infection, we suspect that the N protein also drove greater production of other IFN inducing RNA species generated by DVG-B, such as shorter dsRNA species. These shorter dsRNAs could be degraded products of viral genomes when not properly coated with N and exporting outside of DMVs, driving higher IFN induction. Therefore, we hypothesize that the sensing of long dsRNAs, perhaps by MDA5, is coupled to their spatial organization, whereas RIG-I activation is independent of the spatial distribution of these long dsRNA species. Further studies are required to determine the DVG-B derived RNA species activating RIG-I and how N protein complementation impacts its abundance and localization.”

5. Replication vs immune activation trade-off
Both DVG-A and DVG-B suppress WT virus replication, but only DVG-B induces strong IFN.
Suggestion:
• Discuss:
o Are these two separable functions?
o Could DVG-A act as a “silent suppressor”?

A: We thank the reviewers for raising these two important points. We have incorporated the following paragraph in the discussion (Line522-540).

“The distinct capacities of DVG-A and DVG-B to induce IFN suggest that these two DVGs suppress WT virus through different mechanisms. Because DVG-B strongly induces IFN responses, its robust IFN induction likely contributes to the suppression of WT virus replication. In addition, although DVG-B can express nonstructural proteins, it lacks the essential assembly component N. Therefore, DVG-B may compete with WT viral genomes for assembly machinery, thereby reducing overall WT virus production during coinfection. Further investigation of WT virus suppression by DVG-B in IFN-deficient cells, such as Vero E6 cells, will be needed to determine the relative contributions of IFN-dependent and IFN-independent mechanisms of DVG-B in suppressing WT virus replication. For DVG-A, our data clearly indicate that it suppresses WT virus through an IFN-independent mechanism. Consistent with this observation, a previous study demonstrated that a synthetic DVG with a genomic structure similar to DVG-A can be developed into a therapeutic interfering particle (TIP) that effectively suppresses wild-type SARS-CoV-2 infection in Syrian golden hamsters without inducing IFN responses[7]. The molecular mechanism by which DVG-A inhibits WT virus replication remains unknown. Because DVG-A is relatively short and retains both the 5′ and 3′ UTRs, it may compete with WT viral genomes for replication and assembly. DVG-A particles may, alternatively, compete with SARS-CoV-2 for ACE2 at entry. In addition, the DVG-A genome may form unique secondary structures and generate distinct small interfering RNAs (siRNAs) that could further target WT viral genomes.”

Minor Comments
Clarity & Writing
• Some sections of the Introduction are slightly long—could be streamlined
• Define “hotspot A/B” earlier for clarity

A: We have now clarified the boundary hotspot A/B in the introduction.

Figures
• Figure legends are generally clear, but:
o Add schematic for DVG-A vs DVG-B structure (very helpful visually)
• In imaging data:
o Quantification should always accompany representative images (some already do well)

A: Thank you for this comment, we have since added schematics of the DVG-B and DVG-A genomes into Figure 3A. Additionally, for each figure containing representative widefield or confocal images, we have added the number of fields imaged, or the number of spike containing structures quantified. Regarding the quantification of our electron micrographs, we are of the opinion that our TEM identification/classification of sub-cellular structures is inherently qualitative because we did not employ any method specifically labelling lysosomes. Therefore, our characterization of “lysosome-like structures” is based on descriptions consistent with our images. Therefore, to validate our TEM findings of impaired DVG-B assembly and lysosome-like structures in DVG-B-infected cells, we performed additional immunofluorescence staining using antibodies against SARS-CoV-2 spike and the lysosomal marker LAMP-1. These new data have been included in the new Figure 8, and the corresponding text has been added to the revised manuscript (Line 450-466).

“Because our TEM methods yielded results that were inherently qualitative, we sought to confirm the identity of the tertiary lysosome-like structures present in DVG-B infected cells via immunofluorescence assays. Given the inverse relationship in the abundance of SMVs and tertiary-lysosome like structures in DVG-B infected cells, we used spike and LAMP-1 to mark SMVs and lysosomes, respectively. At 24 hpi, WT virus derived spike protein was observed widely distributed throughout the cell, likely marking the ER, ERGIC, exocytic vesicles and the plasma membrane. DVG-B derived spike protein, however, was less abundant overall and localized to circular hollow structures additionally containing LAMP1 (Fig. 8B). Line intensity analysis showed that spike and LAMP1 strongly correlated with one another in these structures (Fig. 8B, left). While N protein complementation did not impact spike distribution in WT virus infected cells, among DVG-B infected cells we observed that N largely restored the spike protein distribution to that of SARS-CoV-2. Notably, N protein complementation yielded a loss of these spike/LAMP1 containing-lysosomal structures during DVG-B infection. Furthermore, we determined that DVG-B derived dsRNA localizes to a distinct compartment relative to spike containing lysosomes (Fig. 8C). Overall, our data indicate that absence of the N protein drastically altered the cellular distribution of the spike protein and further suggest that the N protein is required for optimal SMV maintenance.”

Further, we have added the following lines of text to the discussion (Lines 589-605):

“Our data additionally highlight a critical role of N in viral structural protein trafficking and virus particle assembly. The large lysosome-like structures observed in DVG-B–infected cells are intriguing. The co-staining of spike and LAMP-1 revealed that DVG-B infection resulted in a markedly altered spike distribution pattern, characterized by hollow spike-containing structures associated with LAMP-1 staining. Given the striking reduction in SMV abundance, and the corresponding increase of these lysosomal-structures, in DVG-B infected cells compared to WT, we speculate that these structures form as a consequence of aberrant SMV formation or maintenance. Previous studies have reported that β-coronaviruses utilize a lysosomal Arl8b-dependent exocytic pathway for virus egress [8]. Additionally, SARS-CoV-2 protein-human protein interactome data has previously identified the post-ER trafficking proteins: GOLGA8F, GIPC2, and ARPC3 as N interactors[9]. It is therefore possible that the N protein plays a role in maintaining the stability of the SMV or regulating post-ERGIC pathways involved in SARS-CoV-2 virus particle trafficking. Consequently, loss of the N protein may ultimately result in localization of the SMV to the lysosome. To better assess how the N protein, or its lack thereof, impacts SMV formation and maintenance, future experiments may seek to evaluate their morphology at various time points post-infection and assess whether the viral envelope and membrane proteins also localize to the lysosome.”

Methods
• Clarify:
o MOI consistency across experiments
o Whether IFN readouts are normalized per infected cell
Data Availability
• GEO deposition is “in progress”

A: RNA sequencing data is available at GSE337138. All MOIs have been indicated in the figure legends. Note that lower MOI was used in WT infection in N expressing cells due to higher cell death noticed in WT-infected transduced cells.

Reviewer #2: The role of viral nucleocapsid protein has previously been described. Aloise et al 2023. [https://doi.org/10.1371/journal.ppat.1011582](https://urldefense.com/v3/__https:/doi.org/10.1371/journal.ppat.1011582__;!!MkQhnT5yHH0MPvOc00c!WimnaqqovEfZ_vQXf2mJE3rTjcksCm2Q3m49OEEep_az9uabFh5x-h6S7ZEdwwGwzLgxDPhRHp2hQ3ZFkyLs92Um$). Specifically this previous publication demonstrated that N inhibits RLR pathway. This paper is not referenced or discussed at all, but could explain nearly all the phenotypes associated with DVG-B. Authors should put findings in context of this previous publication.

A: Thank you for bringing this citation to our attention. We have added the above citation and the following paragraph discussing our findings in the context of previous descriptions of the N protein’s role in interferon antagonism (Lines 577-588):

“DVG-B and DVG-ΔN induced comparable levels of IFN, strongly suggesting that their IFN-inducing activity is primarily attributable to the absence of the N protein. N protein antagonism of RLR pathway has been previously described[10-13]. Interestingly, the level of N expression is also reported to impact N-mediated antagonism of IFN induction. While low doses of N suppress IFN and ISG induction, high doses of N promote their expression[14]. Consequently, it is possible that the N protein level in our A549:N cells inherently promote IFN induction during DVG-B infection, independent of enhanced viral RNA synthesis activity. Alternatively, it is possible that the kinetics of N protein production, and its accumulation over time, are important for its antagonism of IFN induction. To our knowledge, this is the first study demonstrating that the absence of N enhances IFN induction in the context of SARS-CoV-2 infection.”

**References:**

1.	Chaturvedi S, Vasen G, Pablo M, Chen X, Beutler N, Kumar A, et al. Identification of a therapeutic interfering particle-A single-dose SARS-CoV-2 antiviral intervention with a high barrier to resistance. Cell. 2021;184(25):6022-36.e18. Epub 2021/11/29. doi: 10.1016/j.cell.2021.11.004. PubMed PMID: 34838159; PubMed Central PMCID: PMCPMC8577993.

2.	Xu K, Zheng BJ, Zeng R, Lu W, Lin YP, Xue L, et al. Severe acute respiratory syndrome coronavirus accessory protein 9b is a virion-associated protein. Virology. 2009;388(2):279-85. Epub 20090425. doi: 10.1016/j.virol.2009.03.032. PubMed PMID: 19394665; PubMed Central PMCID: PMCPMC7103405.

3.	Gao X, Zhu K, Qin B, Olieric V, Wang M, Cui S. Crystal structure of SARS-CoV-2 Orf9b in complex with human TOM70 suggests unusual virus-host interactions. Nat Commun. 2021;12(1):2843. Epub 20210514. doi: 10.1038/s41467-021-23118-8. PubMed PMID: 33990585; PubMed Central PMCID: PMCPMC8121815.

4.	Jiang HW, Zhang HN, Meng QF, Xie J, Li Y, Chen H, et al. SARS-CoV-2 Orf9b suppresses type I interferon responses by targeting TOM70. Cell Mol Immunol. 2020;17(9):998-1000. Epub 20200729. doi: 10.1038/s41423-020-0514-8. PubMed PMID: 32728199; PubMed Central PMCID: PMCPMC7387808.

5.	Pichlmair A, Schulz O, Tan CP, Naslund TI, Liljestrom P, Weber F, et al. RIG-I-mediated antiviral responses to single-stranded RNA bearing 5'-phosphates. Science. 2006;314(5801):997-1001. Epub 20061012. doi: 10.1126/science.1132998. PubMed PMID: 17038589.

6.	Schlee M, Roth A, Hornung V, Hagmann CA, Wimmenauer V, Barchet W, et al. Recognition of 5' triphosphate by RIG-I helicase requires short blunt double-stranded RNA as contained in panhandle of negative-strand virus. Immunity. 2009;31(1):25-34. Epub 20090702. doi: 10.1016/j.immuni.2009.05.008. PubMed PMID: 19576794; PubMed Central PMCID: PMCPMC2824854.

7.	Chaturvedi S, Vasen G, Pablo M, Chen X, Beutler N, Kumar A, et al. Identification of a therapeutic interfering particle-A single-dose SARS-CoV-2 antiviral intervention with a high barrier to resistance. Cell. 2021;184(25):6022-36 e18. Epub 2021/11/29. doi: 10.1016/j.cell.2021.11.004. PubMed PMID: 34838159; PubMed Central PMCID: PMCPMC8577993.

8.	Ghosh S, Dellibovi-Ragheb TA, Kerviel A, Pak E, Qiu Q, Fisher M, et al. beta-Coronaviruses Use Lysosomes for Egress Instead of the Biosynthetic Secretory Pathway. Cell. 2020;183(6):1520-35 e14. Epub 20201027. doi: 10.1016/j.cell.2020.10.039. PubMed PMID: 33157038; PubMed Central PMCID: PMCPMC7590812.

9.	Zhou Y, Liu Y, Gupta S, Paramo MI, Hou Y, Mao C, et al. A comprehensive SARS-CoV-2-human protein-protein interactome reveals COVID-19 pathobiology and potential host therapeutic targets. Nat Biotechnol. 2023;41(1):128-39. Epub 20221010. doi: 10.1038/s41587-022-01474-0. PubMed PMID: 36217030; PubMed Central PMCID: PMCPMC9851973.

10.	Aloise C, Schipper JG, van Vliet A, Oymans J, Donselaar T, Hurdiss DL, et al. SARS-CoV-2 nucleocapsid protein inhibits the PKR-mediated integrated stress response through RNA-binding domain N2b. PLoS Pathog. 2023;19(8):e1011582. Epub 20230822. doi: 10.1371/journal.ppat.1011582. PubMed PMID: 37607209; PubMed Central PMCID: PMCPMC10473545.

11.	Gori Savellini G, Anichini G, Gandolfo C, Cusi MG. SARS-CoV-2 N Protein Targets TRIM25-Mediated RIG-I Activation to Suppress Innate Immunity. Viruses. 2021;13(8). Epub 20210723. doi: 10.3390/v13081439. PubMed PMID: 34452305; PubMed Central PMCID: PMCPMC8402637.

12.	Oh SJ, Shin OS. SARS-CoV-2 Nucleocapsid Protein Targets RIG-I-Like Receptor Pathways to Inhibit the Induction of Interferon Response. Cells. 2021;10(3). Epub 20210302. doi: 10.3390/cells10030530. PubMed PMID: 33801464; PubMed Central PMCID: PMCPMC7999926.

13.	Chen K, Xiao F, Hu D, Ge W, Tian M, Wang W, et al. SARS-CoV-2 Nucleocapsid Protein Interacts with RIG-I and Represses RIG-Mediated IFN-beta Production. Viruses. 2020;13(1). Epub 20201230. doi: 10.3390/v13010047. PubMed PMID: 33396605; PubMed Central PMCID: PMCPMC7823417.

14.	Zhao Y, Sui L, Wu P, Wang W, Wang Z, Yu Y, et al. A dual-role of SARS-CoV-2 nucleocapsid protein in regulating innate immune response. Signal Transduct Target Ther. 2021;6(1):331. Epub 20210901. doi: 10.1038/s41392-021-00742-w. PubMed PMID: 34471099; PubMed Central PMCID: PMCPMC8409078.
