# Supplementary material for: SARS-CoV-2 defective viral genomes from distinct genomic regions drive divergent interferon responses
Source: PLoS Pathog. 2026 Sep 23;22(9):e1014099. doi: 10.1371/journal.ppat.1014099 (PMC13626462; doi:10.1371/journal.ppat.1014099)
Supplement: Supplementary file 4 [file ppat.1014099.s019.docx]

Reviewer #1: Clarify the rationale for selecting representative DVG-A and DVG-B constructs. Although this point is discussed in the Discussion, it would be helpful to briefly explain in the Methods or Results why the outermost deletion boundaries were chosen and how well these constructs represent the diversity of naturally occurring DVGs.

A: Thank you for this comment. We have since added the following to the results and methods sections, respectively.

Lines 217-219 in the result section: “To capture a broad diversity of DVG recombination junctions and the cumulative functional consequences of deletions arising within these hotspots, we selected the outer boundaries of these respective recombination junctions to construct DVG-A and DVG-B.”

Lines 783-787 in the method section: “Representative DVG-A and DVG-B constructs were designed using the outermost deletion boundaries identified within hotspot A and hotspot B, respectively. The outermost boundaries were selected to capture a diversity of DVG recombination junctions thereby preserving the cumulative functional consequences associated with diverse sequence deletion in each DVG class.”

Temper statements implying causality between DVG abundance and disease severity. The patient data demonstrate a positive association, but because DVG abundance also correlates with viral load, the manuscript should consistently emphasize that these observations are correlative rather than evidence that DVGs contribute directly to disease severity.

A: Thank you for this comment. We agree that it is essential to emphasize that the relationship between DVG abundance and disease severity may be purely correlative. We have since added the following in the results and discussion sections:

Lines 174-176 in the results: “It is important to note, however, that because DVG abundance positively correlated with viral load, the relationship between DVG abundance and disease severity may be purely correlative rather than evidence that DVGs directly contribute to disease severity.”

Lines 641-643 in the discussion: “Consequently, it is important to emphasize that the association between DVG abundance and disease severity may be strictly correlative and does not necessarily constitute evidence that DVGs directly contribute to disease severity.”

Clarify the statistical analysis for single-cell RNA-seq data. The manuscript would benefit from a clearer description of how cells containing multiple DVG junctions were handled and whether statistical comparisons accounted for repeated observations from the same cell.

A: Thanks for the reviewer’s comments. Cells containing multiple DVG junctions are represented as separate dots and were therefore counted multiple times in the comparison, regardless of whether the cells were positive for DVG-A or DVG-B. This information was previously included in the Materials and Methods section, and we have clarified the description further in the revised manuscript (Lines 756-757).

Expand the discussion of RIG-I versus MDA5 recognition. While the new knockdown experiments support a predominant role for RIG-I, the possibility that MDA5 contributes at later stages of infection or recognizes different DVG-derived RNA species should be discussed more explicitly.

A: Thank you for this comment. We agree that this manuscript would benefit from a discussion exploring the RNA species that may be activating RIG-I and/or MDA5 and how the time point chosen for experimentation may not fully reflect the contributions of these sensors at different stages of infection. We have since restructured and expanded the RIG-I and MDA5 discussion:

Lines 568-590: “MDA5 reportedly detects long dsRNAs (replication intermediates) generated during SARS-CoV-2 genomic replication[21, 22, 50]. However, our RIG-I and MDA5 knockdown data suggest that RIG-I is the major sensor for detecting DVG-B-derived dsRNA (Fig. 6I). RIG-I preferentially recognizes short blunt-ended dsRNAs with a 5′-triphosphate or 5′-diphosphate, typically 10-300 bp in length [51, 52]. Our data suggest that such short dsRNAs are the major IFN-inducing stimuli during DVG-B infection. Given the absence of N, RIG-I may detect short dsRNA species formed as a consequence of genomic RNA degradation following its export from the DMV. Like SARS-CoV-2, DVG-B’s long dsRNA replication intermediates may be detected by MDA5, whose access to such dsRNAs may be enhanced in the absence of N. While we consistently observed that N protein complementation redistributed the pattern of long dsRNA species detected by the rJ2 antibody during DVG-B infection, we suspect that the N protein also drove greater production of shorter dsRNA species, detected by RIG-I to further elevate IFN responses. Therefore, we hypothesize that the sensing of long dsRNAs, perhaps by MDA5, is coupled to their spatial organization. In contrast, RIG-I activation may depend on the abundance of short dsRNAs and be independent of the spatial distribution of long dsRNA species. Additionally, because our analyses assessed IFN induction at 24 hpi, it is possible that the relative contributions of RIG-I and MDA5 differ at different stages of viral infection. For example, at time points greater than 24 hpi, as dsRNA genomic replication intermediates accumulate, MDA5 may play a relatively larger role in IFN induction. Future studies are necessary to identify the specific DVG-B derived dsRNA species responsible for activating RIG-I and/or MDA5, determine how N protein complementation affects their abundance and localization, and clarify the relative contributions of RIG-I and MDA5 to IFN induction throughout the course of infection in the presence and absence of N.”

Improve figure readability. Several multi-panel figures (particularly Figures 5–9) contain numerous panels and small font sizes. Increasing font size and simplifying panel labels would improve readability.

A: To improve readability of Fig.5-9, we simplified some labels and increase font size of all labels to 7 or 8 pt. We also noticed an inconsistency in the labeling of IFNL1 in Figs. 2 and S1. Since it was labeled as IL-29 elsewhere, we changed IFNL1 to IL-29 in these two figures for consistency.

Minor editorial corrections. A careful proofreading is recommended to correct several grammatical errors and improve wording consistency (e.g., "Interesting" instead of "Interestingly" in the Results, consistent formatting of IFN-β/IFNB1, and uniform use of "hotspot A" and "hotspot B" throughout the manuscript).

A: Thank you for this comment. We have since carefully assessed the document to correct grammatical errors and to ensure consistent formatting in our writing and in our figures.
