# Supplementary material for: SARS-CoV-2 defective viral genomes from distinct genomic regions drive divergent interferon responses
Source: PLoS Pathog. 2026 Sep 23;22(9):e1014099. doi: 10.1371/journal.ppat.1014099 (PMC13626462; doi:10.1371/journal.ppat.1014099)
Supplement: Peer review history

# Peer review: Decision Letter 0

**DOI:** 10.1371/journal.ppat.1014099.r001

**Authors:** Holly Ramage, Michael Letko

23 Apr 2026

PPATHOGENS-D-26-00695

SARS-CoV-2 Defective Viral Genomes from Distinct Genomic Regions Drive Divergent Interferon Responses

PLOS Pathogens

Dear Dr. Sun,

Thank you for submitting your manuscript to PLOS Pathogens. After careful consideration, we feel that it has merit but does not fully meet PLOS Pathogens's publication criteria as it currently stands. Therefore, we invite you to submit a revised version of the manuscript that addresses the points raised during the review process. In particular, the reviewers would like additional mechanistic insight into the distinct responses elicited by different DVGs and how this may impact infection. Further, the relationship between the subcellular localization of dsRNA and IFN production should be more thoroughly addressed. While you did not find a role for the N protein in modulating IFN, this function has been suggested in previous publications and a discussion on this point should be included.

Please submit your revised manuscript by Jun 22 2026 11:59PM. If you will need more time than this to complete your revisions, please reply to this message or contact the journal office at plospathogens@plos.org. When you're ready to submit your revision, log on to https://www.editorialmanager.com/ppathogens/ and select the 'Submissions Needing Revision' folder to locate your manuscript file.

Please include the following items when submitting your revised manuscript:

* A letter that responds to each point raised by the editor and reviewer(s). You should upload this letter as a separate file labeled 'Response to Reviewers'. This file does not need to include responses to any formatting updates and technical items listed in the 'Journal Requirements' section below.

* A marked-up copy of your manuscript that highlights changes made to the original version. You should upload this as a separate file labeled 'Revised Manuscript with Track Changes'.

* An unmarked version of your revised paper without tracked changes. You should upload this as a separate file labeled 'Manuscript'.

If you would like to make changes to your financial disclosure, competing interests statement, or data availability statement, please make these updates within the submission form at the time of resubmission. Guidelines for resubmitting your figure files are available below the reviewer comments at the end of this letter.

We look forward to receiving your revised manuscript.

Kind regards,

Holly Ramage

Academic Editor

PLOS Pathogens

Michael Letko

Section Editor

PLOS Pathogens

Sumita Bhaduri-McIntosh

Editor-in-Chief

PLOS Pathogens

orcid.org/0000-0003-2946-9497

Michael Malim

Editor-in-Chief

PLOS Pathogens

orcid.org/0000-0002-7699-2064

**Journal Requirements:**

1) Please confirm whether your study includes live participants, human specimens or data. If so, please insert an Ethics Statement at the beginning of your Methods section, under a subheading 'Ethics Statement'. It must include:

i) The full name(s) of the Institutional Review Board(s) or Ethics Committee(s)

ii) The approval number(s), or a statement that approval was granted by the named board(s)

iii) A statement that formal consent was obtained (must state whether verbal/written) OR the reason consent was not obtained (e.g. anonymity). NOTE: If child participants, the statement must declare that formal consent was obtained from the parent/guardian.].

2) Please upload all main figures as separate Figure files in .tif or .eps format. For more information about how to convert and format your figure files please see our guidelines:

https://journals.plos.org/plospathogens/s/figures

3) Thank you for stating that "Bulk RNA-seq datasets for DVG-B and A549-ACE2 infected cells are in the process of depositing to the Gene Expression Omnibus (GEO) database for public access." Please note that, though access restrictions are acceptable now, your entire data will need to be made freely accessible if your manuscript is accepted for publication. This policy applies to all data except where public deposition would breach compliance with the protocol approved by your research ethics board.

4) Please amend your detailed Financial Disclosure statement. This is published with the article. It must therefore be completed in full sentences and contain the exact wording you wish to be published.

1) State what role the funders took in the study. If the funders had no role in your study, please state: "The funders had no role in study design, data collection and analysis, decision to publish, or preparation of the manuscript."

5) Please ensure that the funders and grant numbers match between the Financial Disclosure field and the Funding Information tab in your submission form. Note that the funders and grants must be provided in the same order in both places as well.

6) Please provide a completed 'Competing Interests' statement, including any COIs declared by your co-authors. If you have no competing interests to declare, please state "The authors have declared that no competing interests exist". Otherwise please declare all competing interests beginning with the statement "I have read the journal's policy and the authors of this manuscript have the following competing interests:"

7) Some material included in your submission may be copyrighted. According to PLOS’s copyright policy, authors who use figures or other material (e.g., graphics, clipart, maps) from another author or copyright holder must demonstrate or obtain permission to publish this material under the Creative Commons Attribution 4.0 International (CC BY 4.0) License used by PLOS journals. Please closely review the details of PLOS’s copyright requirements here: PLOS Licenses and Copyright. If you need to request permissions from a copyright holder, you may use PLOS's Copyright Content Permission form.

Please respond directly to this email and provide any known details concerning your material's license terms and permissions required for reuse, even if you have not yet obtained copyright permissions or are unsure of your material's copyright compatibility. Once you have responded and addressed all other outstanding technical requirements, you may resubmit your manuscript within Editorial Manager.

Potential Copyright Issues:

Figure 3A.Please confirm whether you drew the images / clip-art within the figure panels by hand. If you did not draw the images, please provide (a) a link to the source of the images or icons and their license / terms of use; or (b) written permission from the copyright holder to publish the images or icons under our CC BY 4.0 license. Alternatively, you may replace the images with open source alternatives. See these open source resources you may use to replace images / clip-art:

- https://commons.wikimedia.org

- https://openclipart.org/.

Note: If the reviewer comments include a recommendation to cite specific previously published works, please review and evaluate these publications to determine whether they are relevant and should be cited. There is no requirement to cite these works unless the editor has indicated otherwise.

**Reviewers' Comments:**

Reviewer's Responses to Questions

**Part I - Summary**

Please use this section to discuss strengths/weaknesses of study, novelty/significance, general execution and scholarship.

Reviewer #1: This manuscript investigates the functional heterogeneity of defective viral genomes (DVGs) generated during SARS-CoV-2 infection. The authors identify two major genomic hotspots (A and B) for DVG formation and demonstrate that:

DVG abundance correlates with COVID-19 severity

DVGs from hotspot B (but not A) strongly induce type I interferon responses

Both DVG-A and DVG-B suppress wild-type virus replication

DVG-B generates dsRNA with distinct subcellular localization and enhanced immunostimulatory capacity

These findings suggest that genomic origin determines the immunological function of DVGs, providing insight into how DVGs contribute to both antiviral responses and disease severity.

Reviewer #2: n this manuscript, the authors follow-up on the analysis of Defective Viral Genomes (DVG) that were identified in a previous study. This previous study identified two dominant DVG, DVG-A and DVG-B. DVG-A was predominantly found in vitro with little/no detection in vivo.

This manuscript presents two different stories. The first story is the comparison of DVG- A and DVG-B, to define the differential role of these DVGs. However the relevance of DVG-A is not clear as detected to very low/undetectable levels in patient lung samples.

The second story is the role of DVG-B in viral infection and the mechanism of IFN induction. This aspect of the manuscript is novel and interesting given the in vivo relevance of DVG-B with detection in patient lung samples. However this aspect of the manuscript and mechanistic link to N expression is somewhat clouded by the loss of 4 viral proteins in DVG-B. The molecular linkage of N to RLR inhibition has been described before, (see critique and paper below), but there is still a novel story here. To my knowledge, this would be the first paper to demonstrate how N regulates IFN dynamics in the context of a viral infection.

Overall by combining these two stories, this manuscript is convoluted. For these reasons I would support this manuscript with a focus on just DVG-B and perform additional studies with a N deletion (or N mutant) virus. See below for more specific details.

Reviewer #3: Overall, this is a well-executed, thorough, and well-described study. The finding that DVGs with specific genetic architectures is a novel, interesting and potentially impactful finding affecting our understanding of host responses to SARS-CoV-2 as well as knowledge on how to deploy/design TIPs. Minor limitations to the study include the breath/diversity of DVG types studies. It would be interesting to characterize other specific DVGs comprising recombination junctions found the 'B' region of the DVG landscape rather than just one specific construct. But the findings of the study stand as is.

Though this is a complex and in-depth study, I have no comments or concerns with the data presented nor interpretations thereof. The studies include suitable controls and approaches that support the findings. I have only some minor comments to help on clarity of the figures/description:

In figure 2B/C, S1A and similar, it's a little unclear how cell point corresponds to a single cell with a specific DVG. Is the cells assigned according to the most abundant/prevalent DVG observed? Or, if multiple DVGs are found in cell (which seems likely), are multiple points (one for each observed DVG) plotted for a single cell?

I found the headings on Figure 5G a little confusing. I believe this represents the ratio of IFNB1 (for example) found in WT vs DVGB cell carrying cells, but as labelled this look like a plot of the ratio of Wt genomic RNA to DVG-B RNA.

The separation of the Wt sample data point in the PCA analysis in Figure S2A is concerning and may reduce the accuracy/power of the DE analyses presented in figure 5 and related. Is the dispersion/variability of these Wt data when using all 4 replicates higher than the other samples in this analysis? Or rather, are a small number of genes driving this separation (e.g. some cells might be in a later stage of infection and proceeding to CPE/death). Ideally, if feasible, these RNAseq libraries should be repeated. But since the Wt point do not co-cluster with other data points and that the findings are generally robust and consistent with expectations, this is only of minor concern and would be a 'good-to-have' rather than a core issue.

**********

**Part II – Major Issues: Key Experiments Required for Acceptance**

Please use this section to detail the key new experiments or modifications of existing experiments that should be absolutely required to validate study conclusions.

Generally, there should be no more than 3 such required experiments or major modifications for a "Major Revision" recommendation. If more than 3 experiments are necessary to validate the study conclusions, then you are encouraged to recommend "Reject".

Reviewer #1: (No Response)

Reviewer #2: Comparison of DVG-A vs DVG-B is a bit contrived. Method of synthesis of these two DVGs are very different and the level of RNA resulting from these will be massively different inside the host cell as DVG-B is replication competent and DVG-A is not. Also DVG-A appears largely to be an artifact of tissue culture as there is little/no detection in lung patient samples. Recommend to cut DVG-A comparison from the manuscript.

Authors attempt to ascribe the majority of IFN induction to the loss of N. This is supported by the rescue data and the previous description of N function (see above). However the conclusions with DVG-B are somewhat clouded as there is a loss of Orf7a/7b, Orf8, and N in DVG-B. To more directly assess the role of N in DVG-B gene regulation, the authors should create a KO virus with just N deleted (or active site mutations) but retaining expression of Orf7a/7b and Orf8. A complete repeat of all studies with the new virus is not necessary, but the authors should perform studies to validate their findings and link the IFN induction specifically to a loss of N.

Reviewer #3: (No Response)

**********

**Part III – Minor Issues: Editorial and Data Presentation Modifications**

Please use this section for editorial suggestions as well as relatively minor modifications of existing data that would enhance clarity.

Reviewer #1: 1. Mechanistic depth: What makes DVG-B more immunostimulatory?

The central conclusion is that DVG-B induces stronger IFN responses, but the mechanism remains somewhat descriptive.

• The authors show distinct dsRNA localization and IRF3 activation

• However, it is unclear which sensor(s) are responsible (e.g., MDA5 vs RIG-I)

Suggestion:

• Include or discuss:

o MAVS dependency (KO or inhibition)

o MDA5 vs RIG-I contribution

• Even discussion-level clarification would strengthen mechanistic insight

2. DVG abundance vs disease severity – causation vs correlation

The manuscript reports that DVG levels correlate with COVID-19 severity

However:

• Severe disease may simply reflect higher viral replication → more DVGs

Suggestion:

• Clarify this limitation explicitly in Discussion

• If possible:

o Normalize DVGs to viral load (Jfreq is mentioned but not emphasized enough)

• A short paragraph would greatly improve interpretation rigor

3. Physiological relevance of engineered DVGs

The authors construct representative DVG-A and DVG-B and test them functionally.

Concern:

• Do these constructs fully recapitulate natural DVG diversity?

Suggestion:

• Clarify:

o How representative these constructs are of endogenous DVG populations

• Possibly include:

o Sequence variability or structural predictions

4. Role of nucleocapsid (N) protein—interpretation needs refinement

The authors show:

• N complementation changes dsRNA localization

• But does not change IFN response

This is interesting but under-discussed.

❗ Key question:

Suggestion:

• Expand discussion:

o Is spatial organization uncoupled from sensing?

o Or is sensing already saturated?

This could be a conceptually important point

5. Replication vs immune activation trade-off

Both DVG-A and DVG-B suppress WT virus replication, but only DVG-B induces strong IFN.

Suggestion:

• Discuss:

o Are these two separable functions?

o Could DVG-A act as a “silent suppressor”?

Minor Comments

Clarity & Writing

• Some sections of the Introduction are slightly long—could be streamlined

• Define “hotspot A/B” earlier for clarity

Figures

• Figure legends are generally clear, but:

o Add schematic for DVG-A vs DVG-B structure (very helpful visually)

• In imaging data:

o Quantification should always accompany representative images (some already do well)

Methods

• Clarify:

o MOI consistency across experiments

o Whether IFN readouts are normalized per infected cell

Data Availability

• GEO deposition is “in progress”

Reviewer #2: The role of viral nucleocapsid protein has previously been described. Aloise et al 2023. https://doi.org/10.1371/journal.ppat.1011582. Specifically this previous publication demonstrated that N inhibits RLR pathway. This paper is not referenced or discussed at all, but could explain nearly all the phenotypes associated with DVG-B. Authors should put findings in context of this previous publication.

Reviewer #3: (No Response)

**********

PLOS authors have the option to publish the peer review history of their article (what does this mean?). If published, this will include your full peer review and any attached files.

If you choose “no”, your identity will remain anonymous but your review may still be made public.

**Do you want your identity to be public for this peer review?**  For information about this choice, including consent withdrawal, please see our Privacy Policy.

Reviewer #1: No

Reviewer #2: No

Reviewer #3: **Yes:**  Andrew Routh

[NOTE: If reviewer comments were submitted as an attachment file, they will be attached to this email and accessible via the submission site. Please log into your account, locate the manuscript record, and check for the action link "View Attachments". If this link does not appear, there are no attachment files.]

**Figure resubmission:**

While revising your submission, we strongly recommend that you use PLOS’s NAAS tool (https://ngplosjournals.pagemajik.ai/artanalysis) to test your figure files. NAAS can convert your figure files to the TIFF file type and meet basic requirements (such as print size, resolution), or provide you with a report on issues that do not meet our requirements and that NAAS cannot fix.  After uploading your figures to PLOS’s NAAS tool - https://ngplosjournals.pagemajik.ai/artanalysis, NAAS will process the files provided and display the results in the "Uploaded Files" section of the page as the processing is complete. If the uploaded figures meet our requirements (or NAAS is able to fix the files to meet our requirements), the figure will be marked as "fixed" above. If NAAS is unable to fix the files, a red "failed" label will appear above. When NAAS has confirmed that the figure files meet our requirements, please download the file via the download option, and include these NAAS processed figure files when submitting your revised manuscript.

**Reproducibility:**

To enhance the reproducibility of your results, we recommend that authors of applicable studies deposit laboratory protocols in protocols.io, where a protocol can be assigned its own identifier (DOI) such that it can be cited independently in the future. Additionally, PLOS ONE offers an option to publish peer-reviewed clinical study protocols. Read more information on sharing protocols at https://plos.org/protocols?utm_medium=editorial-email&utm_source=authorletters&utm_campaign=protocols

# Author comment: Author response to Decision Letter 1

**DOI:** 10.1371/journal.ppat.1014099.r002

29 Jul 2026

Attachment Submitted filename: Point-by-point responses.docx

# Peer review: Decision Letter 1

**DOI:** 10.1371/journal.ppat.1014099.r003

**Authors:** Holly Ramage, Michael Letko

29 Aug 2026

PPATHOGENS-D-26-00695R1

SARS-CoV-2 Defective Viral Genomes from Distinct Genomic Regions Drive Divergent Interferon Responses

PLOS Pathogens

Dear Dr. Sun,

Thank you for submitting your manuscript to PLOS Pathogens. As you will read, the reviewers are generally satisfied with your revision and responses.  However, Reviewer 1 has indicated some minor points that should be addressed. Therefore, we invite you to submit a revised version of the manuscript that addresses these points.

Please submit your revised manuscript by Oct 28 2026 11:59PM. If you will need more time than this to complete your revisions, please reply to this message or contact the journal office at plospathogens@plos.org. When you're ready to submit your revision, log on to https://www.editorialmanager.com/ppathogens/ and select the 'Submissions Needing Revision' folder to locate your manuscript file.

Please include the following items when submitting your revised manuscript:

* A letter that responds to each point raised by the editor and reviewer(s). You should upload this letter as a separate file labeled 'Response to Reviewers'. This file does not need to include responses to any formatting updates and technical items listed in the 'Journal Requirements' section below.

* A marked-up copy of your manuscript that highlights changes made to the original version. You should upload this as a separate file labeled 'Revised Manuscript with Track Changes'.

* An unmarked version of your revised paper without tracked changes. You should upload this as a separate file labeled 'Manuscript'.

If you would like to make changes to your financial disclosure, competing interests statement, or data availability statement, please make these updates within the submission form at the time of resubmission. Guidelines for resubmitting your figure files are available below the reviewer comments at the end of this letter.

As the corresponding author, your ORCID iD is verified in the submission system and will appear in the published article. PLOS supports the use of ORCID, and we encourage all coauthors to register for an ORCID iD and use it as well. Please encourage your coauthors to verify their ORCID iD within the submission system before final acceptance, as unverified ORCID iDs will not appear in the published article. Only the individual author can complete the verification step; PLOS staff cannot verify ORCID iDs on behalf of authors.

We look forward to receiving your revised manuscript.

Kind regards,

Holly Ramage

Academic Editor

PLOS Pathogens

Michael Letko

Section Editor

PLOS Pathogens

Sumita Bhaduri-McIntosh

Editor-in-Chief

PLOS Pathogens

orcid.org/0000-0003-2946-9497

Dominique Soldati-Favre

Editor-in-Chief

PLOS Pathogens

orcid.org/0000-0003-4156-2109

**Journal Requirements:**

If the reviewer comments include a recommendation to cite specific previously published works, please review and evaluate these publications to determine whether they are relevant and should be cited. There is no requirement to cite these works unless the editor has indicated otherwise.

[Note: HTML markup is below. Please do not edit.]

**Reviewers' Comments:**

Reviewer's Responses to Questions

**Part I - Summary**

Please use this section to discuss strengths/weaknesses of study, novelty/significance, general execution and scholarship.

Reviewer #1: This manuscript investigates the functional diversity of SARS-CoV-2 defective viral genomes (DVGs) and demonstrates that DVGs originating from two distinct genomic hotspots have markedly different biological activities. While both hotspot A and hotspot B DVGs suppress wild-type virus replication, only hotspot B strongly induces type I and III interferon responses, largely due to the absence of the nucleocapsid (N) protein, which alters viral dsRNA organization and enhances RIG-I-mediated innate immune activation. These findings provide new mechanistic insight into how naturally occurring coronavirus DVGs differentially regulate host antiviral immunity and viral pathogenesis.

Reviewer #2: Thank you for thoroughly addressing all comments/critiques. The conclusions of this manuscript are made stronger with this new data.

Reviewer #3: This is an important and interesting study. The author have addressed my original comments and concerns well.

**********

**Part II – Major Issues: Key Experiments Required for Acceptance**

Please use this section to detail the key new experiments or modifications of existing experiments that should be absolutely required to validate study conclusions.

Generally, there should be no more than 3 such required experiments or major modifications for a "Major Revision" recommendation. If more than 3 experiments are necessary to validate the study conclusions, then you are encouraged to recommend "Reject".

Reviewer #1: NA

Reviewer #2: None

Reviewer #3: (No Response)

**********

**Part III – Minor Issues: Editorial and Data Presentation Modifications**

Please use this section for editorial suggestions as well as relatively minor modifications of existing data that would enhance clarity.

Reviewer #1: Clarify the rationale for selecting representative DVG-A and DVG-B constructs. Although this point is discussed in the Discussion, it would be helpful to briefly explain in the Methods or Results why the outermost deletion boundaries were chosen and how well these constructs represent the diversity of naturally occurring DVGs.

Temper statements implying causality between DVG abundance and disease severity. The patient data demonstrate a positive association, but because DVG abundance also correlates with viral load, the manuscript should consistently emphasize that these observations are correlative rather than evidence that DVGs contribute directly to disease severity.

Clarify the statistical analysis for single-cell RNA-seq data. The manuscript would benefit from a clearer description of how cells containing multiple DVG junctions were handled and whether statistical comparisons accounted for repeated observations from the same cell.

Expand the discussion of RIG-I versus MDA5 recognition. While the new knockdown experiments support a predominant role for RIG-I, the possibility that MDA5 contributes at later stages of infection or recognizes different DVG-derived RNA species should be discussed more explicitly.

Improve figure readability. Several multi-panel figures (particularly Figures 5–9) contain numerous panels and small font sizes. Increasing font size and simplifying panel labels would improve readability.

Minor editorial corrections. A careful proofreading is recommended to correct several grammatical errors and improve wording consistency (e.g., "Interesting" instead of "Interestingly" in the Results, consistent formatting of IFN-β/IFNB1, and uniform use of "hotspot A" and "hotspot B" throughout the manuscript).

Reviewer #2: None

Reviewer #3: (No Response)

**********

PLOS authors have the option to publish the peer review history of their article (what does this mean?). If published, this will include your full peer review and any attached files.

If you choose “no”, your identity will remain anonymous but your review may still be made public.

**Do you want your identity to be public for this peer review?**  For information about this choice, including consent withdrawal, please see our Privacy Policy.

Reviewer #1: No

Reviewer #2: No

Reviewer #3: **Yes:**  Andrew Routh

[NOTE: If reviewer comments were submitted as an attachment file, they will be attached to this email and accessible via the submission site. Please log into your account, locate the manuscript record, and check for the action link "View Attachments". If this link does not appear, there are no attachment files.]

**Figure resubmission:**

While revising your submission, we strongly recommend that you use PLOS’s NAAS tool (https://ngplosjournals.pagemajik.ai/artanalysis) to test your figure files. NAAS can convert your figure files to the TIFF file type and meet basic requirements (such as print size, resolution), or provide you with a report on issues that do not meet our requirements and that NAAS cannot fix.

After uploading your figures to PLOS’s NAAS tool - https://ngplosjournals.pagemajik.ai/artanalysis, NAAS will process the files provided and display the results in the "Uploaded Files" section of the page as the processing is complete. If the uploaded figures meet our requirements (or NAAS is able to fix the files to meet our requirements), the figure will be marked as "fixed" above. If NAAS is unable to fix the files, a red "failed" label will appear above. When NAAS has confirmed that the figure files meet our requirements, please download the file via the download option, and include these NAAS processed figure files when submitting your revised manuscript.

**Reproducibility:**

To enhance the reproducibility of your results, we recommend that authors of applicable studies deposit laboratory protocols in protocols.io, where a protocol can be assigned its own identifier (DOI) such that it can be cited independently in the future. Additionally, PLOS ONE offers an option to publish peer-reviewed clinical study protocols. Read more information on sharing protocols at https://plos.org/protocols?utm_medium=editorial-email&utm_source=authorletters&utm_campaign=protocols

# Author comment: Author response to Decision Letter 2

**DOI:** 10.1371/journal.ppat.1014099.r004

8 Sep 2026

Attachment Submitted filename: Point-by-pont response_090726.docx

# Editor report: Decision Letter 2

**DOI:** 10.1371/journal.ppat.1014099.r005

**Authors:** Holly Ramage, Michael Letko

12 Sep 2026

Dear Assistant Professor Sun,

We are pleased to inform that your manuscript, "SARS-CoV-2 Defective Viral Genomes from Distinct Genomic Regions Drive Divergent Interferon Responses", has been editorially accepted for publication at PLOS Pathogens.

Before your manuscript can be formally accepted and sent to production, you will need to complete our formatting changes, which you will receive by email within a week. Please note that your manuscript will not be scheduled for publication until you have made the required changes.

IMPORTANT NOTES

(1) Copyediting and Proofreading: The corresponding author will receive a typeset proof for review, to ensure errors have not been introduced during production. Please review the PDF proof of your manuscript carefully, as this is the last chance to correct any errors. Please note that major changes, or those which affect the scientific understanding of the work, will likely cause delays to the publication date of your manuscript.

(2) Appropriate Figure Files: Please remove all name and figure # text from your figure files. Please also take this time to check that your figures are of high resolution, which will improve the readbility of your figures and help expedite your manuscript's publication. Please note that figures must have been originally created at 300dpi or higher. Do not manually increase the resolution of your files. For instructions on how to properly obtain high quality images, please review our Figure Guidelines, with examples at: http://journals.plos.org/plospathogens/s/figures.

We encourage you to use PACE (http://pace.apexcovantage.com/), a figure-checking tool which helps authors ensure all figures meet PLOS requirements so that figures can publish in the highest quality. PACE is a digital diagnostic and conversion tool for figure files. It will provide information about any failed check(s) and, if able, will automatically convert the figure into an acceptable file that passes quality checks.

To use PACE, you must first register as a user. Then, login and navigate to the UPLOAD tab, where you will find detailed instructions on how to use the tool.

(3) Striking Image: Please upload a striking still image to accompany your article if one is available (you can include a new image or an existing one from within your manuscript). Should your paper be accepted, this image will be considered for our monthly issue image and may also appear on our website to feature your article. Please upload this as a separate file, selecting "striking image" as the file type upon upload. Please also include a separate "Other" file with a caption, including credits and any potential copyright information. Please do not include the caption in the main article file. If your image is from someone other than yourself, please ensure that the artist has read and agreed to the terms and conditions of the Creative Commons Attribution License at http://journals.plos.org/plospathogens/s/content-license. Please note that PLOS cannot publish copyrighted images.

(4) Press Release or Related Media: If you or your institution will be preparing press materials for this manuscript, please inform our press team in advance at plospathogens@plos.org as soon as possible. We ask that you contact us within one week to plan ahead of our fast Production schedule. If you need to know your paper's publication date for related media purposes, you must coordinate with our press team, and your manuscript will remain under a strict press embargo until the publication date and time.

(5) Update your Profile Information: Now that your manuscript has been provisionally accepted, please log into Editorial Manager and update your profile, if needed. Go to http://www.editorialmanager.com/ppathogens, log in, and click on the "Update My Information" link at the top of the page. Please update your user information to ensure an efficient production and billing process.

(6) LaTeX users only: Our staff will ask you to upload a TEX file in addition to the PDF before the paper can be sent to typesetting, so please carefully review our Latex Guidelines http://journals.plos.org/plospathogens/s/latex in the meantime.

Thank you again for supporting Open Access publishing; we are looking forward to publishing your work in PLOS Pathogens.

Best regards,

Holly Ramage

Academic Editor

PLOS Pathogens

Michael Letko

Section Editor

PLOS Pathogens

Dominique Soldati-Favre

Editor-in-Chief

PLOS Pathogens

***********************************************************

# Editor report: Acceptance letter

**DOI:** 10.1371/journal.ppat.1014099.r006

**Authors:** Holly Ramage, Michael Letko

Dear Assistant Professor Sun,

We are delighted to inform you that your manuscript, "SARS-CoV-2 Defective Viral Genomes from Distinct Genomic Regions Drive Divergent Interferon Responses," has been formally accepted for publication in PLOS Pathogens.

We have now passed your article onto the PLOS Production Department who will complete the rest of the pre-publication process. All authors will receive a confirmation email upon publication.

Thank you again for supporting open-access publishing; we are looking forward to publishing your work in PLOS Pathogens.

Best regards,

Dominique Soldati-Favre

Editor-in-Chief

PLOS Pathogens
